# Structural and thermal evolution of the eastern Aar Massif: insights from structural field work and Raman thermometry

**DOI:** 10.1186/s00015-020-00381-3

**Published:** 2021-03-03

**Authors:** Lukas Nibourel, Alfons Berger, Daniel Egli, Stefan Heuberger, Marco Herwegh

**Affiliations:** 1 Institute of Geological Sciences, University of Bern, Baltzerstrasse 1+3, 3012 Bern, Switzerland; 2 Department of Earth Sciences, ETH Zurich, Sonneggstrasse 5, 8092 Zürich, Switzerland

**Keywords:** Aar Massif, Compressional deformation and exhumation, Relative timing of peak-metamorphism and deformation, Raman thermometry, Structural evolution, Steep reverse faults, Inherited normal faults

## Abstract

The thermo-kinematic evolution of the eastern Aar Massif, Swiss Alps, was investigated using peak temperature data estimated from Raman spectroscopy of carbonaceous material and detailed field analyses. New and compiled temperature-time constraints along the deformed and exhumed basement-cover contact allow us to (i) establish the timing of metamorphism and deformation, (ii) track long-term horizontal and vertical orogenic movements and (iii) assess the influence of temperature and structural inheritance on the kinematic evolution. We present a new shear zone map, structural cross sections and a step-wise retrodeformation. From $\text{ca.\;26\,Ma}$ onwards, basement-involved deformation started with the formation of relatively discrete NNW-directed thrusts. Peak metamorphic isograds are weakly deformed by these thrusts, suggesting that they initiated before or during the metamorphic peak under ongoing burial in the footwall to the basal Helvetic roof thrust. Subsequent peak- to post-metamorphic deformation was dominated by steep, mostly NNW-vergent reverse faults ($\text{ca.}$ 22–14 Ma). Field investigations demonstrate that these shear zones were steeper than $50^{\circ}$ already at inception. This produced the massif-internal structural relief and was associated with large vertical displacements (7 km shortening vs. up to 11 km exhumation). From 14 Ma onwards, the eastern Aar massif exhumed “en bloc” (i.e., without significant differential massif-internal exhumation) in the hanging wall of frontal thrusts, which is consistent with the transition to strike-slip dominated deformation observed within the massif. Our results indicate 13 km shortening and 9 km exhumation between 14 Ma and present. Inherited normal faults were not significantly reactivated. Instead, new thrusts/reverse faults developed in the basement below syn-rift basins, and can be traced into overturned fold limbs in the overlying sediment, producing tight synclines and broad anticlines along the basement-cover contact. The sediments were not detached from their crystalline substratum and formed disharmonic folds. Our results highlight decreasing rheological contrasts between (i) relatively strong basement and (ii) relatively weak cover units and inherited faults at higher temperature conditions. Both the timing of basement-involved deformation and the structural style (shear zone dip) appear to be controlled by evolving temperature conditions.

## Introduction

Collisional mountain belts form in response to convergent movements between tectonic plates and result from the closure and subduction of oceanic domains, followed by continent-continent collision. Such mountain belts typically involve passive continental margins, including basins and normal faults inherited from pre-orogenic extension (e.g., Beaumont et al. 2000; Bellahsen et al. 2012; Butler et al. 2006; Jackson 1980; Lacombe and Mouthereau 2002; Lafosse et al. 2016; Lemoine et al. 1989; Manatschal 2004; Marshak et al. 2000; Mohn et al. 2012). In mountain belts, the structural style reflects the manner by which convergence is accommodated in the crust and lithospheric mantle of the colliding plates (e.g., Jammes and Huismans 2012; Mouthereau et al. 2013). Principally, the tectonic deformation style depends on two main parameters: (i) the large-scale geodynamic framework and (ii) the rheology and spatial distribution of involved rock types (basement units and sedimentary cover). Structural styles are commonly described as two conceptual end-members. In the case of a strong and dense lithosphere, contractional deformation is typically restricted to a narrow décollement within the weak sedimentary cover (thin-skinned style, Rodgers 1949) whereas the underlying basement is underthrusted without significant internal deformation (Burov and Yamato 2008). A weak and buoyant lithosphere on the other hand favours thick-skinned deformation affecting large parts of the crust, which leads to basement uplift associated with an increased pure-shear component at the crustal scale (Capitanio et al. 2010; Cloos 1993; Mouthereau et al. 2013). Coeval thin-skinned and thick-skinned deformation is commonly observed within different regions of the same mountain belt (e.g., Bauville and Schmalholz 2015; Nemčok et al. 2013; Pfiffner 2017).

Mouthereau et al. (2013) demonstrated that the strength of the lithosphere and thus the distribution of strain depends to a first order on the thermotectonic age (i.e., the time elapsed since the last pre-orogenic rifting event). Another significant factor is the inherited extensional passive margin structure (e.g., Lafosse et al. 2016). The reactivation of inherited normal faults is mainly a function of the steepness and orientation of the fault plane, the friction along the fault plane and its frictional strength relative to the surrounding rock mass (Bauville and Schmalholz 2015; Bellahsen et al. 2012; Buiter and Pfiffner 2003; Buiter et al. 2009; Butler et al. 2006; Jammes and Huismans 2012; Lacombe and Bellahsen 2016).

The competence contrast between the relatively strong basement versus weak sediments and inherited faults may change through time. At shallow crustal levels (i.e., at low temperatures) this contrast is considered high (Lafosse et al. 2016). At higher metamorphic conditions, this contrast appears to decrease, which may inhibit the reactivation of inherited faults and the formation of detachments between basement and cover units (Bellahsen et al. 2012; Lafosse et al. 2016).

The Aar Massif (European Alps) is the easternmost and largest of a belt of basement domes commonly referred to as the External Crystalline Massifs (ECMs, e.g., Bellahsen et al. 2012; Schmid et al. 2004). In these Massifs, basement-involved deformation in the footwall of thin-skinned thrust sheets caused rapid and highly localised exhumation (e.g., Bellanger et al. 2015; Boutoux et al. 2016; Fox et al. 2016; Glotzbach et al. 2010, 2011b; Herwegh et al. 2020; Rolland et al. 2008, 2009; Sanchez et al. 2011; Schwartz et al. 2017; Vernon et al. 2008). The ECMs are therefore key places for studying (i) geodynamic processes, (ii) the mechanical behaviour of basement and cover units and (iii) the role of inherited passive margin structures during collisional deformation.

In this study, we examine the Alpine ($\sim 34\,\text {Ma}$ to present) thermo-kinematic evolution of the eastern Aar Massif by combining detailed field analyses with the quantitative assessment of peak temperature ($T_p$) based on Raman spectroscopy on carbonaceous material (RSCM). The high spatial resolution of 47 new and 335 compiled $T_p$ data allows peak metamorphic isograds to be analysed in map and section view. The exposed metamorphic pattern and structural observations are used (i) to establish the relative timing of metamorphism and deformation and (ii) to put upper temperature bounds to the temperature conditions prevailing during deformation. A new kinematic scenario for the tectonic evolution of the eastern Aar Massif is proposed in the form of five line- and area-balanced scaled profile sketches, in which new and published temperature-time constraints highlight the vertical component of deformation. We thereby especially address the following three aspects: (i) The Aar Massif exposes basement and cover units from sub-greenschist facies in the north to upper greenschist facies in the south (e.g., Bousquet et al. 2012; Nibourel et al. 2018). How did the mechanical behaviour of contrasting lithologies (i.e., overall granitoid basement versus calcite-dominated cover sediments) and the overall structural style evolve as a function of metamorphic conditions? (ii) What is the present-day position and orientation of inherited passive margin structures such as normal faults and related basins? To what degree were they reactivated during Alpine inversion and how did they influence subsequent compressional deformation? (iii) Finally, we assess if Alpine compressional structures were passively rotated into their present-day orientation or if they were already relatively steep at inception. This has important implications for the estimation of shortening vs. uplift ratios during compressional deformation.

## Geological setting and previous work

### Geological overview

**Fig. 1:**
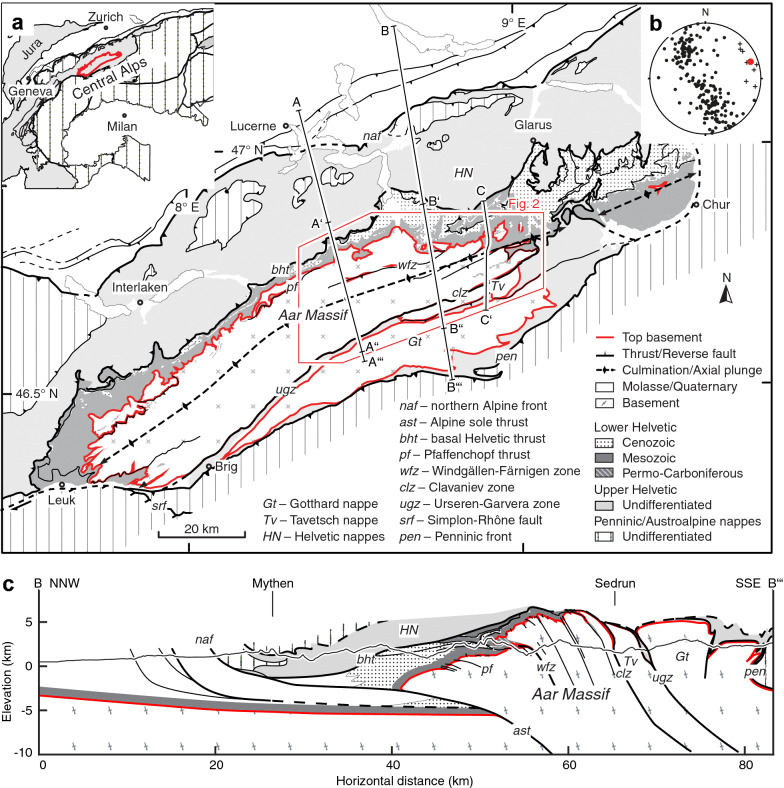
**a** Simplified tectonic map of the Aar Massif (modified after Schmid et al. 2004). Traces of cross sections A–A′′′ (Susten pass), cross sections B–B′′′ (Reusstal), cross section C–C′ (Tödi) and the study area (red polygon) are highlighted. Inset modified after Schmid et al. (2004). **b** Stereographic projection shows poles to bedding (black dots, 227 measurements) and axes to early folds (black crosses, 8 measurements) from the sedimentary cover of the eastern Aar Massif (Schmidt, lower hemisphere). The mean easterly axial plunge of $070^{\circ }/10^{\circ }$ (red circle, plunge-azimuth/plunge) represents a cylindrical best fit through all poles to bedding. **c** Cross section B–B′′′ (in part after Brückner and Zbinden 1987; Pfiffner et al. 2011)

The Central Alps are classically viewed as the result of approximately NW-SE directed convergence between the European and the Adriatic plates, leading to the relative southward subduction of the Penninic ocean, followed by the post-35 Ma continent-continent collision (Froitzheim et al. 1996; Handy et al. 2010; Schmid et al. 1996, 2017). The Aar Massif is a WSW-ENE-trending elongate dome exposing the pre-Triassic basement of the European continental margin in the footwall of stacked thrust sheets including, in ascending order, the Helvetic, Penninic and Austroalpine nappes (Fig. 1, Schmid et al. 1996). It is characterised by a structural relief (i.e. top basement topography) increasing from an elevation of -6 km below the North Alpine foreland to estimated 6 km in the central Aar Massif over less than 20 km horizontal distance (see cross section B–B$'''$, Fig. 1c) and an associated increase in the exposed metamorphic grade from sub-greenschist facies in the north to greenschist facies in the south (e.g., Bambauer et al. 2009; Frey and Ferreiro Mählmann 1999; Herwegh et al. 2017). The elevation of top basement below the North Alpine foreland is constrained by seismic profiles (Pfiffner et al. 1997). In cross section B–B$'''$, the eroded top basement surface above the Aar Massif was projected parallel to the mean easterly axial plunge of the eastern Aar Massif, which leads to the exposure of higher tectonic levels towards the east (Fig. 1, Hitz and Pfiffner 1994; Nibourel et al. 2018). The Aar Massif basement, together with its Carboniferous to Cenozoic autochthonous and allochthonous sedimentary cover (summarised as the Lower Helvetic), is separated from the overlying Helvetic nappes (Upper Helvetic) by the basal Helvetic thrust (Milnes and Pfiffner 1977). Passive folding and tilting of the basal Helvetic thrust indicates that internal deformation of the Aar Massif mostly post-dates the emplacement of the Helvetic and tectonically higher nappes (Fig. 1c, see also Milnes 1974). To the south, the Aar Massif is bound by the Urseren-Garvera or the Clavaniev zones representing two major detachments (Fig. 1). Both zones contain strongly deformed Mesozoic and Permo-Carboniferous sediments and especially the Clavaniev zone locally also includes basement fragments (e.g., Berger et al. 2017a; Gisler 2018). The sediments of the Urseren-Garvera and the Clavaniev zones are interpreted to represent the cover of the Gotthard nappe and the Aar Massif, respectively, which is mostly based on their stratigraphic polarity (e.g., Bonanomi et al. 1992; Gisler 2018; Wyss 1986, Figs. 1 and 2). Inside the Aar Massif, Permo-Carboniferous to Cenozoic sediments are locally preserved in WSW-ENE-trending wedges, synclines or graben-related structures (e.g., Burkhard 1988; Berger et al. 2016; Pfiffner 2015). The most prominent occurrence of massif-internal sediments is the Windgällen-Färnigen zone, separating the northern Aar Massif from the main body of the Aar Massif to the south (Fig. 1, Funk et al. 1983; Gisler 2018; Heim and Heim 1916; Kammer 1985; Labhart et al. 2015; Morgenthaler 1921; Schenker 1980).

The timing of burial of the Aar Massif domain below the advancing orogenic wedge is constrained by the Rupelian (34–28 Ma) age of the syn-orogenic Matt Formation (Lu et al. 2018; Menkveld-Gfeller et al. 2016) representing the youngest sediments to be deposited onto the Aar Massif domain. The age of peak metamorphism is estimated at ca. 22–17 Ma based on isotopic ages from syn-kinematic minerals and thermal considerations (e.g., Berger et al. 2017b; Challandes et al. 2008; Rolland et al. 2009; Wehrens et al. 2017), or slightly earlier (27–19 Ma) as indicated by a subset of relatively old but Alpine reset zircon fission track and zircon helium ages from the northern Aar Massif (Herwegh et al. 2020; Michalski and Soom 1990; Nibourel 2019; Wangenheim 2016). Estimated convergence rates between Europe and Adria decreased from $\sim 13\,\text {mm/year}$ in the Oligocene to $\sim 2\,\text {mm/year}$ during the Neogene (Schmid et al. 1996; Handy et al. 2010). Basement-involved crustal thickening initiated at ca. 22 Ma (e.g., Challandes et al. 2008; Rolland et al. 2009) and caused highly localised and rapid exhumation in the Aar Massif and other ECMs during the Neogene (e.g., Fox et al. 2016; Fügenschuh and Schmid 2003; Glotzbach et al. 2010; Michalski and Soom 1990; Reinecker et al. 2008; Vernon et al. 2008, 2009). This is also confirmed by a significant increase in the present-day crustal thickness from the weakly deformed North Alpine foreland to the Aar Massif (Pfiffner et al. 1997; Rosenberg and Kissling 2013; Schmid et al. 2017; Wagner et al. 2012).

### Structural evolution and tectonic architecture

Alpine collisional structures of the Aar Massif have been studied over more than one century, especially in the central Aar Massif (e.g., Baltzer 1880; Franks 1968a; Heim 1921; Kammer 1989; Labhart 1966; Milnes and Pfiffner 1977; Rohr 1926; Steck 1968; Wehrens et al. 2017). Along the Haslital transect, an early steep to sub-vertical foliation and associated ductile shear zones with down-dip stretching lineations were recognised (e.g., Steck 1968; Wehrens et al. 2017). Based on isotopic ages of syn-kinematic sheet silicates (Berger et al. 2017b; Challandes et al. 2008; Rolland et al. 2009) and of monazites from associated Alpine clefts (Bergemann et al. 2017; Bergemann 2017; Janots et al. 2012; Rauchenstein-Martinek 2014; Ricchi et al. 2019) the age of shearing was estimated at $\sim$ 22–14 Ma. In the southern central Aar Massif, thermodynamic calculations suggest shearing at a temperature of $450\,^{\circ }\text{C}$ and at a pressure of 6.5 kbar (Challandes et al. 2008; Goncalves et al. 2012). Early ductile shear zones are reactivated and overprinted by oblique to dextral strike-slip retrograde shear zones. Syn-kinematic sheet silicates and Alpine cleft monazites associated with these overprinting shear zones yield younger ages (14–10 Ma, Bergemann et al. 2017; Berger et al. 2017b; Challandes et al. 2008; Janots et al. 2012; Pleuger et al. 2012; Rauchenstein-Martinek 2014; Rolland et al. 2009). At the exhumed northern massif front, an array of NNW-vergent brittle-ductile thrusts (Berger et al. 2017b) were active after approximately 12 Ma, as indicated by an age jump in zircon fission track ages across the zone of most intense thrusting (Herwegh et al. 2020). Deformation in the Aar Massif and other ECMs is inferred to be kinematically linked to thrusting along the Subalpine Molasse (Boyer and Elliott 1982; Burkhard 1990; Burkhard and Sommaruga 1998; Mock et al. 2020; Pfiffner et al. 1997, 1990, 2011; von Hagke et al. 2012), where thrusting initiated in the Late Oligocene (Kempf et al. 1999; Schlunegger et al. 1997) and lasted until at least 5 Ma (von Hagke et al. 2012). In the basement of the eastern Aar Massif, the distribution of strain and associated kinematics have not been extensively studied. In most of the northern and eastern Aar Massif, no major detachments are observed between basement and cover units (Pfiffner 2015). In contrast, large portions of the sedimentary cover were sheared off the crystalline basement of the southern Aar Massif and transported to the north during an early phase of deformation (i.e., Cavestrau décollement and other early Lower Helvetic thrust sheets highlighted on Fig. 3a, b, Brückner and Zbinden 1987; Mair et al. 2018; Pfiffner 1978).

Contrasting kinematic/geodynamic models have been proposed to explain the structural development of the Aar Massif, mainly depending on whether the main steep Alpine foliation and associated shear zones were interpreted as (i) thrusts, which were passively “back-rotated” in the hanging wall of younger active thrusts (Burkhard 1988, 1999), (ii) an axial plane foliation (e.g., Burkhard 1999; Krayenbuhl and Steck 2009; Steck 1968) mainly reflecting coaxial deformation (Choukroune and Gapais 1983) or (iii) S-block up dominated shear zones accommodating sub-vertical tectonic movements driven by lower crustal delamination and buoyancy (Herwegh et al. 2017). This is also reflected by a wide range of shortening estimates, as demonstrated by Burkhard (1999). All models presented in Herwegh et al. (2020) involve fundamental assumptions regarding the mechanical behaviour of basement rocks under greenschist facies conditions, which are, in the absence of markers in the basement of the central Aar Massif, difficult to constrain. Here, we carefully reassess the structural role of this foliation and investigate its steepness at inception by studying its geometrical relationship to inherited normal faults and the basement-cover contact.

### Pre-Triassic units

**Fig. 2:**
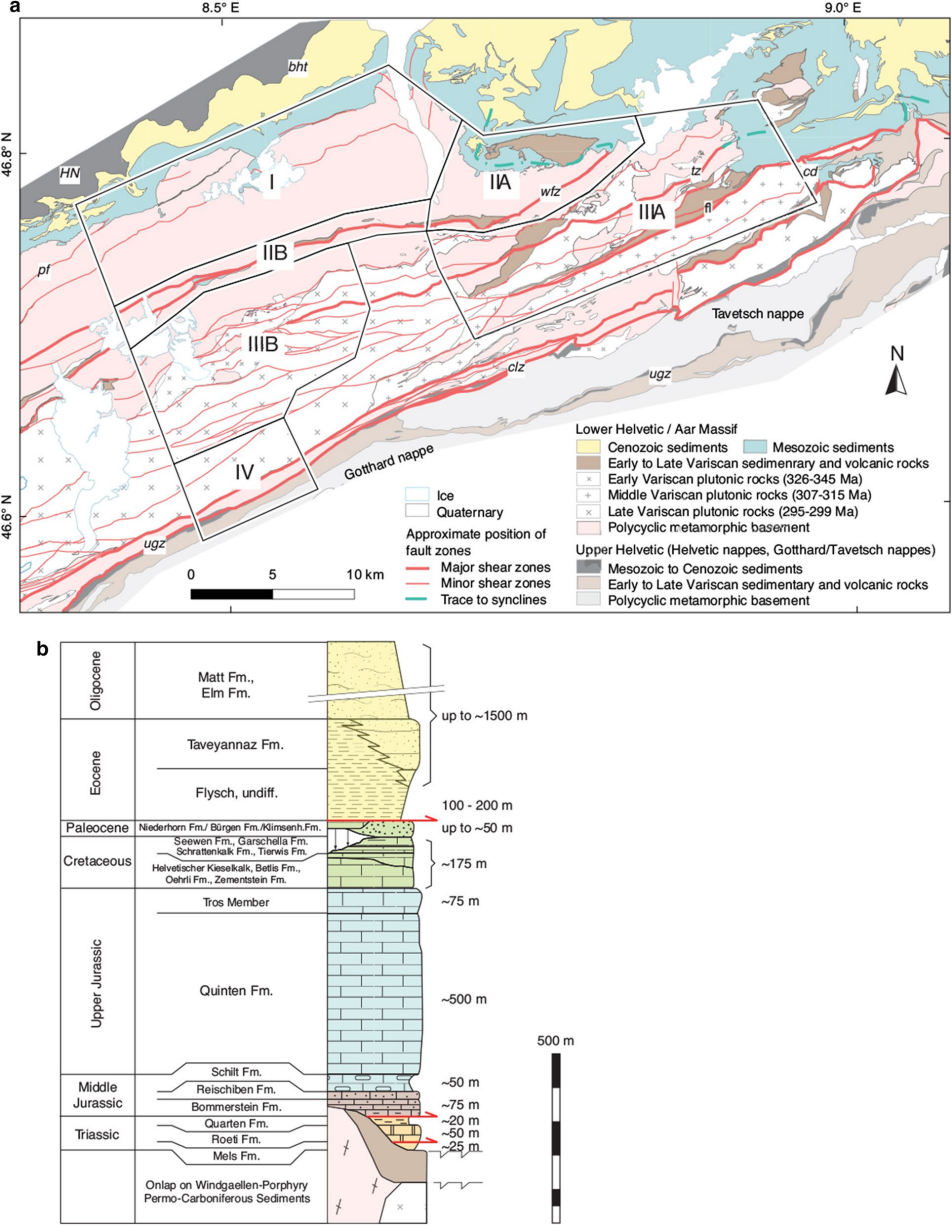
**a** Geological map of the Aar Massif (modified after Berger et al. 2016) showing the simplified pattern of major (thickness $>\,20\,\text {m}$) and minor (2–20 m) Alpine basement shear zones and associated synclines in the cover units (partly modified from Baumberger 2015; Pfiffner 1978). See Fig. 1a for location of map. *HN*—Helvetic nappes; *bht*—basal Helvetic thrust; *pf*—Pfaffenchopf thrust; *wfz*—Windgällen-Färnigen zone; *tz*—Tödi zone; *fl*—Frisal line; *cd*—Cavestrau décollement; *clz*—Clavaniev zone; *ugz*—Urseren-Garvera zone. We subdivide the study area into four subregions I–IV: I—northern Aar Massif; IIA—Windgällen area; IIB—Färnigen area; IIIA—Tödi area; IIIB—Göscheneralp area; IV—southern Aar Massif. **b** Summarised stratigraphic column of the eastern Aar Massif (modified after Gautschi et al. 2008; Pfiffner 2015)

Polycyclic metamorphic basement units make up the majority of the Aar Massif (Fig. 2a). These very heterogeneous but mostly gneissic metamorphic rocks are of Late Proterozoic to Early Paleozoic age and show a different degree of retrograde alteration (e.g., Abrecht 1994; Berger et al. 2017a; Von Raumer et al. 1993, and references therein). Berger et al. (2017a) subdivided these rocks into seven mostly NNW-striking zones with internally coherent characteristics. These units were intruded by early to post-Variscan granitoids of essentially Carboniferous to Permian age (see Berger et al. 2017a, for a review, Fig. 2a). Late- to post-Variscan intrusives such as the central Aare granite only record Alpine metamorphism and compressional deformation structures. Plutonic activity was accompanied by subaerial deposition of clastic and volcanoclastic sediments into isolated basins or graben-related structures, essentially during Carboniferous and Permian times (Eugster 1951; Franks 1968a, b; Labhart 1977; Oberhänsli et al. 1988; Schaltegger et al. 2003; Schenker 1987; Schenker and Abrecht 1987). These sediments locally contain organic carbon, making them suitable target lithologies for RSCM analysis.

### Mesozoic to Cenozoic sedimentary cover and passive margin evolution

The pre-Triassic units described above, in this study summarised as basement, are covered by a up to 1000 m thick Mesozoic carbonate shelf sequence consisting mainly of limestones with minor dolomites, marls, shales and sandstones deposited during the development of the European continental margin (Heim 1921; Pfiffner et al. 2011; Rohr 1926; Trümpy et al. 1980). In the following, we describe the most important lithostratigraphic units of the Lower Helvetic sequence (Fig. 2b, http://www.strati.ch). Mesozoic sedimentation initiated with the deposition of a transgressive basal sandstone or conglomerate (Mels Formation) onto the crystalline basement (e.g., Gisler et al. 2007). These deposits are covered by the Röti Formation consisting mostly of massive dolomite. In the absence of the Upper Triassic Quarten Formation and the entire Lower Jurassic sequence, the dolomites are often covered by the Middle Jurassic dark shales, marls, silicious limestones and sandstones (Bommerstein and Reischiben Formations, Dollfus 1965). The fine-grained limestones of the Upper Jurassic (Schilt and Quinten Formations) are the most prominent units of the Lower Helvetic stratigraphy. In the eastern Aar Massif, the Cretaceous sediments, mostly comprising limestones, siliceous limestones, marls and sandstones, were partly eroded during a phase of pre-Eocene exposure (Pfiffner et al. 2011). The transition to the Cenozoic is marked by a large hiatus, as also highlighted by the infilling of weathering products into karst pockets of the Upper Cretaceous to Upper Jurassic limestones (i.e. depending on the level of erosion), also known as “Siderolithic” (Herb 1983). The Cenozoic sequence is mainly characterised by sedimentation of syn-orogenic clastic sediments (Dielforder et al. 2015, 2016; Kempf and Pfiffner 2004; Lihou and Allen 1996; Lihou 1996a, b; Pfiffner et al. 2011; Pfiffner 2015).

Stratigraphic and structural observations (i.e., lateral thickness and facies variations, syn-sedimentary normal faulting) indicate different pulses of rift-related normal faulting starting mainly in the Early Jurassic (e.g., Pfiffner 1993; Trümpy et al. 1980). In the Helvetic nappes, continued rifting during the Cretaceous and possibly even the Cenozoic is documented (e.g., Cardello and Mancktelow 2014; Hänni and Pfiffner 2001; Kempf and Pfiffner 2004; Pfiffner 2015). Mostly S-vergent normal faults and related grabens or half-grabens separated basement blocks such as the Gastern Massif and the Aar Massif (Burkhard 1988; Herwegh and Pfiffner 2005) as well as the Aar Massif and the Gotthard nappe (Trümpy et al. 1980) already during the passive margin evolution. The footwall blocks to these normal faults were uplifted and exposed, as indicated by the absence of Early Jurassic sediments in the Aar Massif domain (i.e. “Alemannisches Land”, Pfiffner 2015). The position and role of inherited normal faults during the development of the eastern Aar Massif has not been previously studied.

### Existing constraints on the grade of Alpine metamorphism

The general metamorphic pattern is well mapped in the Central Alps by the presence or absence of key index minerals or assemblages (e.g., Bousquet et al. 2008; Frey and Ferreiro Mählmann 1999; Niggli and Niggli 1965), fluid inclusion compositions (Frey 1980) as well as illite crystallinity (Breitschmid 1982; Rahn et al. 1995). In the Lower Helvetic, there is a general increase in metamorphic grade from sub-greenschist facies in the north to greenschist facies in the south. A metamorphic discontinuity is observed across the basal Helvetic thrust, where the metamorphic grade increases from the footwall (Lower Helvetic) to the hanging wall (Upper Helvetic, Breitschmid 1982; Ebert et al. 2008; Herwegh and Pfiffner 2005). In the northern and eastern Aar Massif, the exact pattern of metamorphism is not completely resolved. This is mainly due to the presence of polycyclic metamorphic units and the difficulty in distinguishing between different metamorphic cycles in these rocks (e.g., Berger et al. 2017a). Alpine metamorphism was investigated based on the transition from microcline to sanidine during Alpine deformation (Bambauer et al. 2005), or by mapping the onset of dynamic quartz recrystallization (Bambauer et al. 2009). Frey et al. (1976) mapped the Alpine biotite-in isograd, which is located south of the Windgällen-Färnigen zone and continues to the Tödi area. Thermo-barometric data on syn-kinematic peak metamorphic mineral assemblages (Challandes et al. 2008; Goncalves et al. 2012) and fluid inclusions (Frey 1980; Schenker 1980) confirm the general north-south increase of metamorphic grade from ca. $250\,^{\circ }\text{C}$ / 1.3–2.5 kbar in the north to more than $450\,^{\circ }\text{C}$/6.5 kbar in the south. More recently, peak temperature conditions were quantitatively estimated using RSCM (Berger et al. 2020; Beyssac et al. 2002; Erne 2014; Girault et al. 2020; Hafner 2016; Lahfid et al. 2010; Mair et al. 2018; Nibourel et al. 2018; Negro et al. 2013; Wiederkehr et al. 2011) and calcite-graphite or calcite-dolomite thermometry (Herwegh and Pfiffner 2005). These studies mostly focused on the western Aar Massif, the Urseren-Garvera zone, the Glarus area or the Penninics. The metamorphic pattern of the northern and eastern Aar Massif was so far only partly constrained.

## Approach and methodology

### Field work, fault zone map and cross sections

We mapped Alpine fault zones along three massif-perpendicular transects, approximately following cross sections A–A′′′, B–B′′′ and C–C′ (Fig. 1a). Between these transects, the lateral continuation of shear zones was estimated based on remote sensing data (high-resolution aerial-photography and digital elevation models), closely following the procedure described by Baumberger (2015). Published and unpublished geological information from maps, cross sections and tunnel data were compiled to complement and compare with our results (see Table S1 in Additional file 1 for a complete list of sources).

A compiled geological map covering the entire Aar Massif (Berger et al. 2016) and the Helvetic nappes (Pfiffner et al. 2011) was used as a basis for both the shear zone map and the cross sections. The shear zone map presented in Fig. 2a builds on published shear zone maps from the central Aar Massif (Baumberger 2015; Herwegh et al. 2020; Rolland et al. 2009; Steck 1968; Wehrens et al. 2017).

**Fig. 3:**
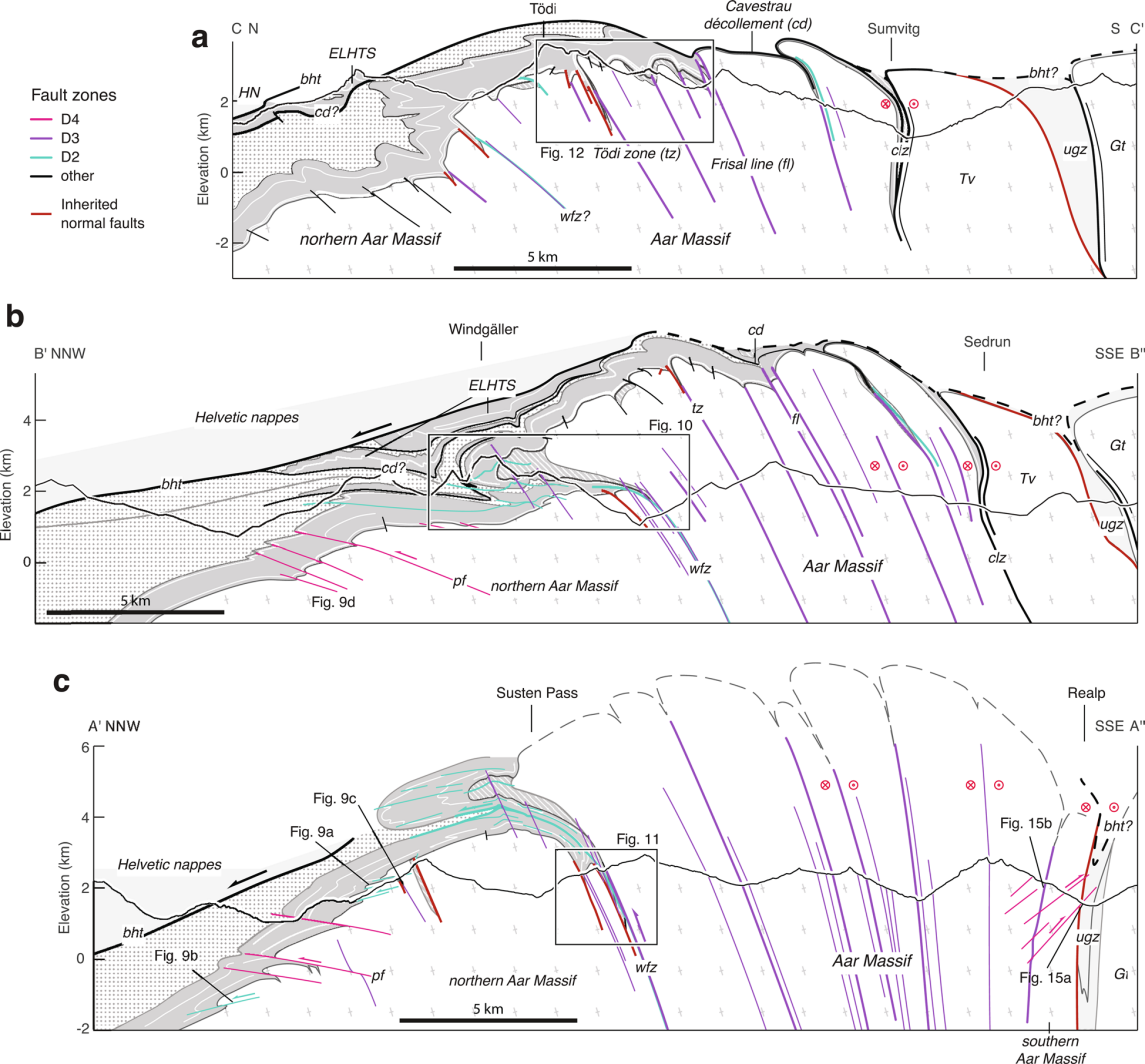
Three stacked cross sections through the eastern Aar Massif. Section traces are shown on Fig. 1a. *ELHTS* — Early Lower Helvetic thrust sheets. Legend and abbreviations as in Fig. 1a. **a** Section A′–A′′ is adapted from Nibourel et al. (2018). Coordinates: A′ $46.8492^{\circ }\text {N}/8.4274^{\circ }\text {E}$, A′′ $46.5916^{\circ }\text {N}/8.5181^{\circ }\text {E}$; Projection parallel to the fold axes: $070^{\circ }/10^{\circ }$ (plunge-azimuth/plunge). **b** Section B′-B′′ is in part after Brückner and Zbinden (1987) and Pfiffner et al. (2011). Coordinates: B′ $46.92282^{\circ }\text {N}/8.71097^{\circ }$E, B′′ $46.64687^{\circ }\text {N}/8.77070^{\circ }\text {E}$.; Projection parallel to the fold axes: $070^{\circ }/14^{\circ }$ (plunge-azimuth/plunge). **c** Section C–C′ is modified from Böhm (1986). Coordinates: C $46.8900^{\circ }\text {N}/8.9010^{\circ }\text {E}$, C′ $46.6627^{\circ }\text {N}/8.9047^{\circ }\text {E}$; Section trace C–C′ additionally runs through the mountain tops of Tödi: $46.8112^{\circ }\text {E}/8.9148^{\circ }\text {N}$, Stoc Grond: $46.8003^{\circ }\text {E}/8.9143^{\circ }\text {N}$ and Stoc Pign: $46.7862^{\circ }\text {E}/8.9072^{\circ }\text {N}$. Projection parallel to the fold axes: $065^{\circ }/10^{\circ }$ (plunge-azimuth/plunge)

Cross section A′–A′′ (Fig. 3c) and associated references are presented in Nibourel et al. (2018). In cross section B–B′′′ (Fig. 1) and its close-up view B′–B′′ shown on Fig. 3b), the Helvetic and Penninic nappes were adapted from existing cross sections (e.g., Brückner and Zbinden 1987; Pfiffner 1993, 2011). The position and orientation of basement shear zones are based on unpublished geological reports related to the nearby Gotthard NEAT tunnel, detailed field maps (Ambühl et al. 2008; Brückner and Zbinden 1987) as well as on our own observations. Cross section C–C′ (Fig. 3a) is partly redrawn after Böhm (1986). Based on our field observations, we included structural details regarding inherited normal faults and adapted the position and orientation of Alpine shear zones. The geometries of the Punteglias and Trun submassifs and the overturned Cavestrau nappe are adapted from Käch (1972), Pfiffner (1978) and Pfiffner (1993). The geometry of the Lower Helvetic thrust-slices and the Upper Helvetic nappes in the north are based on the projection of geological surface data from published geological (Berger et al. 2016) and tectonic (Pfiffner et al. 2011) maps (see Table S1 in Additional file 1).

Top basement and higher tectonic units were projected into the cross sections parallel to the regional easterly axial plunge of the eastern Aar Massif (the mean value for the study area is estimated at $070^{\circ }/10^{\circ }$ (plunge-azimuth/plunge), Fig. 1b). Detailed projection information is given in the caption to Fig. 3. The same projection was used to project $T_p$ data into the cross sections. Such a projection assumes a cylindrical geometry. This is certainly an oversimplification and thus associated with some uncertainties, especially in the case of section A′–A′′ (Fig. 1c), in which the basement-cover contact is projected over horizontal distances larger than 25 km. Owing to structural complexities (i.e., non-cylindricity), a few (< 10%) sample positions had to be adjusted by a maximum of 200 m. Adjustments were made to ensure that all data points are located within the correct stratigraphic unit and prior to the calculation of paleo-isotemperature contour lines, so they do not significantly influence the resulting temperature pattern and our interpretation.

Recognising Alpine compressional structures in the polycyclic metamorphic basement is very challenging. This is mainly due to the presence of pre-Alpine foliations and deformation structures, which were possibly active under pre-Alpine metamorphic conditions similar to those during Alpine deformation. An Alpine age of deformation is indicated if shear zones propagate from the polycyclic metamorphic basement into the Mesozoic cover or into mono-metamorphic late- or post-Variscan magmatic rocks. The basement-cover contact provides an excellent marker allowing us (i) to quantify absolute displacements and (ii) to independently constrain the overall kinematics of basement shear zones. This is a significant advantage with respect to the central Aar Massif, where the basement-cover contact is mostly eroded.

### RSCM analysis

RSCM estimates the metamorphic peak temperature ($T_p$) by quantifying the crystallinity of carbonaceous material in metasedimentary rocks, independently of diagnostic mineral assemblages and the extent of retrograde alteration (e.g., Beyssac et al. 2002; Lahfid et al. 2010). The method is based on the temperature-dependent increase of structural organization in organic matter towards the graphite high-temperature end-member (Beyssac et al. 2002). RSCM measurements were performed in situ on uncovered and polished thin sections at the Raman Laboratory of the Institute of Geological Sciences (University of Bern). The lab used is equipped with an Olympus BX41100x confocal microscope, a Peltier-cooled CCD detector (Andor Technology) and an air-cooled Nd-YAG laser (Compass 315 M, coherent, 20 mW) having a beam spot of approximately $1\,\upmu \text {m}$ diameter and a wavelength of 532.12 nm. The software LabSpec 4.14 of HORIBA Jobin-Yvon was used to start the measurements. A silicon standard was measured to check both calibration and signal intensity at the start of each session. Acquisition time varied between 20 and 120 seconds, depending on the signal to noise ratio. In order to avoid artefacts, the signal was accumulated over two to four accumulation cycles. Raman spectra were acquired between 500 and $2200\,\text {cm}^{-1}$ to insure that both, base line and all necessary first order Raman bands could be recognised (Tuinstra and Koenig 1970). Between 10 to 63 individual spots per sample were analysed, depending on (i) the availability of suitable graphitic particles and (ii) on the range of spectra observed during the first 10 measurements.

Thin sections were prepared and analysed according to the procedure described in Lünsdorf et al. (2014). RSCM measurements can be affected by several analytical mismatches. Therefore, we closely followed the analytical procedure described in Beyssac et al. (2002) and Beyssac and Lazzeri (2012). An automated, randomised and iterative curve-fitting approach (Lünsdorf et al. 2014; Lünsdorf and Lünsdorf 2016) was applied, which is based on a $T_p$ calibration described in Lünsdorf et al. (2017). This calibration is sensitive in a temperature field from $240$ to $600\,^{\circ }\text{C}$. Calibration-related absolute errors are in the order of $\pm\, 40\,^{\circ }\text{C}$. The performance of the automated curve-fitting was quality-checked for each spectrum. Insufficiently fitted spectra were discarded. The temperature distribution of each sample was assessed individually (see $T_p$ histograms on Figures S1-S4 in Additional file 1). In samples for which individual $T_p$ measurements were distributed normally around one central peak, the mean temperature value was plotted (see also Table S2, Figures S1-S4, Additional file 1). In many of these samples, narrow sample-internal distributions and equivalent estimates in neighbouring samples indicate that relative $T_p$ differences can be resolved down to $\pm\, 20\,^{\circ }\text{C}$ (Nibourel et al. 2018). Some samples yielded broad, bimodal or more complex $T_p$ distributions. In such samples, higher temperature estimates might be due to the presence of detrital higher metamorphic graphite (e.g., Galy et al. 2008) or indicate partly increased structural ordering of the carbonaceous material due to aseismic shear (e.g., Kedar et al. 2020). In both cases, the lowest local $T_p$ maximum is most likely to represent Alpine peak metamorphism. At the presence of bimodal $T_p$ signals or high-temperature outliers, we therefore interpreted the local maximum with the lowest temperature to reflect Alpine peak metamorphism. Such samples are highlighted by one asterisk in Additional file 1: Table S2 and on Figures S1-S4. This procedure is also justified by relatively good agreement with neighbouring less complex samples. To avoid potential effects of shear heating and/or strain (e.g., Barzoi 2015; Kedar et al. 2020; Kirilova et al. 2018; Kuo et al. 2017), we measured weakly deformed samples whenever possible.

Our sampling mainly focused on the parautochthonous sedimentary cover of the Aar Massif. In the absence of major detachments between basement and cover units, $T_p$ measurements from the cover should represent a lower bound for the maximum temperature reached in the underlying basement. Samples include silicate-dominated clastic sediments and calcite-dominated metasediments of Carboniferous to Cenozoic age (see Table S2, Additional file 1). In order to reduce the potential bias related to variations in the types of carbonaceous material in different stratigraphic units, we sampled the dark shales of the Middle Jurassic (Mols Member) whenever possible.

## Field data

The Aar Massif region has a complex and polyphase deformation history (e.g., Krayenbuhl and Steck 2009; Steck 1968; Wehrens et al. 2017). We start by presenting a new shear zone map of the eastern Aar Massif and three updated cross sections. We then describe the overall structural framework subdivided into five deformation phases D1 to D5. This numbering reflects the relative chronology based on overprinting relationships and geometrical arguments and is not necessarily meant to define discrete phases associated with changing kinematics.

### Shear zone map

Our shear zone map (Fig. 2a) highlights a heterogeneous distribution of Alpine strain in the crystalline basement. Strain is localised along a network of mostly ductile ENE to E trending anastomosing shear zones. Basement shear zones can be traced into pinched “synclines” at the basement-cover contact. The position of remotely sensed shear zones was only partly verified in the field. Significant uncertainties are to be expected at a large distance from the basement-cover contact and with increasing distance from the mapped transects (cross sections A–A′′′, B–B′′′ and C–C′, Fig. 1a), especially in the polycyclic metamorphic basement units. In the southeastern Aar Massif region, the pattern of Alpine shear zones is poorly constrained. This is partly due to poor outcrop conditions in the area, but also due to the scarcity of published and own structural observations. A more detailed shear zone map including field measurements and a reliability assignment can be found in Nibourel (2019).

### Cross sections

**Fig. 4:**
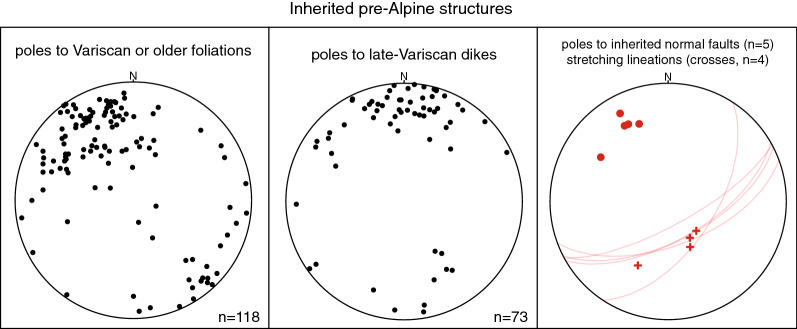
Structural data related to inherited pre-collisional structures (Schmidt, lower hemisphere). n: number of measurements

**Fig. 5:**
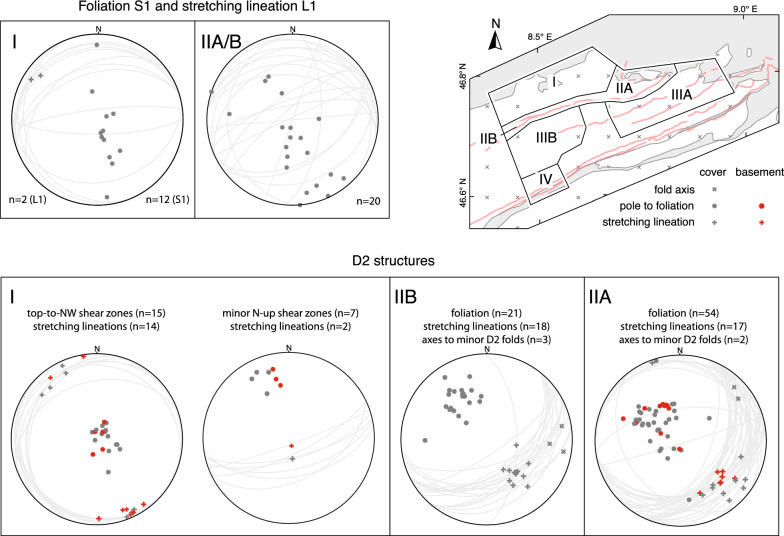
Structural data related to D1 and D2. Stereographic projections of poles to foliations, lineations and fold axes are given for each subregion. n: number of measurements. Where appropriate, the projections are coded for lithologies (see legend)

**Fig. 6:**
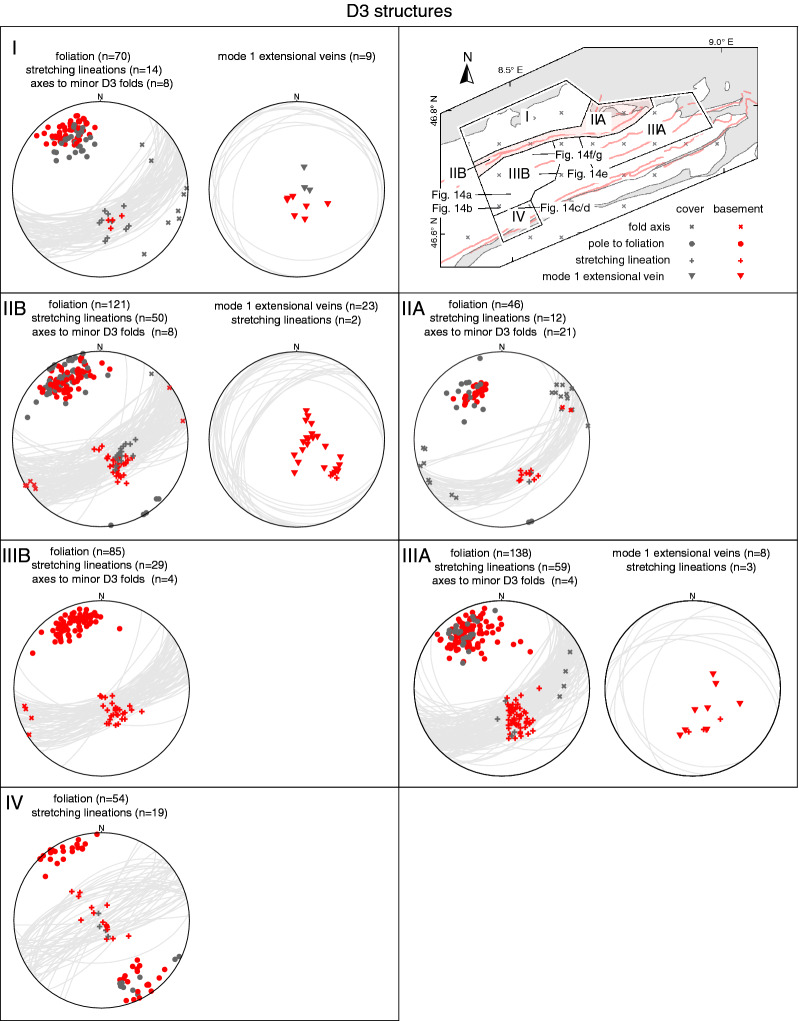
Structural data related to D3. For each subregion stereographic projections of poles to planes, lineations and fold axes are given. n: number of measurements. Where appropriate, the projections are coded for lithologies (see legend)

**Fig. 7:**
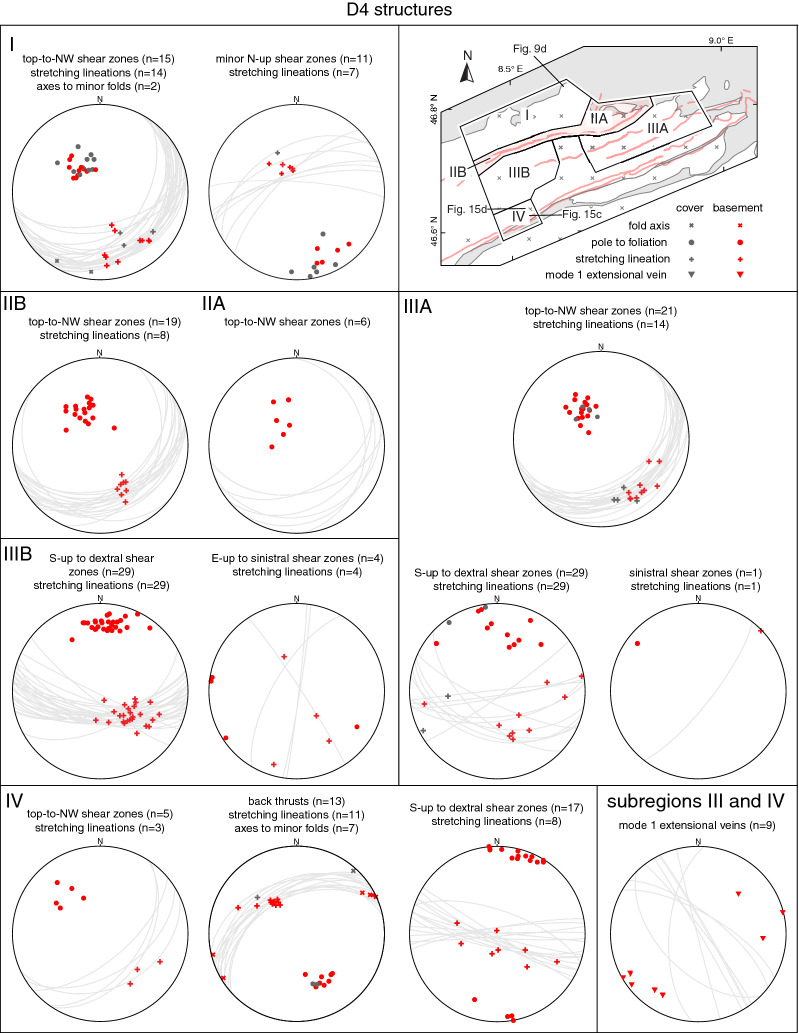
Structural data related to D4. For each subregion stereographic projections of poles to planes, lineations and fold axes are given. n: number of measurements. Where appropriate, the projections are coded for lithologies (see legend)

**Fig. 8:**
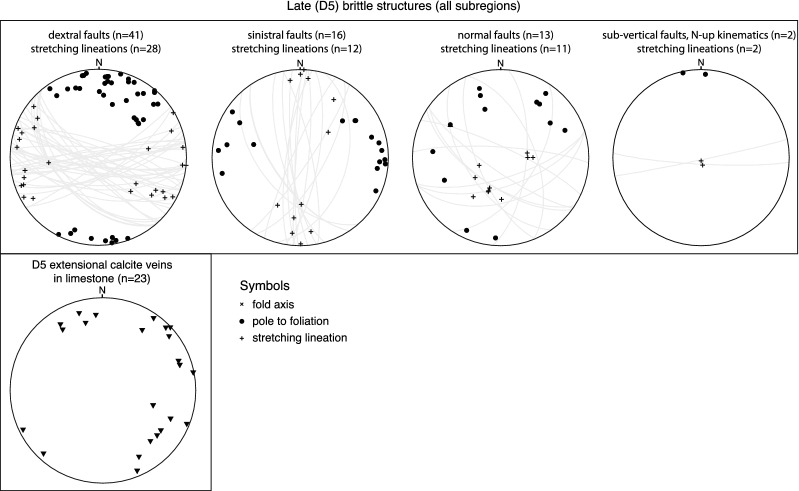
Stereographic projection of late brittle faults and post-foliation late mode 1 extensional calcite veins (cover units only). n: number of measurements

Three cross sections (A′–A′′, B′–B′′ and C–C′) are shown on Fig. 3, in descending structural order from east to west. The main Alpine shear zones and associated foliations are coloured after deformation phase D1 to D4. The estimated maximum elevation of top basement increases from ca. 3 km in the east (cross section C–C′, Fig. 3a) to 8 km in the west (cross section A′–A′′, Fig. 3c), which is consistent with an increasingly steep north-northwesterly dip of top basement at the northern massif front from east to west. This corresponds to an eroded thickness of up to 5 km of Aar Massif basement above the present-day topography, which is significantly more than previously estimated (Pfiffner et al. 2011). A rather high eroded top basement elevation above the Aar Massif is also indicated (i) by a gradual E-W increase in massif width (in orogen-perpendicular direction) (ii) and by an along-strike increase of the exposed metamorphic grade towards the centre of the Aar Massif (e.g., Nibourel et al. 2018). However, it has to be expected that the easterly axial plunge of top basement decreases towards its culmination point situated in the central Aar Massif. Therefore, the projection parallel to the overall regional fold axis used in this study might somewhat overestimate the elevation of top basement, especially in cross section A′–A′′ (Fig. 3). In contrast, in the top basement topography map shown by Pfiffner et al. (2011), top basement is placed right above the highest peaks of the Aar Massif. Based on the above considerations, this is likely to underestimate the true elevation of the eroded top basement horizon.

### Structural observations and interpretation

In the following, structural observations related to D1 to D5, including pre-collisional inherited structures, are described from north to south and shown in stereographic projections (Schmidt net, lower hemisphere, Figs. 4, 5, 6, 7 and 8). Where necessary, structural data were subdivided into structurally consistent subregions (Fig. 2a). Table 1 shows an attempt to correlate D1 to D5, as used in this study, with previously described deformation phases west (Burkhard 1988; Herwegh et al. 2020; Labhart 1966; Mair et al. 2018; Rolland et al. 2009; Steck 1968; Wehrens et al. 2017), east (Funk et al. 1983; Milnes and Pfiffner 1977) and south (Wyss 1986) of the study area. A table containing all field measurements is available in Nibourel (2019). Thermochronology data indicate that the timing of collisional deformation along the strike of the Aar Massif was diachronous (Nibourel 2019). Correlating deformation phases is therefore difficult, especially along the strike of the massif. The correlation shown on Table 1 is mainly based on fault kinematics, strike, dip and overprinting relationships. Indications for the metamorphic grade during deformation, such as syn-kinematic index minerals, were also considered. Pre-D1 deformation phases in more internal units such as the Upper Helvetic (e.g., Milnes and Pfiffner 1977) and the Penninic (e.g., Steck 1984; Wiederkehr et al. 2008) nappes are not considered here (Fig. 3a, b).

**Table 1:** Attempt to correlate deformation phases D1 to D5 from this study with previous work (Burkhard 1988; Brückner and Zbinden 1987; Funk et al. 1983; Pfiffner 2015; Milnes and Pfiffner 1977; Rolland et al. 2009; Schenker 1980; Wehrens et al. 2017; Wyss 1986)

Domain	References	Deformation phases				
Eastern Aar Massif	This study	D1	D2	D3	D4	D5
	Funk et al. (1983), Schenker (1980), Brückner and Zbinden (1987)	Griessstock and Kammlistock nappe emplacement, Helvetic Nappe emplacement, Hochfulen phase	Windgällen phase	Schwarzstöckli phase	–	–
	Milnes and Pfiffner (1977)	Cavestrau phase?	Calanda phase?	Calanda phase? Ruchi phase	–	–
Central Aar Massif	Wehrens et al. (2017), Herwegh et al. (2020)	–	–	Handegg phase	Oberaar phase, Pfaffenchopf phase	Gadmen faults
	Rolland et al. (2009)	–	–	Stage 1	stage 2, stage 3?	Stage 3?
	Burkhard (1988), Pfiffner (2015)	Kiental phase?	Kiental phase	Grindelwad phase	Grindelwad phase	–
Urseren-Garvera zone	Wyss (1986)	D1	D2	D3	Post-D3	–

#### Inherited pre-collisional structures

Pre-Alpine foliations are often preserved, especially in the northern Aar Massif (i.e., subregion I on Fig. 2a, Lehmann 2008). They are mostly steep to sub-vertical, show a large variation in strike from the mean strike of $160^{\circ }$ (Fig. 4) and often form a high angle to the overlying basal Mesozoic strata. Basic and felsic dykes are widespread in the crystalline basement and were commonly reactivated as shear zones during subsequent Alpine deformation (Schneeberger et al. 2017, 2018; Wehrens et al. 2016, 2017). They are mostly steep to sub-vertical and show a large variation in strike (Fig. 4). A number of inherited normal faults were identified. The most prominent examples are the Färnigen (Fig. 3b, c) and the Tödi normal faults (Fig. 3a). Both normal faults dip to the SSE with angles between 65 and $80^{\circ }$. Associated half-graben geometries can still be recognised.

#### D1 structures

The first discernible Alpine deformation structure is a cleavage (S1). S1 is only preserved in parautochthonous limestones or marls, especially in the north of the Aar Massif, where it was weakly overprinted by subsequent deformation. S1 is sub-parallel to the bedding (S0) of the strata and mostly strikes WSW-ENE, with a large variation in the dip angle (Fig. 5). Where preserved, associated stretching lineations trend NW-SE (Fig. 5). In the basement, no structures related to D1 shearing were recognised. D1 is presumably related to the burial of the Aar Massif domain in the footwall to the Helvetic nappes or to the emplacement of the early Lower Helvetic thrust sheets (i.e., Cavestrau phase, Käch 1972; Pfiffner 1978, see Cavestrau décollement and other early Lower Helvetic thrust sheets (ELHTS) on cross sections C–C′ and B′–B′′, Fig. 3a, b, see also Table 1).

#### D2 structures

D2 is mainly characterised by NNW-directed shearing affecting both basement and cover units (Fig. 5). D2 structures are only preserved in the northern Aar Massif and along the Windgällen-Färnigen zone. The relationship to overprinting structures is best established along the Windgällen-Färnigen zone (Fig. 5, subregion II). South of the Windgällen-Färnigen zone, D2 structures are mostly obliterated by subsequent deformation.

**Fig. 9:**
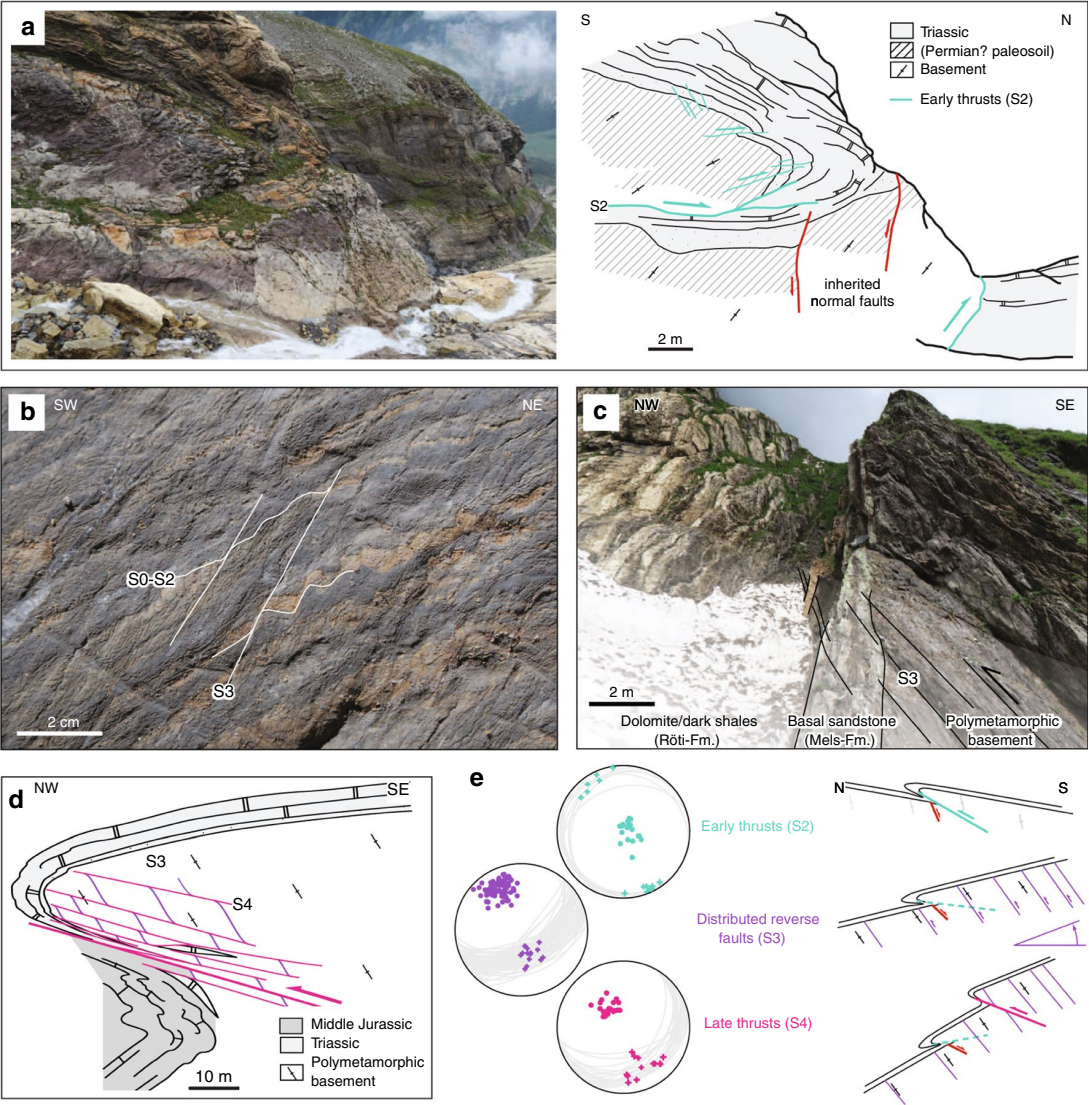
**a** Early N-dipping NNW-vergent thrust (S2, coordinates: $46.7833^{\circ }\text {N}/8.4474^{\circ }\text {E}$, see Fig. 3c for location). **b** Steep SE-dipping spaced crenulation cleavage S3 in the Middle Jurassic limestones, overprinting a pronounced S0–S2 composite foliation (coordinates: $46.6523^{\circ }\text {N}/8.1714^{\circ }\text {E}$, see Fig. 3c for location). **c** Steep main foliation (S3) in both Triassic marly beds (left) and their crystalline substratum (right). The sub-vertical orientation of the stratigraphic contact indicates significant SSE-block up movements along S3 (coordinates: $46.7587^{\circ }\text {N}/8.4027^{\circ }\text {E}$, see Fig. 3c for location). (**d**) Sketch of an array of post-S3 NNW-vergent thrusts (S4) at the basement-cover interface near Erstfeld (coordinates: $46.8409^{\circ }\text {N}/8.6264^{\circ }\text {E}$, see Figs. 3d and 7 for location). **e** (left) Structural measurements related to NNW-vergent shearing during D2–D4. Poles to fault planes (circles) and associated stretching lineations (crosses) are highlighted. (right) Schematic sketch illustrating the geometric relationship between S2 and S4 at the basement-cover contact during progressive deformation and block rotation associated with S3 at the northern massif front

In the northern Aar Massif, sub-horizontal to gently NNW-dipping brittle-ductile D2 faults (in the following also referred to as S2) dissect the crystalline basement and its sedimentary cover (Fig. 3, section A′–A′′, Fig. 5, subregion I). The kinematics are consistently top-to-the-NNW, as indicated by offsets along the basement-cover contact (Fig. 9a) or by calcite, quartz or chlorite slickenfibres on fault surfaces. S2 intersects the basal Triassic strata at a low angle ($10^{\circ }-25^{\circ }$, Fig. 9a). Displacements are in the order of sub-centimetres to several kilometres. Arrays of D2 faults lead to fold-like geometries at the basement-cover contact and to overturned stratigraphy in the upper limb of tight synclines (Fig. 9a). In the calcite-dominated Jurassic strata, S2 forms a ductile foliation and tight to isoclinal asymmetric folds.

**Fig. 10:**
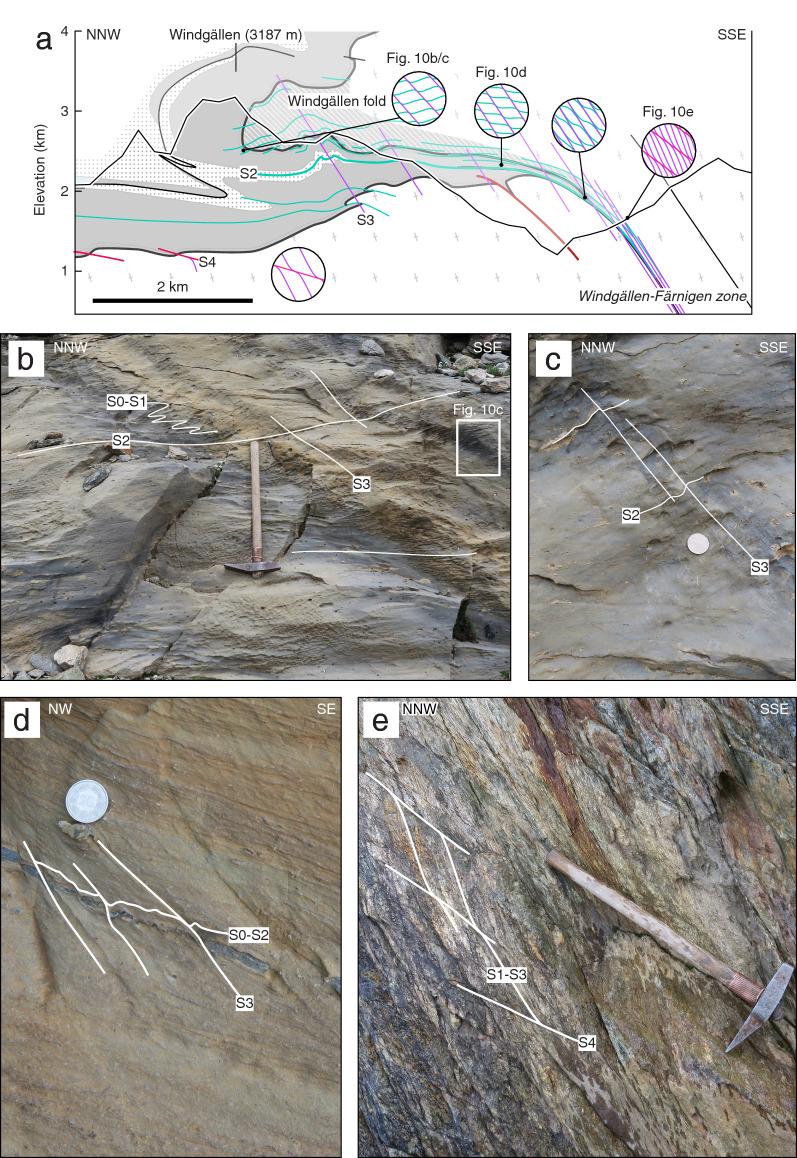
**a** Close-up view of section B′–B′′ (see Fig. 3 for location) showing the Windgällen fold-and-thrust structure. Legend as on Figure 1. Schematic sketches highlight the crosscutting relationships for the three main tectonic foliations S2 to S4 as well as overall changes in their relative spacing at different positions of the structure. **b** Well-developed sub-horizontal axial foliation cleavage S2 in Middle Jurassic strata (coordinates: $46.8015^{\circ }\text {N}/8.8267^{\circ }\text {E}$). **c** Close-up view of Fig. 5b showing a weakly developed steeper crenulation cleavage (S3) overprinting S0–S2. **d** Middle Jurassic siliceous limestone with a pronounced S3 crenulation cleavage overprinting the still dominating S0–S2 composite foliation (coordinates: $46.7982^{\circ }\text {N}/8.7441^{\circ }\text {E}$). **e** Basement shear zone in the Windgällen-Färnigen zone. A steep main foliation (S1–S3 composite) is overprinted by discrete top-to-the NNW shear bands (S4) (coordinates: $46.7191^{\circ }\text {N}/8.4630^{\circ }\text {E}$)

**Fig. 11:**
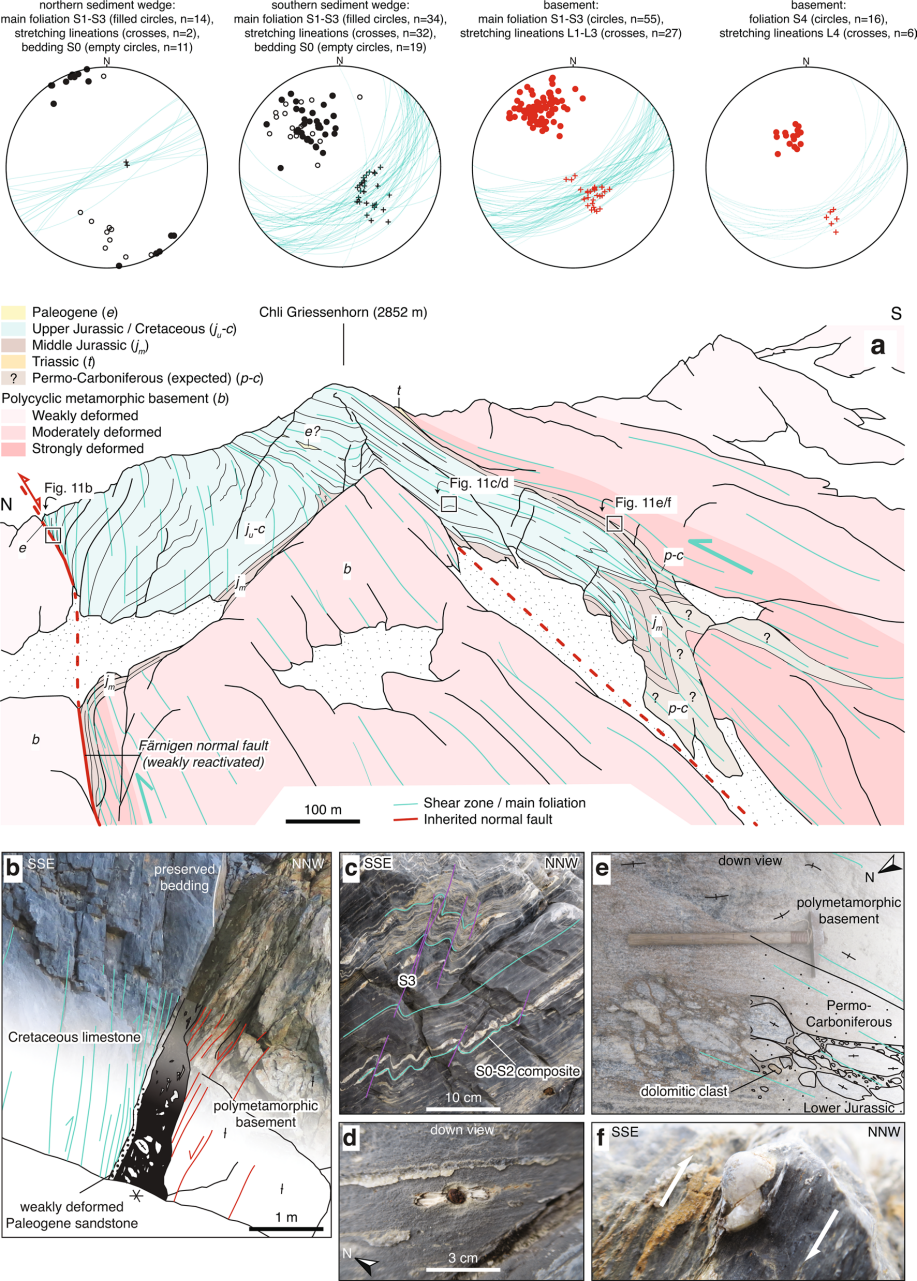
**a** Profile-like view of the Windgällen-Färnigen zone and structural data. Location is given in Fig. 3c. **b** Färnigen normal fault. Strongly foliated (highlighted as green lines) Cretaceous limestones and a $\sim 10\,\text {cm}$ thick band of weakly deformed Nummulite-bearing Paleogene sandstone are directly adjacent to a fault breccia (black). The gneisses adjacent to the normal fault are overprinted by cataclasites indicating S-block down movement (highlighted as red lines) and are only weakly overprinted by younger Alpine deformation. The Färnigen normal fault dips $70^{\circ }$ SSE (coordinates: $46.7303^{\circ }\text {N}/8.4740^{\circ }\text {E}$) **c** S3 overprinting an intense S0–S2 composite foliation (coordinates: $46.7281^{\circ }\text {N}/8.4814^{\circ }\text {E}$). **d** Well-developed stretching lineation in fine-grained limestone and pressure shadow around a pyrite concretion in the main foliation surface (S0–S3 composite, coordinates: $46.7291^{\circ }\text {N}/8.4850^{\circ }\text {E}$). **e** Overturned stratigraphic contacts between Carboniferous, Mesozoic sediments and the basement. The presence of dolomite clasts indicates a (Middle?) Jurassic or younger age for the basal conglomerate (Coordinates: 46.7269$^{\circ }\text {N}/8.4830^{\circ }\text {E}$). **f** Centimetre-sized shear band type fragmented quartz porphyroclast indicating top-to-the NNW shearing during D2 (coordinates: $46.7269^{\circ }\text {N}/8.4830^{\circ }\text {E}$)

NNW-dipping D2 faults appear to be restricted to the northern massif front, where top basement dips $30^{\circ }$ (cross section B′–B′′, Fig. 3b) to $40^{\circ }$ (cross section A′–A′′, Fig. 3c) NNW. We interpret these faults as early thrusts, which were passively rotated from originally south-southeasterly dip to their present day NNW-dipping orientation, as sketched on Fig. 9e. S2 is locally overprinted by a significantly steeper crenulation cleavage S3 (Fig. 9b). This relationship is best observed along the Windgällen-Färnigen zone, in which D2 and D3 were previously described as Windgällen and Schwarzstöckli phases (Figs. 10 and 11, Brückner and Zbinden 1987; Funk et al. 1983). Labhart (1966) described an early foliation with very similar characteristics in the limestone-dominated sediments associated with the Pfaffenchopf thrust (Fig. 3c).

A second minor set of discrete sub-vertical to steeply SSE dipping brittle-ductile faults, which is always associated with a N-block up sense of shear, is also attributed to D2. It possibly forms a conjugate set with the sub-horizontal faults described above. The two sets of faults intersect at a mean angle of $80^{\circ }$ (Fig. 5).

The Windgällen-Färnigen zone represents the most prominent D2 structure of the study area (see Fig. 3b, c and close-up view on Fig. 10a). In the Windgällen area (Region IIA on Fig. 5), Carboniferous-Permian volcanic and volcanoclastic rocks form the core of a kilometre-scale NW-verging recumbent anticline, also known as the Windgällen anticline (Figs. 10 and 11, Funk et al. 1983; Ramsay et al. 1983; Schenker 1980; Spillmann 2011). The Windgällen anticline is underlain by a pinched syncline containing Mesozoic to Cenozoic strata. The dominant structural element along the Windgällen-Färnigen zone is a lower greenschist facies axial plane cleavage S2 affecting both basement and cover units (Fig. 5). It is most tightly spaced along the lower overturned limb of the Windgällen anticline and can be traced into a steeply SSE dipping decametre-wide basement shear zone (Fig. 10a, see Baker 1964; Funk et al. 1983; Heim 1887; Tan 1969, 1976, for a quantitative assessment of strain). Axes to minor D2 folds plunge to the ENE with an angle of $5^{\circ }-15^{\circ }$. We interpret the easterly plunge of D2 fold axes to reflect passive tilting during the subsequent development of the Aar Massif dome. Figure 10B shows S2 overprinting earlier planar fabrics (i.e., a composite foliation S0-S1 in Middle Jurassic strata). S2 is associated with a well-developed mineral stretching lineation L2, which trends NW-SE (Fig. 5). Northwest-directed shearing is indicated (i) by outcrop scale shear sense criteria (see Fig. 11f for an example), (ii) the vergence of D2 folds and (iii) by displacements up to 5 km recorded along top basement (Fig. 3b, c). In the Windgällen region (Fig. 5, subregion IIA), S2 is sub-horizontal to moderately north-dipping. Towards the south and towards the west, S2 bends into a progressively steep SSE dip (Fig. 5, subregion IIB, Fig. 10a–d). This large variation in orientation might be (i) due to subsequent deformation, especially during D3 (see below) and/or (ii) partly reflect a ramp-flat geometry.

Similar observations of southward steepening early foliations and sediment wedges are known from the Doldenhorn (Burkhard 1988; Krayenbuhl and Steck 2009; Mair et al. 2018) and Morcles nappes (Ramsay et al. 1983) in the Central and Western Alps (i.e., Kiental phase in Table 1, Burkhard 1988; Mair et al. 2018; Pfiffner 2015). Furthermore, D2 structures appear to correlate with what is referred to as Calanda phase in the Lower Helvetic east of the study area (Milnes and Pfiffner 1977).

#### D3 structures

D3 is mainly characterised as a well-developed greenschist facies schistosity S3, which is associated with heterogeneously distributed ductile shearing affecting basement and cover units. Overall, D3 structures are the dominant structural element in the study area. The intensity of S3 increases from north to south (Fig. 3). In the northern Aar Massif (Fig. 6, subregion I), S3 is weakly developed and often reactivates pre-existing Variscan or older foliations, which here represent the dominant fabric.

**Fig. 12:**
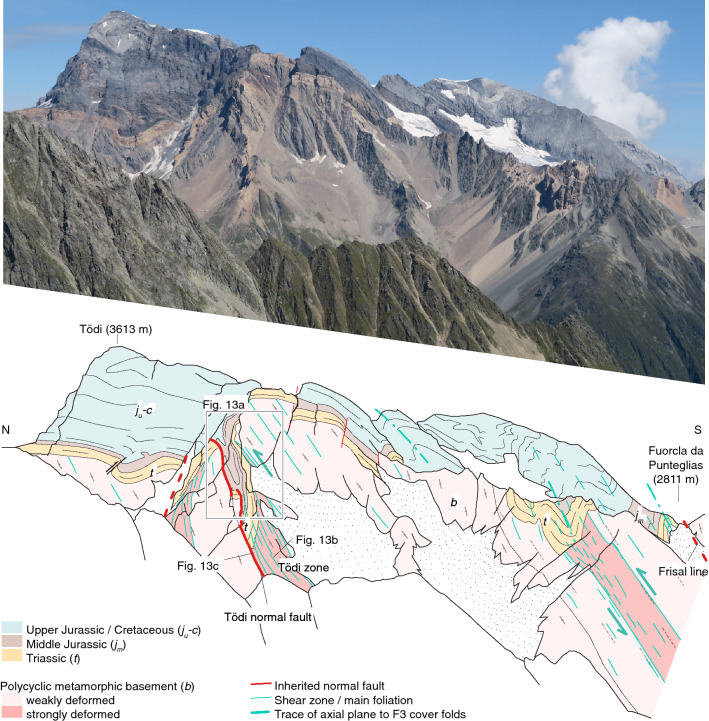
Profile-like view of the Tödi west face. The Tödi zone includes from north to south, a south-verging non-reactivated Jurassic? normal fault (Tödi normal fault), a pinched “syncline” including Mesozoic sediments, a strongly sheared sub-vertical to overturned stratigraphic contact to the crystalline (respectively Permo-Carboniferous) substratum, a decametre-wide ductile basement shear zone with S-up sense of shear. See text and Fig. 13a for a more detailed description. Location is given in Fig. 3a

**Fig. 13:**
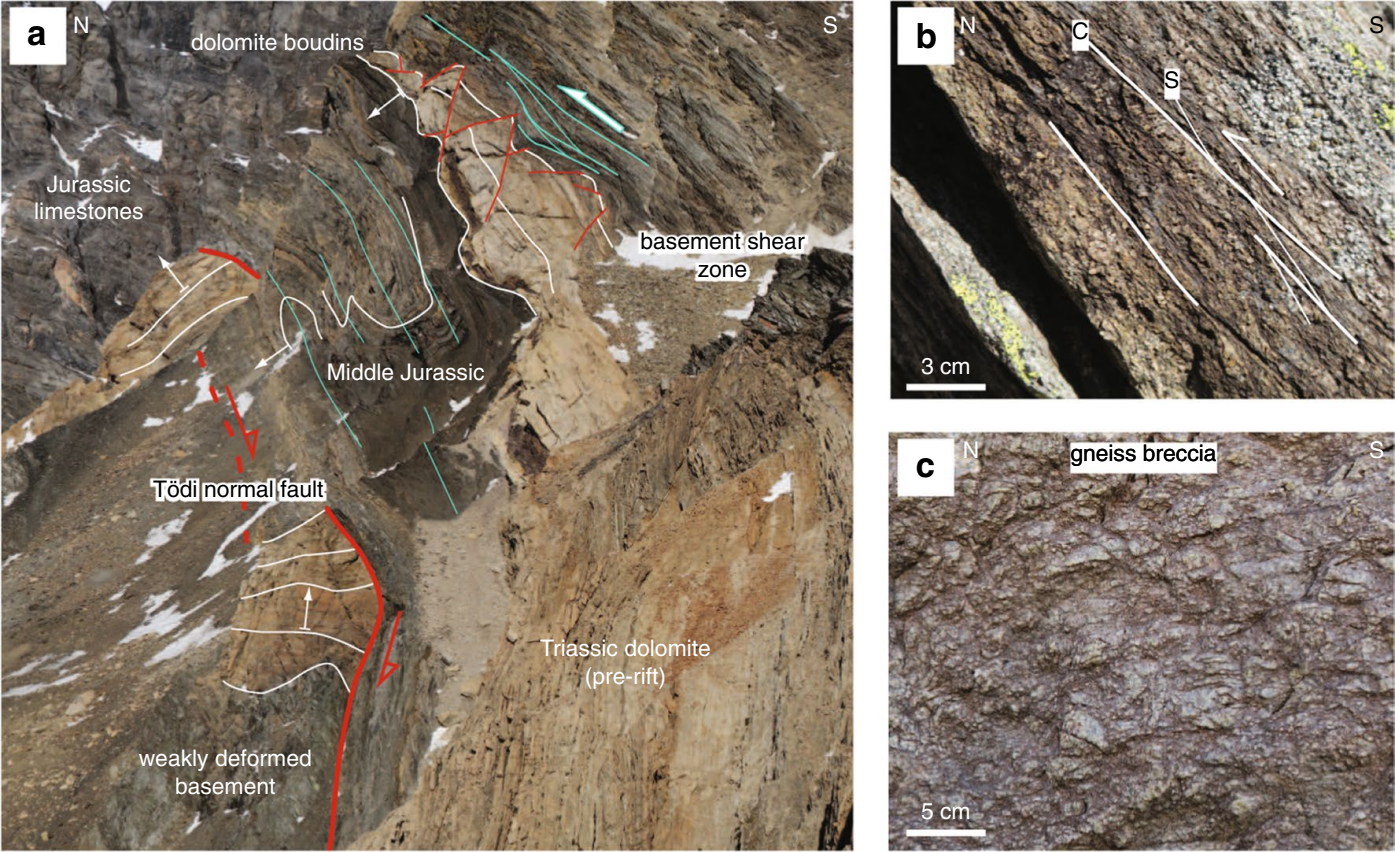
**a** Photograph of the Tödi zone. The tectonic contact in the north (Tödi normal fault, red line with arrow indicates Mesozoic extension). South of the synclinal area, a strongly foliated mylonitic basement shear zone with S-up kinematics (green arrow) can be traced into the vertical to overturned stratigraphic basement-cover contact. The approximate thickness of the Röti Formation (yellow dolomitic horizon) is 50 m. White arrows indicate the younging direction in Mesozoic strata. See Figs. 3a and 12 for location. **b** Mylonitic basement shear zone with S-up kinematics near the overturned stratigraphic basement cover contact (coordinates: $46.7953^{\circ }\text {N}/8.8974^{\circ }\text {E}$, see Fig. 12 for location). **c** Gneiss breccia near the Tödi normal fault. Clasts internally preserve pre-Alpine textures (coordinates: $46.7967^{\circ }\text {N}/8.8988^{\circ }\text {E}$, see Fig. 12 for location)

**Fig. 14:**
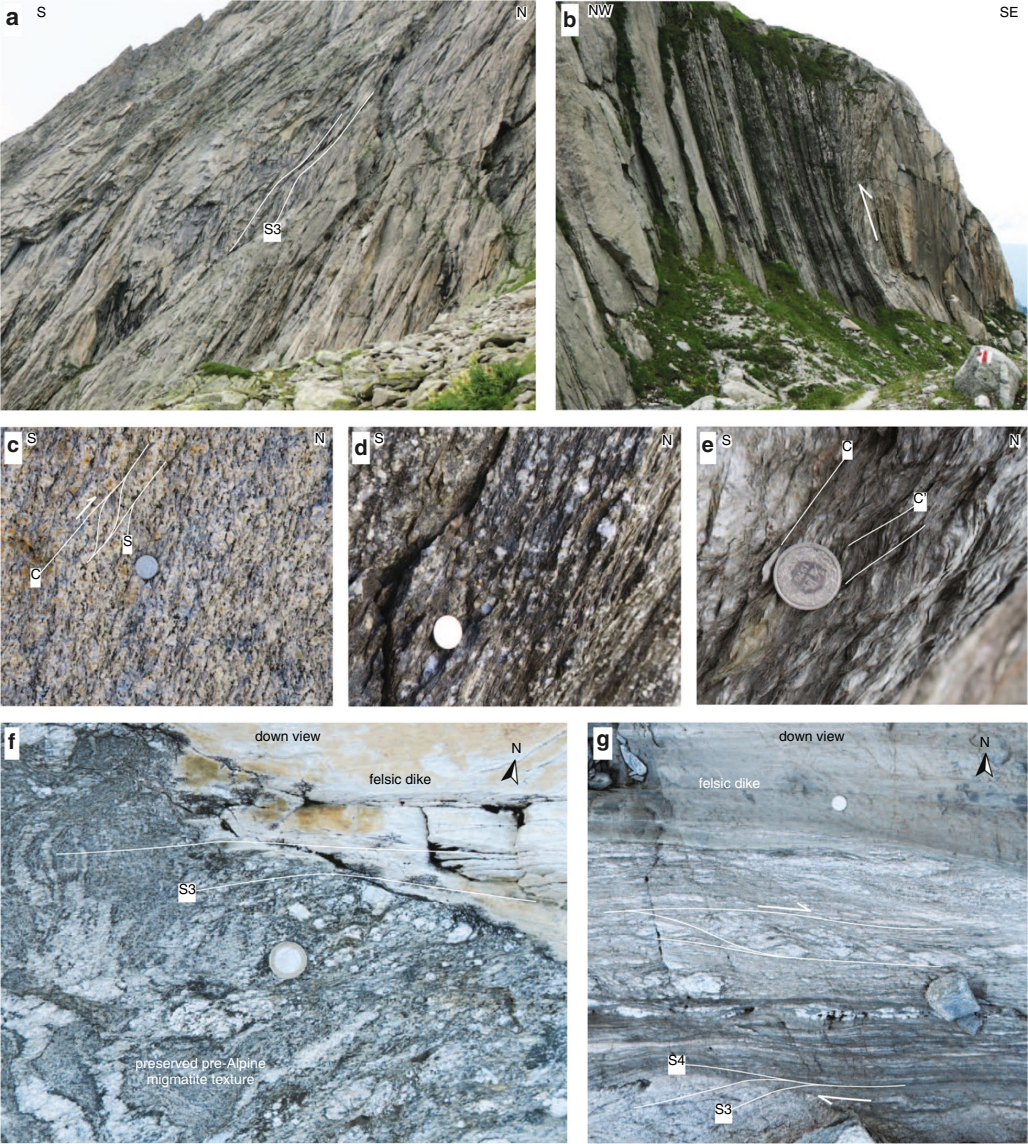
Shear zone patterns and kinematics in the basement. **a** Slightly undulating pattern of S3 at the decametre scale (coordinates: $46.6634^{\circ }\text {N}/8.4895^{\circ }\text {E}$, see Fig. 6 for location). **b** Major shear zone with S-block up kinematics (coordinates: $46.6396^{\circ }\text {N}/8.4580^{\circ }\text {E}$, see Fig. 6 for location). **c** Shearband cleavage (C/S fabric) in weakly deformed granite indicating S-block up kinematics (coordinates: $46.6213^{\circ }\text {N}/8.4708^{\circ }\text {E}$, see Fig. 6 for location). **d** Mylonite with feldspar porphyroclasts in a granite (coordinates: $46.6231^{\circ }\text {N}/8.4611^{\circ }\text {E}$, see Fig. 6 for location). **e** C’-type shear bands in the core of a phyllonitic shear zone indicating S-block up kinematics (coordinates: $46.6888^{\circ }\text {N}/8.5307^{\circ }\text {E}$, see Fig. 6 for location). **f** S3 intersecting the contact between a felsic dike and a migmatite with well-preserved pre-Alpine foliation (coordinates: $46.7118^{\circ }\text {N}/8.5174^{\circ }\text {E}$, see Fig. 6 for location). **g** D4 shear zone overprinting S3. Deflection of S3 indicates a dextral component of deformation. Stretching lineations associated with the outcrop indicate oblique dextral S-block up kinematics (coordinates: $46.7124^{\circ }\text {N}/8.5172^{\circ }\text {E}$, see Fig. 6 for location)

**Fig. 15:**
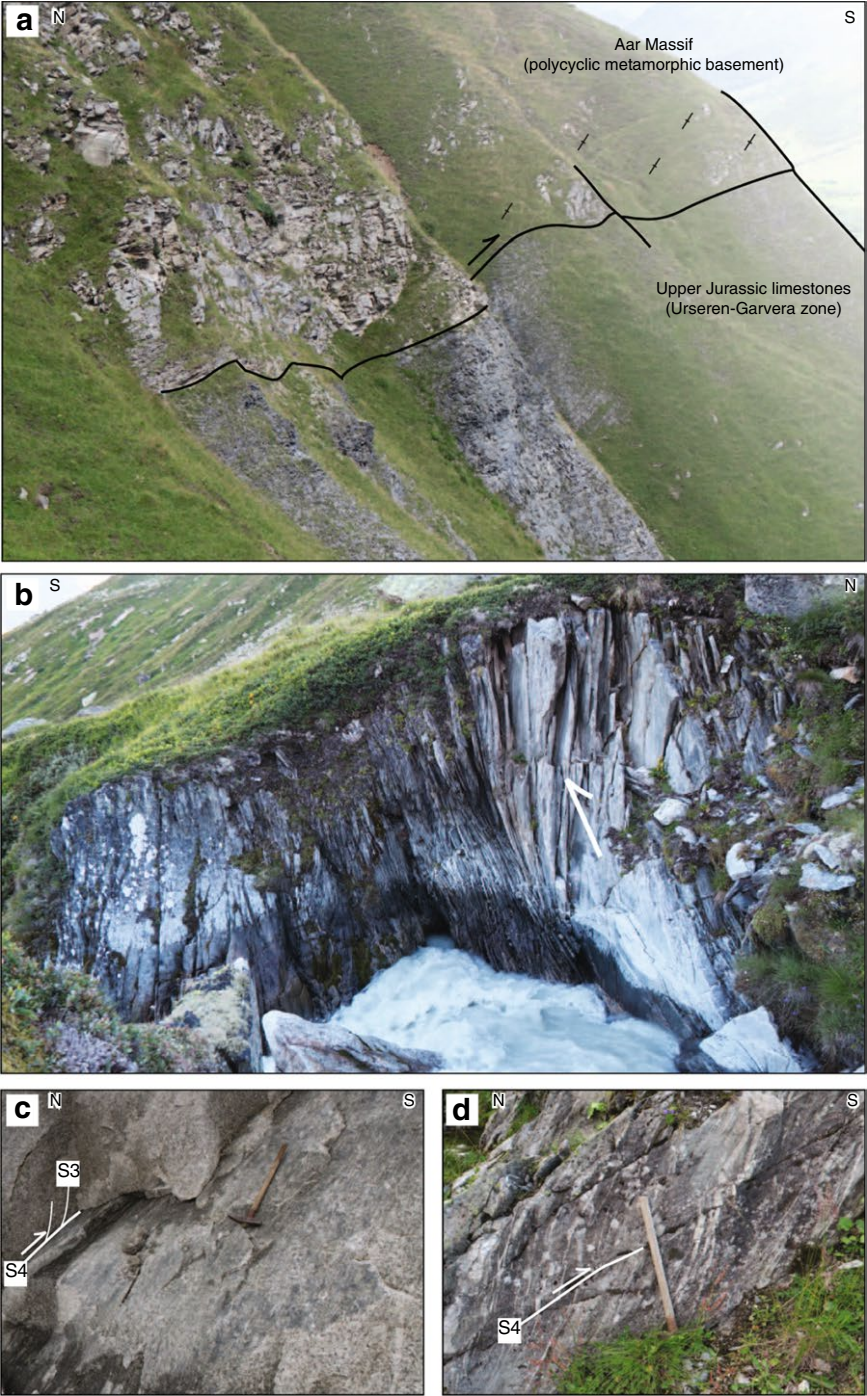
Deformation structures at the southern Aar Massif boundary **a** Top-to-the S shearing at the steeply NNW-dipping tectonic contact between the Aar Massif basement (left) and Jurassic limestones of the Urseren-Garvera zone (right) (coordinates: $46.6024^{\circ }\text {N}/8.4991^{\circ }\text {E}$, see Fig. 3c for location). **b** Major steeply NNW-dipping D3 shear zone near the southern Aar Massif boundary with N-block up kinematics (coordinates: $46.5972^{\circ }\text {N}/8.4581^{\circ }\text {E}$, see Fig. 3c for location). Height of the cliff $\sim 5\,\text {m}$. **c** Brittle-ductile D4 S-directed back-thrusts in the Central Aar granite (coordinates: $46.6200^{\circ }\text {N}/8.4866^{\circ }\text {E}$, see Fig. 7 for location). **d** Brittle-ductile D4 S-directed back-thrusts in gneiss (coordinates: $46.6075^{\circ }\text {N}/8.4924^{\circ }\text {E}$, see Fig. 7 for location)

South of the Windgällen-Färnigen zone (Fig. 6, subregion IIIA/B, IV), S3 dominates the structural appearance (Fig. 3). Although S3 is ubiquitous throughout the study area, a concentration of strain on up to decametre wide shear zones can be observed (Figs. 3,  9, 10, 11, 12, 13, 14 and 15). S3 strikes ESE-WNW (sub-parallel to the Aar Massif) and always steeper than the earlier Alpine foliations. Its dip increases from a mean value of $60^{\circ }$ SSE in the northern Aar Massif to a mean value of $80^{\circ }$ NNW in the southern Aar Massif, so that the resulting overall geometry in section view is an upright fan (Figs. 3c, 6 and 14a, b). S3 is associated with a down-dip stretching lineation (L3). In the basement, D3 forms a dense network of anastomosing shear zones (Fig. 2a) and sigmoidal bodies with lower strain (Fig. 14a–f). Indications for both NNW-block-up or SSE-block-up kinematics were observed (in agreement with Wehrens et al. 2017). However, D3 shear zones appear to be dominated by a SSE-block up/reverse sense of shear overall, which is confirmed by observed displacements along the basement-cover contact (Figs. 12, 13 and  14a–e). Close to the southern Aar Massif boundary (Fig. 6, subregion IV), we observed a shift to dominantly N-block up kinematics (Figs. 3 and 15a, b). In the limestone-dominated cover units, S3 represents the axial plane foliation to D3 folds at various scales and often forms a crenulation cleavage (Figs. 3, 9b and 10c, d). Axes to minor D3 folds plunge ENE with a mean angle of $5^{\circ }$ (Fig. 6). This indicates minor tilting in along-strike direction after D3.

D3 shear zones are characterised by an increased sheet silicate content (white mica, chlorite, biotite) and by a substantial reduction in grain size (see also, Challandes et al. 2008; Goncalves et al. 2012; Wehrens et al. 2017). Fig. 14c–e shows the often gradual evolution from a weakly deformed protomylonite (Fig. 14c) into a strongly foliated mylonite (Fig. 14d) and finally into an ultramylonite, which is strongly enriched in sheet silicates (phyllonite, Fig. 14e). South of the Windgällen-Färnigen zone, the presence of newly formed or recrystallised syn-kinematic biotite and quartz grains indicate that ductile shearing took place at biotite-stable greenschist facies, and thus near peak metamorphic conditions (Fig. 14d, e).

D3 shearing is often associated with the formation of mostly quartz-filled mode 1 extensional veins (Fig. 6, see also Steck 1968). Locally, syn-kinematic D3 veins are partly or completely transposed into the S3 plane during progressive shearing.

The Windgällen fold-and-thrust structure, which developed during D2 was refolded during D3 (Fig. 6, subregion IIA). Small displacements along S3, distributed in space, are presumably responsible for the development of the culmination of the northern Aar Massif, including the passive rotation and folding of the overlying Windgällen fold-and-thrust structure (Fig. 10a, see also Funk et al. 1983; Schenker 1980). Similarly, we attribute the northward tilting of early D2 thrusts at the northern massif-front (see Fig. 9e) to D3. As illustrated on Fig. 10a–e, the spacing of S3 decreases from north to south, which leads to the progressive steepening of earlier fabrics. Where the overturned lower limb of the Windgällen anticline passes into a basement shear zone, we observe a composite schistosity S0-S3 (Fig. 10e).

In the central Aar Massif, the main Alpine foliation and associated shear zones which formed during D3 are referred to as Handegg phase structures (Table 1, Herwegh et al. 2020; Mair et al. 2018; Wehrens et al. 2017). In the western Aar Massif, post-D2 deformation was summarised as Grindelwald phase (Burkhard 1988; Pfiffner 2015) and is considered the main phase of exhumation of the Aar Massif. In the Lower Helvetic east of the study area, the Calanda phase foliation is commonly overprinted by a steeper Ruchi phase crenulation cleavage (Milnes and Pfiffner 1977), which we here correlate with D3.

#### D4 structures

D4 is characterised by a transition to brittle-ductile shearing. D4 shear zones cross-cut or reactivate earlier foliations. Oblique to strike-slip faults and thrusts appear to be active synchronously within the study area, thus indicating significant strain partitioning during D4. The massif-internal regions (subregions IIIA/B in Fig. 3) were dominated by oblique to strike-slip faulting, while thrusting/back-thrusting dominated in the northern and southern Aar Massif, respectively (Fig. 7). The more discrete appearance of shear zones and the presence of syn-kinematic chlorite and white mica, as well as the absence of biotite, indicate retrograde metamorphic conditions during D4. In the central southern Aar Massif, biotite was still stable during early D4 strike-slip shearing (Rolland et al. 2009; Wehrens et al. 2017).

In the northern Aar Massif, D4 was dominated by discrete NNW-verging thrusts (Fig. 9d). These thrusts dip on average $150^{\circ }/25^{\circ }$ (Fig. 7). In the limestone-dominated cover units D4 forms tight to isoclinal NNW-verging folds with mostly flat ENE-trending fold axes. These thrusts are commonly associated with a second minor and possibly conjugate set of steep reverse faults, which show NNW-block up kinematics (mean dip-azimuth/dip = $330^{\circ }/75^{\circ }$). Associated stretching lineations or striae in the fault surface trend SSE and are consistently down-dip (Fig. 7). The two sets of faults intersect at a mean angle of $80^{\circ }$. In the north, D2 and D4 faults (Figs. 5 and 7) have similar characteristics (i.e., mean strike direction, trend of stretching lineation, kinematics, associated mineral phases, angle between possibly conjugate subsets). However, D4 faults crosscut the weakly developed foliation S3 (Fig. 9d) and intersect the basement-cover contact at a higher angle ($>50^{\circ }$, Fig. 3c) than D2 faults. This indicates that D4 faults formed during a late stage of deformation when top basement was already reorientated to its present-day dip. The most prominent D4 structure is an array of NNW-verging thrusts exposed in the northernmost Aar Massif, also known as Pfaffenchopf phase structures (Table 1, Herwegh et al. 2017, 2020; Labhart 1966; Mock 2014; Wehrens et al. 2017). Displacements along D4 thrusts range between millimetres up to 2 km (Pfaffenchopf thrust array, Herwegh et al. 2020).

Along the Windgällen-Färnigen zone and in the northern Tödi region (Fig. 7, subregions IIA/B, IIIA), the orientation and characteristics of D4 faults remain relatively uniform. However, both the abundance and the estimated displacements along the faults decrease from north to south (Fig. 11a). Figure 10e shows an example of a minor discrete brittle-ductile D4 shear zone crosscutting the composite S1-S3 schistosity of the Windgällen-Färnigen zone. Associated displacements, when recognisable, are mostly below 10 m.

In the massif-internal regions and in the southern Aar Massif (Fig. 7, subregions IIIA/B, IV) D4 was characterised by a transition to oblique to strike-slip dominated ductile to brittle-ductile shearing (see also Oberaar phase structures in Wehrens et al. 2017, Table 1). Sub-vertical and mostly E-W to SE-NW striking, oblique S-block up to dextral shear zones are most abundant. These faults commonly reactivate S3 or crosscut earlier fabrics (Fig. 14g). A likely conjugate minor subset of sub-vertical on average N-S striking shear zones with an oblique E-up to sinistral sense of shear was also observed (Fig. 7), which is in agreement with Rolland et al. (2009). D4 strike-slip shearing is often associated with sub-vertical NNW-SSE striking mode 1 extensional quartz veins (Fig. 7), which are, in the central Aar Massif, clearly younger than D3 veins (Bergemann et al. 2017; Ricchi et al. 2019). The abundance of strike-slip dominated faults decreases from west to east. Although difficult to estimate, associated displacements are estimated to be $<100$ m. This is in contrast to the central and western Aar Massif, where Steck (1984), Rolland et al. (2009) and Wehrens et al. (2017) have documented significant strike-slip movements, especially in the southern domains.

However, in the southern Aar Massif of our study area (Fig. 7, subregion IV), SSE-verging thrusts (Fig. 15c, d) and a minor likely conjugate set of NNW-verging thrusts dominate, and are attributed to D4. Based on their opposite vergence with respect to the overall north-northwest directed transport direction in the Helvetics (Pfiffner 2015), the SSE-verging thrusts are in the following referred to as back-thrusts. Back-thrusts dip $50^{\circ }$ towards the NNW, whereas the NNW-verging faults dip $45^{\circ }$ towards the SSE. Associated stretching lineations are down-dip. Both the frequency of back-thrusts and associated top-to-the SSE displacements increase towards the Urseren-Garvera zone and are mostly in the order of centimetres to metres.

#### D5 structures

A number of late brittle faults have been observed in the field. Their young age is mainly inferred from crosscutting relationships. Where these reactivate pre-existing foliations, which is often the case especially in the basement, their young age is inferred from the presence of non-cohesive fault gouge material in the fault core. In previously strongly foliated limestone-dominated Mesozoic units, D5 faults were more easily identified. Associated displacements, when observable, are mostly below 10 m.

D5 faults are steep to sub-vertical and mostly dominated by strike-slip kinematics, sometimes with a significant normal fault component. We did not find evidence for compressional deformation. The orientations of four subsets of faults, classified after dextral, sinistral, normal fault or N-block-up kinematics are shown on Fig. 8. Dextral faults are most abundant and show significant variation in strike direction ranging from $50^{\circ }$ to $140^{\circ }$. Sinistral faults strike from 140 to $210^{\circ }$ with a mean value of $180^{\circ }$. Late strike-slip faults often reactivate pre-existing D3 and D4 faults, which is in general agreement with Egli et al. (2018), Belgrano et al. (2016) and Hofmann et al. (2004). The normal faults can be subdivided into two subsets, based on the fault orientations and the associated extension direction. The first set of normal faults, which was mainly observed in the northern Aar Massif (Fig. 8, subregions I and IIA/B), comprises a possibly conjugate set of NNW-SSE striking normal faults. These normal faults have dip values varying between 60 and $75^{\circ }$. The second subset comprises a limited number of normal faults, which show large variations in strike but are always associated with orogen-perpendicular extension. Comparable observations were made in the Vättis region (Pfiffner 1972). Very few observations of E–W striking sub-vertical faults with N-block up kinematics were made. These are correlated with the “Gadmen faults”, as known from the northern central Aar Massif (Table 1, Berger et al. 2017b; Herwegh et al. 2020; Mock 2014; Labhart 1966).

#### Alpine shear zones and their relationship to inherited normal faults

We recognised three Alpine shear zones in which inherited passive margin structures (rift-basins and their bounding normal faults) played a key role during the Alpine inversion. In the following, we describe their characteristics and influence on Alpine collisional deformation.

*Engelbergertal* Two minor inherited normal faults are preserved in the footwall to a D2 fault in the northernmost Aar Massif (Fig. 9a; see map in Fig. 5 for locality). Normal fault displacements are on the order of 2–4 m. The basal Triassic sandstone and the underlying crystalline basement are displaced, thus suggesting a Triassic or younger age for the normal faults. Both normal faults are associated with S-block down movements. The normal faults cut the basement-cover contact at an angle of approximately $60^{\circ }$. During early Alpine compressional deformation (D2), an array of thrusts formed in the footwall to these normal faults. These thrusts intersect the normal faults at a high angle ($70^{\circ }-80^{\circ }$). The angular relationship between basement-cover contact, inherited normal faults and D2 thrusts indicates that these thrusts developed at a time when top basement was still S-dipping (Fig. 9e, see also Nibourel et al. 2018). Assuming an initial fault dip of $25^{\circ }-30^{\circ }$ (this value corresponds to the dip of late D4 thrusts), a south-southeasterly dip of $10^{\circ }-15^{\circ }$ can be estimated for top basement for the time of the formation of D2 faults (see schematic sketch on Fig. 9e).

*Windgällen-Färnigen zone* At Färnigen (see Fig. 3c for locality) the Windgällen-Färnigen zone comprises two synformal sediment wedges. These are in the following referred to as the northern and the southern sediment wedge, separated by a triangle-shaped antiformal basement block. The overall structure has a mean easterly axial plunge of $6^{\circ }$ and appears to be strongly non-cylindrical. Figure 11a shows a section-like view of the Färnigen zone.

The northern sediment wedge is bound by the Färnigen normal fault, a major SSE-down normal fault with an estimated throw of up to 1 km (Figs. 3c and 11a, see also Kammer 1985). The Färnigen normal fault is marked by an up to metre thick non-cohesive fault breccia (Fig. 11b). The directly adjacent gneisses are dissected by cataclasites, which indicate S-block down normal fault kinematics. A few metres away from the normal fault, gneisses preserve their pre-Variscan foliation and are largely unaffected by Alpine deformation (Fig. 11b). The normal fault strikes 060$^{\circ }$ ENE and dips $70^{\circ }-80^{\circ }$ SSE. Stretching lineations on the fault surface are down-dip (Fig. 11b). The Färnigen normal fault was weakly reactivated as a steep reverse fault during Alpine inversion, as indicated by an increasingly sharp angle between the Mesozoic strata and the fault plane with decreasing distance to the normal fault (Fig. 11a). This is consistent with the presence of a D3 basement shear zone, which developed in the hanging wall to the normal fault (Fig. 11a). The southern “limb” of the northern wedge is characterised by a $45^{\circ }$ NNW dipping stratigraphic contact between Middle Jurassic strata and their crystalline substratum. The northern wedge comprises weakly deformed Middle Jurassic to Cenozoic sediments in stratigraphic upright position (Kammer 1985). The former half-graben geometry is still recognisable. A pervasive upright main foliation including a vertical mineral stretching lineation, which we mainly attribute to D3, indicates near coaxial shortening in the northern wedge. This is in contrast to dominantly NNW-up kinematics in the underlying $65^{\circ }$ SSE dipping basement shear zones and indicates decoupled deformation in the basement and cover units, also in the absence of major detachments.

A second SSE-vergent inherited normal fault appears to be located in the north of the southern wedge. This second normal fault is poorly exposed but can be inferred from (i) the presence of Permo-Carboniferous clastic and volcanoclastic sediments only in its hanging wall, (ii) the elimination of the Middle Jurassic strata in its footwall (Kammer 1985) and (iii) cataclasites in the adjacent crystalline basement (Kammer 1985). It dips approximately $70^{\circ }$ towards SSE. The sediments of the southern wedge represent the fill of a second half-graben. They were strongly sheared and folded, mainly during D2, then refolded during D3. In the relatively homogeneous Upper Jurassic to Cretaceous limestones, S2 and S3 (and minor S1) form a composite foliation, which dips to the SSE with a value decreasing with elevation from $90^{\circ }$ at the bottom of the wedge to $35^{\circ }$ in the upper wedge region. This downward steepening appears to be due to a decreased spacing of the steep to sub-vertical S3 (Fig. 11c). Three decametre-scaled tight to isoclinal synclines can be identified at the bottom of the wedge and are connected to the southern limb with an overturned stratigraphic contact to the crystalline substratum (Fig. 11e). Ductile shearing was most intense between the synclinal area and the overturned southern limb, as recorded by deformed ooides in Middle Jurassic strata (Kammer 1985). The strongly sheared overturned limb can be traced into a decametre-wide mylonitic to phyllonitic basement shear zone, which on average dips $65^{\circ }$ SSE (Fig. 13a).

The presence of Permo-Carboniferous sediments in the southern sediment wedge (Fig. 11e) indicates some pre-Triassic normal faulting. Thickness variations in the Middle Jurassic, including the deposition of sandstones and polymict conglomerates containing Triassic dolomite and crystalline basement clasts (Fig. 11e) point to a high topographic relief during the Middle Jurassic, which we attribute to relative uplift of the footwall block during normal faulting. A $\sim 10\,\text {cm}$ thick Nummulite-bearing Cenozoic calcareous sandstone at the contact to the Färnigen normal fault (Fig. 11b) indicates that normal faulting possibly persisted into the Cenozoic, or that it was reactivated due to the loading of the advancing Alpine orogenic wedge. Alternatively, the discordant contact between Cenozoic strata and the crystalline basement could also be interpreted as a paleo-escarpment. In both cases, significant topographic relief is required to explain the contact situation shown on Fig. 11b, which points to pulses of normal faulting during the Permo-Carboniferous, Lower Mesozoic and eventually Creataceous-Cenozoic (cf. Cardello and Mancktelow 2014). Assuming the normal fault formed at a typical $60^{\circ }$ dip angle, the present day orientation of the fault plane implies a clockwise back-rotation (in section view, looking east) of about $\sim 10^{\circ }$ during Alpine deformation (assuming a $10^{\circ }$ southerly dip of top basement at initiation of D3 shearing, Nibourel et al. 2018).

*Tödi zone* The Tödi zone (see Fig. 3a for locality) comprises, from north to south, a major inherited normal fault (Tödi normal fault), a narrow syncline (Tödi “syncline”) including Mesozoic strata, a southern limb with overturned stratigraphy, a several tens of metres wide steeply SSE-dipping basement shear zone and finally a broad antiformal culmination in the basement (Figs. 12 and 13). The SSE-vergent Tödi normal fault is a major structure, which was not reactivated during Alpine collisional deformation (Fig. 13a). It can be subdivided into two segments, separated by an up to 50 m wide fault block Fig. 13a). Each of these segments accommodated approximately 250 m of displacement during normal faulting, which amount to an estimated SSE-down displacement of 500 m (when dip-slip kinematics are assumed, see Fig. 13a). Triassic pre-rift strata are still attached to the footwall, the intermediate fault block and to the hanging wall to the normal fault, while they were eliminated along the fault surface (Figs. 12 and 13a). This is consistent with the larger thickness of the Middle Jurassic strata in the Tödi “syncline” (i.e., the inverted half-graben, Fig. 13a), thus attesting to active rifting during the Middle Jurassic (cf. Günzler-Seiffert 1941). Note that thickness-variations could partly be due to layer thickening during folding (Ramsay and Huber 1987). The Tödi normal fault intersects the basal Mesozoic strata at an angle of approximately $60^{\circ }$ (Fig. 13a). Basement and cover units adjacent to the northern tectonic normal fault contact were not significantly affected by Alpine deformation as highlighted by (i) preserved pre-Alpine fabrics in the basement or (ii) by primary sedimentary structures in the cover strata. In the basement close to the normal fault, we observed monomictic breccias with internally weakly deformed clasts, which are presumably related to the normal faulting (Fig. 13a).

South of the synclinal area, the spacing of S3 decreases rapidly to form a mylonite/ultramylonite with a well-developed SSE trending down-dip stretching lineation (L3) (Figs. 6 and 13b). This basement shear zone can be traced into the overturned southern limb of the Tödi “syncline”. Although both basement and cover units are strongly sheared, the stratigraphic contact between the mylonitic basement and the basal Triassic strata is intact (Fig. 13a). Along the overturned limb, the originally up to 50 m thick Triassic dolomite (Röti Formation) is thinned and boudinaged, indicating significant layer parallel and thus almost vertical extension during D3 shearing. The phyllonitic foliation of the D3 basement shear zone locally bends around the irregularly-shaped contact between dolomite boudins and basement. Hence, during D3 deformation, dolomite was more competent than both Jurassic marls and limestones and sheet-silicate dominated mylonites, which developed in the basement. The spacing of S3 increases again as we move towards the adjacent basement-cored anticline. In the Middle Jurassic marls of the Tödi syncline, S3 forms a tightly spaced crenulation cleavage, which strikes $065^{\circ }\text {ENE}$ and dips $55^{\circ }$ to $75^{\circ }\text {SSE}$. We were not able to detect a measurable difference between the mean dip of S3 in the cover sediments of the Tödi syncline and the adjacent basement shear zone. Similarly, no systematic variation in dip was recorded between S3 in strongly localised basement shear zones and adjacent weakly deformed basement. The dip of S3 systematically decreases towards the topographically higher Upper Jurassic limestones. This is interpreted to reflect ongoing N-directed movements along the roof-thrusts such as the Cavestrau décollement or the basal Helvetic thrust during D3 (Fig. 3a).

Both, the geometry of top basement and outcrop scale kinematic indicators, such as shear bands and schistosity forming sigmoidal bodies (Fig. 3b), indicate reverse S-block up kinematics along the Tödi zone during D3. The asymmetric distribution of D3 shearing, which mainly affects the southern overturned limb of the “syncline” highlights that the Tödi “syncline” is not a true syncline but rather an inverted half-graben, where only the hanging wall to the Tödi normal fault was affected by steep NNW-directed reverse shearing, while the inherited normal fault acted as a buttress (see also Bellahsen et al. 2012; Tricart and Lemoine 1986). Geometrical considerations (i.e. the acute angle of D3 shear zones with respect to inherited normal faults, which intersect top basement at $60^{\circ }$, Fig. 6) demonstrate that D3 reverse faults had a dip of at least $50^{\circ }$ at inception, or even more if we consider that top basement was dipping to the S with $10^{\circ }-15^{\circ }$ at peak temperature conditions (Nibourel et al. 2018).

## RSCM results and interpretation

**Fig. 16:**
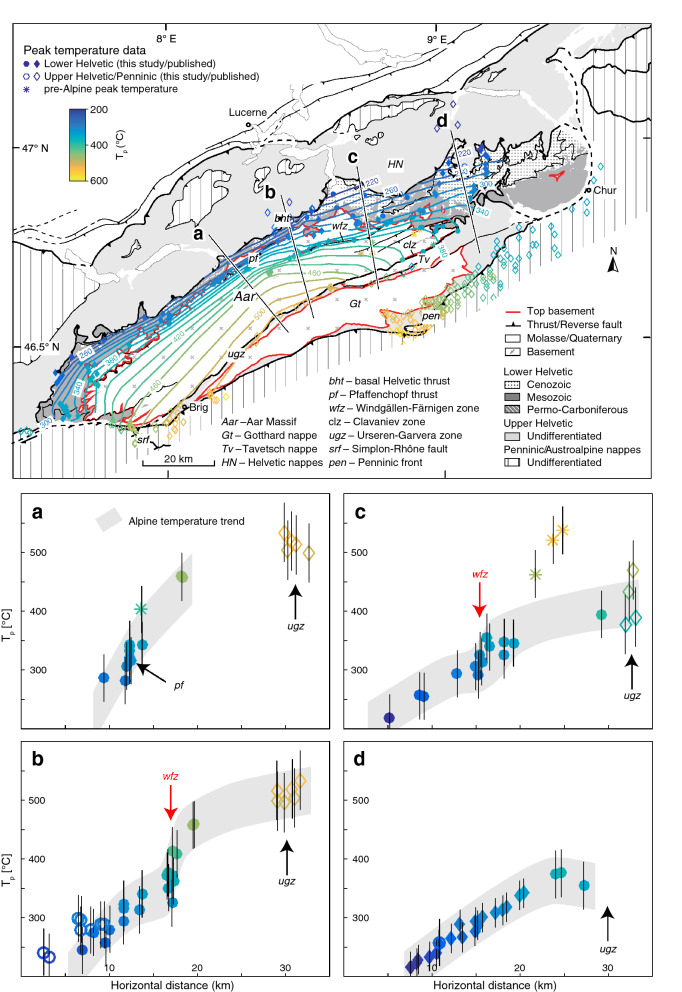
Simplified structural map (modified from Schmid et al. 2004) including new and published peak temperature estimates based on RSCM (Beyssac et al. 2002; Erne 2014; Girault et al. 2020; Hafner 2016; Lahfid et al. 2010; Mair et al. 2018; Negro et al. 2013; Nibourel et al. 2018; Wiederkehr et al. 2008) and calcite-dolomite thermometry data (Herwegh and Pfiffner 2005). Inferred isograds representing peak Alpine metamorphic conditions are shown for the Lower Helvetic domain, normalised to the 1500 m elevation level, assuming a constant geothermal gradient of $25\,^{\circ }\text{C/km}$ (see Nibourel et al. 2018, for a discussion). **a**–**d** New and published elevation-normalised (1500 m elevation level) $T_p$ plotted along four massif-perpendicular transects of unit length. Maximum projection distance = 10 km; Projection angle (trend): $058^{\circ }$ (A), $070^{\circ }$ (**b**–**d**). The approximate positions of major detachments such as the Urseren-Garvera zone, the Windgällen-Färnigen zone and the Pfaffenchopf thrust are highlighted with black and red arrows. Profile edge coordinates are (lat/lon): **a** $46.7779^{\circ }\text {N}/8.1588^{\circ }\text {E}$, $46.5328^{\circ }\text {N}/8.4423^{\circ }\text {E}$ **b** $46.8748^{\circ }\text {N}/8.4224^{\circ }\text {E}$, $46.5680^{\circ }\text {N}/8.5342^{\circ }\text {E}$ **c** $46.9441^{\circ }\text {N}/8.6996^{\circ }\text {E}$, $46.6374^{\circ }\text {N}/8.7708^{\circ }\text {E}$ **d** $47.0298^{\circ }\text {N}/9.0437^{\circ }\text {E}$, $46.7225^{\circ }\text {N}/9.1392^{\circ }\text {E}$

**Fig. 17:**
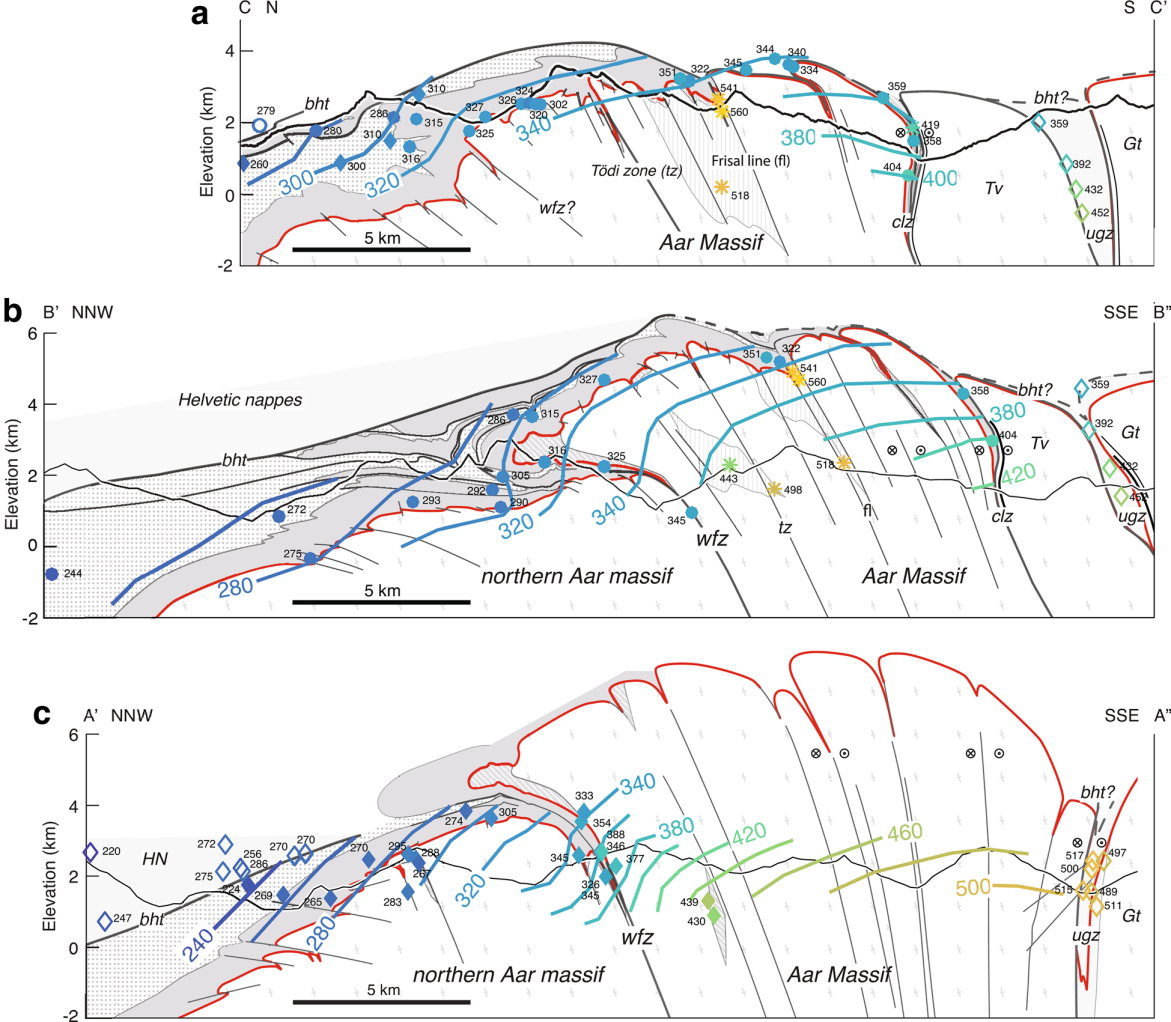
RSCM temperature data projected into three stacked cross sections in descending order from east to west (maximum projection distance RSCM data = 10 km). Inferred isograds highlight the metamorphic pattern in section view. Scale is equal on all cross sections. Section traces are shown on Fig. 1a. Legend and abbreviations as on Fig. 16. References, projection direction and edge coordinates as on Fig. 3. **a** Section A′–A′′. **b** Section B′–B′′. **c** Section C–C′

RSCM results are listed in Additional file 1: Table S1 with key parameters such as location, stratigraphic age, the number of sufficiently good fitted spectra and $T_p$ with associated uncertainties. The distribution of obtained $T_p$ single measurements in each sample is shown in Figures S1-S4 of Additional file 1. RSCM records peak temperature but does not carry information about the time when it was reached. Hence, the $T_p$ values obtained do not necessarily represent Alpine metamorphism. RSCM data shown as dots and diamonds in Figs. 16 and 17 are considered to represent Alpine peak metamorphism. A few samples appear to yield pre-Alpine temperatures or were identified as clear outliers. These samples are highlighted as asterisks on Figs. 16 and 17, and are discussed below.

### Pre-Alpine temperatures and outliers

In the study area, Mesozoic or younger sediments are likely to yield Alpine peak temperatures. In contrast, pre-Triassic sediments could be affected by a pre-Alpine (Variscan) metamorphic event (Eugster 1951; Franks 1968a, b; Labhart 1977; Oberhänsli et al. 1988; Schaltegger et al. 2003; Schenker 1987; Schenker and Abrecht 1987). In the absence of Mesozoic or younger strata, many of the massif-internal samples were taken from pre-Triassic sediments. In these sediments, the age of metamorphism has to be assessed carefully. Based on their age and Variscan thermal and structural overprint, these deposits are subdivided into three subgroups. A first group (Cavardiras Group, Berger et al. 2017a) summarises Pre- to Early Variscan deposits, which were affected by Variscan amphibolite hornfels facies contact metamorphism and subsequent greenschist facies metamorphism (Böhm 1986; Franks 1968a, b; Oberhänsli et al. 1988). Given their additional Alpine overprint, these deposits can be considered polymetamorphic. A second group summarises late to post-Variscan deposits. The rocks of the second group may, in the vicinity of intrusion contacts, be affected by contact metamorphism (as demonstrated for example for the Diechtergletscher formation, Schenker and Abrecht 1987). The deposits of groups one and two have locally been tilted, wedged into their crystalline substratum or buried to depths of 5 to 10 km during what is referred to as “Permian tectonics” (Franks 1968b; Gisler 2018; Oberhänsli et al. 1988). Finally, a third subgroup (Glarus Verrucano Group) includes the youngest Permian deposits. These deposits are conformably overlain by the Mesozoic sediments and only affected by Alpine metamorphism (e.g., Berger et al. 2017a).

Pre-Alpine temperatures 517–560 $\,^{\circ }\text{C}$ were recorded in the pre-Visean Val Gliems Formation (Cavardiras Group, samples GL-16-02, GL-16-07 and EZ-15-02; Figures S3 and S4, Additional file 1). The temperatures recorded in these samples are in accordance with the presence of amphibolite facies minerals and can be attributed to Variscan amphibolite facies metamorphism (Böhm 1986; Eugster 1951; Franks 1968b).

Samples EZ-15-05 and 009 from the Permo-Carboniferous Tscharren Formation yielded relatively high peak temperatures of $497\,^{\circ }\text{C}$ and $443\,^{\circ }\text{C}$, respectively (Figure S3, Additional file 1). More than 22 spectra were obtained on each of the samples. Associated temperature distributions are relatively narrow with mean values well above what would be expected from the Alpine metamorphic pattern as highlighted by neighbouring Mesozoic samples. Both sample localities are located close to the intrusive contact to the late-Variscan central Aar Granite. Therefore, these elevated temperatures are interpreted to reflect late-Variscan contact metamorphism. Given the narrow temperature distribution in both samples, input of detrital high-temperature graphite is unlikely.

Sample BL03 from the Permo-Carboniferous Wendenjoch Formation of the Haslital region shows a very broad and complex temperature distribution. A clear peak is shown at $385\,^{\circ }\text{C}$ by 20 out of 30 measurements (see histogram on Figure S1, Additional file 1). More than 9 measurements indicate peak temperatures between 400 and $500\,^{\circ }\text{C}$, which points to the presence of detrital high-grade graphite in this sample. The main peak at $385\,^{\circ }\text{C}$, however, appears to be in accordance with the overall Alpine metamorphic pattern and is therefore considered to reflect Alpine metamorphism. Other samples from the Wendenjoch Formation also yielded Alpine peak temperatures (see below).

Other Permo-Carboniferous samples are interpreted to yield Alpine peak temperatures, as indicated by their good alignment with the overall Alpine metamorphic pattern and with published Alpine metamorphic mineral assemblages (e.g., Frey and Ferreiro Mählmann 1999). This is also confirmed by several paired Mesozoic/Permo-Carboniferous samples at the localities Wendenjoch (samples 002/WE-16-02-2), Färnigen (samples FA-15-21/FA-15-22b), Sandpass (RU-15-02/RU-15-03) and Bifertengrätli (samples TO-15-02/TO-15-03), which yielded almost equivalent and thus Alpine peak temperatures with very minor input of detrital high-grade graphite (Figures S1-S4, Additional file 1). However, it becomes difficult to determine the age of peak temperatures in samples which were affected by two or more almost similar metamorphic events.

Samples TR-15-01A and TR-15-01B (two sub-samples from the same sampling locality), both presumably of Middle Jurassic age, yielded relatively narrow temperature distributions around $420\,^{\circ }\text{C}$ (Figure S4, Additional file 1). These values are more than $50\,^{\circ }\text{C}$ higher than $T_p$ obtained on neighbouring samples that are located at a distance of less than 5 km along-strike. This difference is well above the precision indicated by the temperature distributions shown on Additional file 1: Figure S4. Therefore, these data points were classified outliers. Such abnormally high temperatures could be due to (i) frictional heating along the major detachment of the Clavaniev zone or (ii) increased structural order of the carbonaceous material due to aseismic shear (e.g., Kedar et al. 2020).

The pre-Alpine $T_p$ values and clear outliers discussed above were not considered for the subsequent analysis of the Alpine metamorphic pattern.

### Method used for contouring RSCM data

The good spatial resolution of RSCM data allows exhumed $T_p$ patterns to be analysed in three dimensions, as highlighted by paleo-isotemperature contour lines (in the following referred to as “isograds”, Figs. 16 and 17). Isograds in map view were calculated for the 1500 m elevation level (mean sample elevation) based on elevation-corrected Alpine peak temperature data, assuming a constant $25\,^{\circ }\text{C/km}$ geothermal gradient (Nibourel et al. 2018). This correction is justified by the observed distance between isograds in section view (Fig. 17). Although such a correction may introduce some bias, especially where exhumed paleo-isothermal surfaces are steep or deformed, it is necessary to normalise $T_p$ data, which are distributed over more than 3500 m topographic relief. Isograds were calculated using a linear interpolation algorithm (marching squares method, Matlab R2015b) at a 2 × 2 km grid size. Isograds were then manually adjusted for clear outliers.

Isograds were only drawn for the footwall to the basal Helvetic thrust, where Alpine peak metamorphism was reached at $\sim$ 22–17 Ma with presumably minor diachrony (Nibourel et al. 2018). Although the age record is not complete, published geochronological constraints (Berger et al. 2017b; Challandes et al. 2008; Glotzbach et al. 2010; Nibourel 2019; Rolland et al. 2008; Vernon et al. 2009; Wangenheim 2016) do not show clear evidence for significant diachrony in the age of Alpine peak metamorphism in the footwall to the basal Helvetic thrust. Therefore, the isograds shown on Fig. 16 and 17 may give indications about the thermal structure in the Lower Helvetic domain at peak metamorphism conditions. In contrast, temperature data from above the basal Helvetic thrust, representing a discontinuity with respect to the age and grade of Alpine metamorphism, were not considered for the construction of isograds. On Figs. 16 and 17, samples from the Helvetic nappes and higher tectonic units are highlighted with different symbols. In the central southern and western Aar Massif, larger data gaps exist due to the absence of suitable sediment exposures for RSCM analysis. There, isograds were interpolated over $>20\,\text {km}$ and are thus associated with significant uncertainties.

**Fig. 18:**
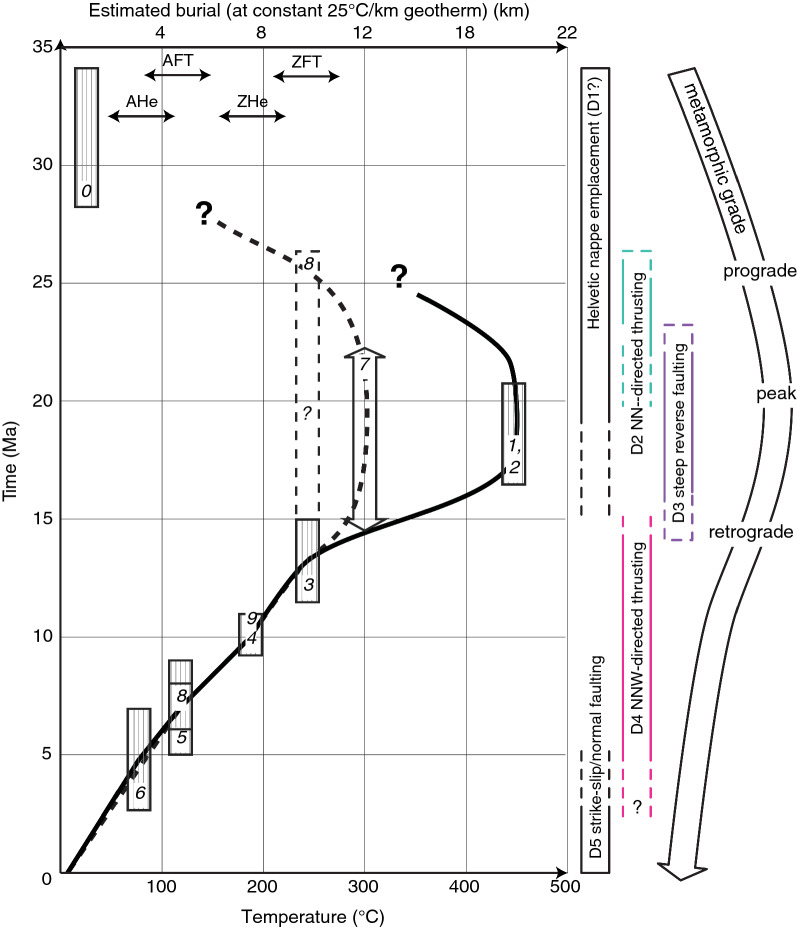
Temperature-time(-burial) paths for two key localities in the northern (see red asterisk on tectonic sketches, Fig. 19) and southern (white asterisk, Fig. 19) Aar Massif, respectively. The proposed timing of deformation phases D1–D5 is highlighted for reference. Temperature-time constraints for the southern Aar Massif are: 0—stratigraphic age Matt Formation (Menkveld-Gfeller et al. 2016), 1—Ar/Ar on biotite (Rolland et al. 2009), 2—Ar/Ar on biotite, Rb/Sr on phengite (Challandes et al. 2008), 3—zircon fission track ages (Glotzbach et al. 2010; Michalski and Soom 1990), 4—(U-Th)/He on zircon (Nibourel 2019), 5—apatite fission track ages (Michalski and Soom 1990; Nibourel 2019), 6—(U-Th-Sm)/He on apatite (Glotzbach et al. 2010); Temperature-time constraints for the northern Aar Massif are: 7—RSCM $T_p$ (Nibourel et al. 2018), 8—zircon fission track ages (Herwegh et al. 2020; Nibourel 2019; Wangenheim 2016), 9—(U-Th)/He on zircon (Nibourel 2019), 10—apatite fission track ages (Michalski and Soom 1990; Nibourel 2019)

Tectonic reconstructions by Wyss (1986) suggest that the sediments of the Urseren-Garvera zone represent the portion of the Gotthard nappe cover which was not transported to the north with the Helvetic nappes, but remained attached to its crystalline substratum. Such an interpretation is also supported by U-Th-Pb ages on peak metamorphic allanite (17–21 Ma, Janots and Rubatto 2014), which are almost equivalent to the inferred age of peak metamorphism in the adjacent Aar Massif basement (see also Fig. 18; Nibourel et al. 2018). During the emplacement of the Gotthard nappe, the main detachment likely jumped from the Gotthard nappe cover to the base of the Gotthard nappe, so that parts of the shortening accommodated along the basal Helvetic thrust root in the Urseren-Garvera zone. However, based on the above constraints, we assign the Gotthard nappe cover of the Urseren-Garvera zone (clearly Upper Helvetic from a stratigraphic point of view) to the footwall of the basal Helvetic thrust. As a consequence, $T_p$ data from the Urseren-Garvera zone are taken into account for the construction of isograds and to image the approximate thermal structure of the Lower Helvetic at peak metamorphic conditions (Figs. 16 and 17).

### Alpine metamorphic pattern in map view

New and published peak temperature data are presented in map view (Fig. 16, references are given in the Figure caption). In the footwall of the basal Helvetic thrust, we observe a general increase of temperature from 240–280$\,^{\circ }\text{C}$ along the northern massif front to up to $\sim 520\,^{\circ }\text{C}$ in the Urseren-Garvera zone. Along the northern massif front, isograds trend ENE and are parallel to the main strike of the massif. To the south, isograds become progressively oblique and rotate into a NE trend in the western Aar Massif, respectively into an E trend in the eastern Aar Massif. At the western and eastern edge of the massif, we locally observe almost orogen-perpendicular isograds, so that the overall pattern reflects the domal shape of the Aar massif. Isograds are most tightly spaced at the northern central massif-front and along the Windgällen-Färnigen zone. Towards the south, the isograd spacing increases non-linearly. Towards the east and west corners of the Aar Massif, the spacing between isograds generally increases and becomes more regular.

The exposed horizontal Alpine temperature gradient along four massif-perpendicular transects decreases from $>12\,^{\circ }\text{C/km}$ along transect A (central Aar Massif) to a value $<6\,^{\circ }\text{C/km}$ along transect D in the Glarus area (Fig. 16a–d). The horizontal Alpine temperature trend is non-linear, especially in the central Aar Massif (transects A and B), and becomes progressively sub-linear towards the east (Fig. 16c, d). The steepest horizontal gradients are located at the northern central massif front (Fig. 16a, b) and across the Windgällen-Färnigen zone (Fig. 16b, c), where we locally observe gradients up to $30\,^{\circ }\text{C/km}$. Along transect B, a relatively discrete temperature increase across the Windgällen-Färnigen zone from ca. $300\,^{\circ }\text{C}$ in the footwall to more than $400\,^{\circ }\text{C}$ in the hanging wall is observed (Fig. 16). Along transect C, a corresponding temperature jump across the Windgällen-Färnigen zone is also observable, but less pronounced. This is interpreted to reflect decreasing displacements along this major reverse fault from W to E. Along transect D, a sub-linear $T_p$ increase from north to south and a weakly pronounced local $T_p$ maximum close to the southern Aar Massif boundary can be observed. In map view (Fig. 16), the trend of isogrds is in good general agreement with mapped mineral-isograds, such as the biotite-in, stilpnomelane-out, microcline/sanidine isograds (Bambauer et al. 2005; Frey et al. 1976; Niggli and Niggli 1965). The first occurrence of dynamic quartz recrystallization (Bambauer et al. 2009; Voll 1976) closely follows the 300–320$\,^{\circ }\text{C}$ isograds.

Measured RSCM temperatures above the basal Helvetic thrust are systematically 10–40$\,^{\circ }\text{C}$ higher than adjacent samples from the Lower Helvetic (Figs. 16 and 17), which is in general agreement with Frey et al. (1976) and Frey (1980).

### Alpine metamorphic pattern in section view

Figure 17 depicts the distribution of $T_p$ along three stacked cross sections from east to west. Inferred isograds highlight the Alpine metamorphic pattern. Overall, isograds closely follow the domal shape of the Aar Massif. This is best observed along cross section C–C′ (Fig. 17a), where exposures of top basement and the spatial resolution of RSCM data are excellent. At the northern massif front, isograds plunge to the N with angles decreasing from $45^{\circ }$ in the central Aar Massif (Fig. 17c) to $20^{\circ }$ in the east (Fig. 17a), and are systematically $5^{\circ }-15^{\circ }$ steeper than top basement or the basal Helvetic thrust (Fig. 17). Across the Windgällen-Färnigen zone (Fig. 17c), we observe a relatively discrete temperature increase from $300\,^{\circ }\text{C}$ in the footwall to more than $400\,^{\circ }\text{C}$ in the hanging wall. This correlates with the significant vertical throw across the tectonic boundary and is in agreement with field observations of south-up dominated kinematics (Nibourel et al. 2018). Along section B′–B′′ (Fig. 17b) the Windgällen-Färnigen zone is best exposed. There, sub-vertical to overturned isograds spatially coincide with the position of the recumbent Windgällen fold-and-thrust structure. At the transition from the Aar Massif into the Urseren-Garvera zone or Clavaniev zone, isograds generally turn into a near horizontal or S-plunging orientation. No significant $T_p$ jump is observed across the Cavestrau detachment (samples TR-17-01/TR-17-02, Fig. 17a), the Clavaniev as well as the Urseren-Garvera zones (Figs. 16 and 17).

### Temperature-time history

**Fig. 19:**
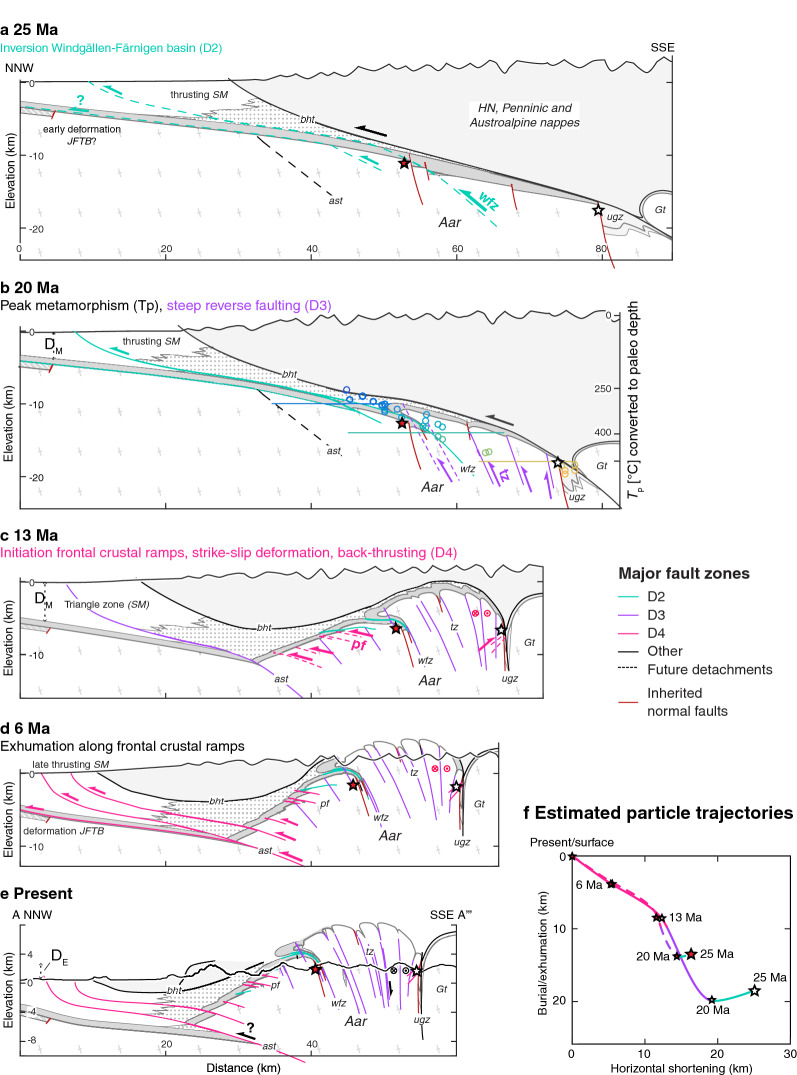
**a**–**e** Hypothetic kinematic evolution of the eastern Aar Massif since the Late Oligocene (25 Ma). Two key localities for which the temperature-time(-burial) history is shown on Fig. 18 are highlighted as red (northern Aar Massif) and white asterisks (southern Aar Massif), respectively. Trace of cross section A–A′′′ is given in Fig. 1a. Depth and orientation of top basement are based on depth-converted new and published temperature-time constraints, assuming a constant $25\,^{\circ }\text{C/km}$ thermal gradient through time (Nibourel et al. 2018). *JFTB*—Jura fold-and-thrust belt; *SM*—Subalpine Molasse; $D_{USM}$—Estimated thickness of Lower Freshwater Molasse (Pfiffner 2017); $D_M$—Estimated thickness of Molasse deposits at 13 Ma (Cederbom et al. 2011); $D_E$—Estimated eroded thickness of Molasse deposits at present (Cederbom et al. 2011). Legend and abbreviations as in Fig. 1a. **f** Estimated particle trajectories for two key localities (highlighted as asterisks in **a**–**e**) from 25 Ma to present, based on the tectonic reconstruction shown (**a**–**e**). Horizontal (relative to stable Europe) and vertical (relative to the surface)

Figure 18 shows the temperature-time evolution for two key localities in the northern and southern Aar Massif, respectively (localities are highlighted with an asterisk on Fig. 19). These data are based on published temperature-time constraints, stratigraphic considerations and new RSCM data (references are given in the caption to Fig. 18).

The upper age bound for peak metamorphism is given by the Rupelian (28–34 Ma) age of the youngest flysch units deposited onto the Lower Helvetic domain (Lu et al. 2018; Menkveld-Gfeller et al. 2016). According to thermal calculations (Challandes et al. 2008), approximately 10 Myrs are required to heat the Aar Massif basement to its maximum temperature of $450\,^{\circ }\text{C}$ reached in the south. In the southern central Aar Massif, the age of Alpine peak metamorphism is relatively well-constrained by Ar–Ar and K–Ar ages on syn-kinematic Alpine sheet silicates (Berger et al. 2017b; Challandes et al. 2008; Rolland et al. 2009). In the northern Aar Massif, the age of peak Alpine metamorphism is poorly constrained. This is mostly due to the scarcity of datable mineral phases and grain sizes and due to the complex polycyclic metamorphic history of the basement units in this area (Berger et al. 2017b). Furthermore, Alpine peak temperatures were not high enough to completely reset zircon fission track ages. The northernmost likely Alpine reset zircon fission track ages point to an initiation of exhumation at 17 to 27 Ma and indicate a relatively long duration of the metamorphic peak (Fig. 19, Michalski and Soom 1990; Nibourel 2019; Wangenheim 2016). Overall, these constraints and considerations suggest that the Alpine thermal peak occurred at $\sim 22$ to 17 Ma for both the northern and southern Aar Massif (Nibourel et al. 2018).

Peak temperatures reached $300\,^{\circ }\text{C}$ and $450\,^{\circ }\text{C}$ in the northern and southern Aar Massif, respectively. Assuming a geothermal gradient of $25\,^{\circ }\text{C/km}$ at peak temperature conditions (see Nibourel et al. 2018, and discussion of this assumption below), this corresponds to a maximum burial of 12 km in the north, increasing to 20 km in the south (Nibourel et al. 2018). This suggests mean exhumation rates of 0.6 mm/year and 1 mm/year for the past 20 Myrs for the northern and southern Aar Massif, respectively. From 17–14 Ma, we observed rapid cooling at rates up to $50\,^{\circ }\text{C/Ma}$ in the southern Aar Massif, while the northern Aar Massif cooled at $30\,^{\circ }\text{C/Ma}$. This implies significant differential uplift along the transect with S-block up dominated kinematics. At 14 Ma, the northern and the southern Aar Massif were exhumed to the same crustal level. From 14 Ma, onwards the northern and southern parts of the massif continued to cool at the same relatively constant rate ($\sim 20\,^{\circ }\text{C/Ma}$, see also Herwegh et al. 2017).

### Relative timing of deformation and peak temperature

The general pattern of isograds in map and section view reflects the three dimensional dome-shape of the Aar Massif in both massif-perpendicular and along-strike directions (Figs. 16 and 17). Our results suggest that most of the deformation and the associated development of the present-day structural relief occurred after peak temperature conditions (Nibourel et al. 2018), which is in general agreement with data and models in Burkhard (1988), Rahn and Grasemann (1999) and Glotzbach et al. (2010). This implies that the future Aar Massif was buried with minor internal deformation. Such an interpretation is also supported by the $10^{\circ }-15^{\circ }$ intersection angle between inferred isograds and top basement as well as the basal Helvetic thrust (Fig. 17). This suggests that both marker horizons, at peak temperature conditions, were dipping to the S at an angle of $10^{\circ }-15^{\circ }$ (if isochronous metamorphism and a constant geothermal gradient are assumed Nibourel et al. 2018). A discussion of these assumptions can be found below and in Nibourel et al. (2018).

The deformation structures described above are placed along the temperature-time path of the northern and southern Aar Massif (Fig. 18). This allows the subdivision into prograde, peak metamorphic and retrograde deformation.

The early northward thrusting of the southernmost Aar Massif cover (summarized as early Lower Helvetic thrust sheets on Fig. 17, affiliated to D1 on Fig. 18) pre-dates the thermal peak in the Lower Helvetic. This can be demonstrated by equivalent temperatures above and below the Cavestrau décollement in the southeastern Aar Massif (Fig. 17a). The estimated timing of active shearing along the basal Helvetic thrust (35–15 Ma; e.g., Akker 2020; Hunziker et al. 1986; Pfiffner 2015) is given on Fig. 18. It represents the main detachment above the Lower Helvetic and D1 is possibly related to slip along this major structure.

Basement-involved deformation initiated with the inversion of the Windgällen-Färnigen basin and with the development of other minor thrusts during D2. Across the Windgällen-Färnigen zone, isograds are less deformed than the basement-cover contact. This is interpreted to reflect some peak metamorphic deformation along this zone (i.e. relaxed isotherms after initial deformation). Also D2 structures are mostly obliterated by subsequent deformation, there is some evidence for peak metamorphic D2 deformation in the southern Aar Massif. In cross section C–C′ (Fig. 17a), inferred isograds are flat or $20^{\circ }$ S-plunging, but significantly less steep than the steeply S-dipping Gotthard nappe-Aar Massif boundary, which was presumably steepened already during D2. This points to deformation at peak metamorphic conditions or earlier, which is in agreement with observations of syn-kinematic peak metamorphic mineral assemblages in ductile shear zones in the central southern Aar Massif (Goncalves et al. 2012) as well as with post-kinematic but peak metamorphic allanite (Janots and Rubatto 2014) and the presence of syn- to post-kinematic chloritoid along the Urseren-Garvera zone (Niggli and Niggli 1965; Pfiffner 1972, 1978).

Overall, the $T_p$ pattern indicates that most of the pervasive and intense deformation associated with D3 occurred after peak temperature conditions (Figs. 16 and 17). This is best observed at the northern massif front, where progressive steepening of isograds correlates with an increasingly steep basement-cover contact (Fig. 17), as also highlighted by an increasingly tight spacing of isograds in map view (Fig. 16). D3 is likely the main responsible for the onset of rapid and differential exhumation in the Aar Massif domain (Fig. 18).

D4 thrusts are retrograde as confirmed by a relatively discrete temperature jump across the Pfaffenchopf thrust array (see Fig. 16a). Retrograde conditions are also indicated by the generally more discrete and brittle-ductile nature of D4 shear zones and associated mineral assemblages. D4 back-thrusts in the southern Aar Massif have the same characteristics (i.e., associated mineral phases) as D4 NNW-vergent thrusts in the north and hence developed at comparable temperature conditions. This implies no significant differential exhumation between the northern and the southern Aar Massif from D4 onwards.

The purely brittle characteristics of faults in basement and cover units indicate even lower temperatures during D5.

### Structural style and steepness of shear zones: a function of temperature?

We identify two zones with contrasting structural appearance. In the northern Aar Massif (footwall to the Windgällen-Färnigen zone, Figs. 2 and 3, subregion I, $T_p$ below $300\,^{\circ }\text{C}$), thrusts that developed during D2 and D4 dominate the structural appearance, while steep D3 structures and the associated foliation are weakly developed. South of the Windgällen-Färnigen zone (Figs. 2 and 3), where compressional deformation mostly occurred at temperatures $> 330\,^{\circ }\text{C}$, steep to sub-vertical ductile D3 shear zones and an associated greenschist facies foliation are the dominant structural element, while D2 and D4 thrusts are not or only weakly developed. Along the Windgällen-Färnigen zone (Figs. 2 and 3), D2, D3 and D4 structures are equally developed.

The steepness of active NNW-verging shear zones (estimated for the time they were active) appears to have varied through time. Estimated dip values increase from 25$^{\circ }$ to 30$^{\circ }$ during prograde to peak metamorphic thrusting (D2), to a dip of $50^{\circ }$ or more during peak metamorphic to retrograde reverse faulting (D3), then decreased again to 15$^{\circ }$–30$^{\circ }$ during retrograde thrusting (D4). Hence, both the style of compressional basement-involved deformation (i.e., steepness of shear zones) and the timing of the transition to basement-involved deformation appear to vary as a function of evolving temperature conditions.

Figure 19 shows how the steepness of top basement, inherited normal faults and D3 shear zones evolved through time. Based on this reconstruction, we estimate a dip $>50^{\circ }$ for D3 shear zones (at the time of active reverse faulting) and we suggest that the dip has not increased significantly during subsequent deformation. This is also supported by the fact that D3 shear zones were commonly reactivated as strike-slip faults. In contrast to any previous and subsequent deformation phase, D3 accommodated significant vertical S-block up movements, which produced the present-day top basement topography as well as the exposed metamorphic gradient. This phase of rapid uplift also coincides with a decrease in relative Europe-Adria convergence from 13 mm/year to 2 mm/year (Handy et al. 2010; Schmid et al. 1996). In a recent model, large vertical displacements during D3 were explained by buoyancy driven vertical tectonics related to the delamination of the lower European crust due to a rollback mechanism of the continental European lithosphere (see also Herwegh et al. 2017). Our results suggest that the upward movement of buoyant crustal material along steep reverse faults was facilitated by high temperature conditions weakening the crust and by a positive feedback mechanism with efficient erosional unloading (Nibourel et al. 2018). However, high temperature in itself is not sufficient as explanation. In the Lepontine region, dominant shearing and schistosity development is shallowly dipping but occurred at Amphibolite facies conditions (e.g., Maxelon and Mancktelow 2005). This points to the comparatively slow convergence rates (Schmid et al. 1996; Handy et al. 2010) as an additional driver for the large exhumation to shortening ratio during the Aar Massif exhumation.

### Inversion of inherited normal faults and associated half-grabens

The two major SSE-dipping inherited Färnigen and Tödi normal faults were not significantly reactivated during Alpine compressional deformation. Instead, decametre-wide steep ductile reverse shear zones developed in the basement below the adjacent syn-rift basins at an angle of only 5$^{\circ}$–20$^{\circ}$ to the inherited normal faults. These shear zones can be traced into steep to overturned limbs in the overlying Mesozoic strata. This suggests that the halfgraben synclinal shape acted as shape perturbation for initiating cuspate-lobate buckling at the rheological boundary between relatively competent basement and relatively weak cover units. In the unlayered, more isotropic basement, this may have been accommodated by localised shear zones, which led to the formation of tight to isoclinal pinched synclines, where the half-graben filled with weaker sediments provided a shape perturbation and to open anticlines in the hanging wall to theses shear zones. Such an interpretation implies that the basement shear zones nucleated at the basement-cover interface. Alternatively, the shear zones could have nucleated at depth, propagate upward and cause the syn- and antiforms at the basement-cover interface. As demonstrated above, D3 structures, such as the main shear zone of the Tödi zone, were active mostly after the metamorphic peak which in this area corresponds to $330\,^{\circ }\text{C}$ to $360\,^{\circ }\text{C}$.

Two main factors may have inhibited the reactivation of the normal faults. First, at initiation of basement-involved shearing, the normal faults had dip angles of 70$^{\circ}$–80$^{\circ}$ (see tectonic reconstruction on Fig. 19a), and were thus too steep to be reactivated as a reverse fault. Second, a number of observations point to a decreasing rheological contrast between the relatively strong basement and the relatively weak cover sediments and inherited normal faults as a function of temperature: (i) the absence of major detachments between basement and cover units, (ii) distributed ductile deformation along D3 basement shear zones, (iii) cuspate-lobate fold geometries along the basement cover contact (Ramsay and Huber 1987). The low angle between inherited normal faults and D3 shear zones ($< 15^{\circ }$) suggests that thermal weakening of basement and cover units, possibly enhanced by hydration and changes in mineralogy (Oliot et al. 2010), is mainly responsible for the absence of normal fault reactivation. This is in agreement with observations from other ECMs in the Western Alps where new basement shear zones formed in the basement instead of reactivating inherited weaknesses such as pre-Alpine foliations or normal faults (Bellahsen et al. 2012; Bellanger et al. 2015; Boutoux et al. 2016; Lafosse et al. 2016). Rather, the normal faults acted as buttresses (Tricart and Lemoine 1986), as highlighted by near coaxial deformation in the half-grabens adjacent to the normal faults. Numerical studies (Buiter and Pfiffner 2003; Buiter et al. 2009) have suggested that the rheological contrast between relatively weak basin fill and the underlying basement controls the localisation of compressional deformation, which is in general agreement with our field observations.

### Thermo-kinematic scenario for the development of the eastern Aar Massif

We propose a kinematic scenario for the development of the eastern Aar Massif (Fig. 19a–e) from 25 Ma to present. It is based on the following assumptions: (i) constant line length for top basement, (ii) constant area for the crustal basement (assuming an undeformed crustal thickness of 30 km and no out-of-section movement), (iii) a constant $25\,^{\circ }\text{C/km}$ geothermal gradient in space and time, (iv) isochronous metamorphism in the footwall of the basal Helvetic thrust and (v) constant topography over the past 20 Ma (Campani et al. 2012). A discussion of these assumptions can be found below and in Nibourel et al. (2018).

Figure 19f shows particle trajectories for two key localities (highlighted with a red and a white asterisk in Fig. 19a–e) from 25 Ma to present, based on the tectonic reconstruction. It highlights the components of horizontal (relative to stable Europe) and vertical (relative to the surface) displacements during the formation of the eastern Aar Massif (positive values indicate movement towards the surface, respectively NNW-directed movement).

Restoring pre-deformational geometries based on the line length of one horizon involves significant uncertainties, particularly when ductile shearing is involved (Boutoux et al. 2014). Out-of-section movements, especially at the presence of strike-slip faults during D4 are not considered in this reconstruction, which certainly adds some uncertainty to our model. Our field data indicate, however, that strike-slip faults are less abundant and associated displacements less significant than in the western and central Aar Massif (Belgrano et al. 2016; Steck 1968; Wehrens et al. 2017).

In the tectonic reconstruction (Fig. 19), the depth and orientation of marker horizons and corresponding estimates of shortening and exhumation rates strongly depend on the assumption of a constant and linear $25\,^{\circ }\text{C/km}$ geothermal gradient. This assumption is justified by the observed spacing of $T_p$ isograds in section view (Fig. 17) and by independent pressure-temperature constraints (Challandes et al. 2008; Goncalves et al. 2012; Rolland et al. 2009). However, tectonic movements towards or away from the surface, such as tectonic burial or subsequent exhumation are likely to perturb the isotherm pattern (Mancktelow and Grasemann 1997). At peak metamorphic conditions, the Lower Helvetic domain was situated in the footwall to the basal Helvetic thrust representing the main active tectonic boundary at that time (Pfiffner 2015). Numerical models by Molnar and England (1990) and Shi and Wang (1987) predict that thermal perturbations across actively deforming thrusts re-equilibrate rapidly. Especially for shallow dipping thrusts with slip rates smaller than 5 mm/year such as the basal Helvetic thrust (Nibourel et al. 2018; Pfiffner 2015). Additionally, the reference time chosen pre-dates most of the crustal thickening and uplift of the Aar Massif basement. Although minor temperature perturbations cannot be excluded, no significant vertical displacements, and as a consequence no major temperature perturbations are to be expected for this time step. In contrast, subsequent exhumation rates of 1 mm (Nibourel et al. 2018; Nibourel 2019) have likely compressed isograds towards the surface (Mancktelow and Grasemann 1997). Such perturbations are not considered in this reconstruction, which may lead to an underestimation of the peak rates at the initiation of rapid exhumation during early D3 deformation and/or to an overestimation of exhumation rates during the subsequent decrease of exhumation (D4).

To reconstruct the depth of top basement at peak temperature conditions, we infer that Alpine peak metamorphism in the Lower Helvetic was isochronous. Although there are significant gaps in the age record, we do overall not find clear evidence for significant diachrony in the metamorphic evolution as would be expected from an in sequence development. Rather, the ages discussed above and given in Fig. 18 point to one relatively isochronous metamorphic peak event between 22 and 17 Ma, which justifies this assumption. Similar observations were also made in other ECMs (e.g., Boutoux et al. 2016).

The temperature pattern, and as a consequence the reconstructed depth and orientation of top basement, could be affected by shear heating in locally strongly deformed sediments or by increased structural order due to aseismic shear (e.g., Kedar et al. 2020). Almost equivalent peak temperatures in directly adjacent internally weakly deformed sandstones and strongly sheared limestones indicate that the effect of strain did not have a major impact on most of our samples.

#### 25–20 Ma

Tectonic burial of the Aar Massif initiated after the deposition of the Matt Formation (34–28 Ma, Lu et al. 2018; Menkveld-Gfeller et al. 2016). Underthrusting in the footwall of the basal Helvetic thrust presumably formed the first foliation S1 in the cover of the Aar Massif. Figure 19a shows the Aar Massif at the initiation of basement-involved deformation (i.e., inversion of the Windgällen-Färnigen basin, as well as other early D2 thrusts). The timing of early thrusting and folding is estimated at 25 Ma, but is poorly constrained by zircon fission track ages and (U-Th)/He ages on zircon between 27 and 19 Ma along the northern Aar Massif (Michalski and Soom 1990; Nibourel 2019; Wangenheim 2016). D2 thrusts caused 7 km of massif-internal shortening but did not significantly exhume the Aar Massif (Fig. 19f). The absence of significant exhumation is attributed to ongoing underthrusting in the footwall to the basal Helvetic thrust and to the relatively low dip of early thrusts. Isograds across the sub-vertical Urseren-Garvera and the Clavaniev zones are near horizontal (Fig. 17). This indicates significant shearing already during the thermal peak in the southern Aar Massif. D2 thrusting was coeval with the initiation of thrusting in the Subalpine Molasse in late Oligocene to early Miocene times (Fig. 19b, Kempf et al. 1999; Schlunegger et al. 1997) and possibly with early deformation in the Jura fold-and-thrust belt as indicated by some equivocal evidence for fault activity during sedimentation around 20 Ma (Becker 2000).

#### 20–13 Ma

Figure 19b shows the Aar Massif at peak temperature conditions. In the retrodeformed profile sketch, $T_p$ data are used to reconstruct the depth and orientation of top basement. The recumbent Windgällen fold-and-thrust structure (D2) is overprinted by steep reverse faults and an associated pervasive foliation (D3). The timing of D3 shearing is dated at 22–14 Ma based on syn-kinematic growth of sheet silicates (Berger et al. 2017b; Challandes et al. 2008; Rolland et al. 2009) as well as monazites and adularia from associated clefts (Bergemann 2017; Ricchi et al. 2019). D3 caused intense massif-internal deformation and led to the development of the highly localised, massif-internal structural relief as observed in Fig. 19c. D3 was associated with 7 km of massif-internal shortening and $\sim 11\,\text {km}$ exhumation, which corresponds to a ratio vertical to horizontal displacement $>1.5$ (Fig. 19f, southern Aar Massif). This large vertical component was accommodated by D3 reverse faults. Our field data reveal that these reverse faults were steeper than $50^{\circ }$ already at inception. Such a high dip could (i) point to buoyancy-driven vertical tectonics related to the delamination of the lower crust in response to a rollback mechanism in the European continental lithosphere (Herwegh et al. 2017, 2020) and (ii) to a positive feedback mechanism between tectonically-driven uplift and erosion (Nibourel et al. 2018). Additionally, this period with a high uplift component coincides with an estimated decrease of Europe-Adria convergence at ca. 20 Ma (Handy et al. 2010; Schmid et al. 1996). Although D3 bends and exhumes the basal Helvetic thrust at the massif-scale, coeval activity of the basal Helvetic thrust and D3, at least during early D3 deformation, is indicated by the local bending of D2 and D3 structures towards tectonically higher levels (Fig. 3a, see also Pfiffner et al. 2011). In the central Aar Massif, D3 leads to a first stage of steepening of the basement-cover contact at the northern massif-front (including the reorientation of earlier D2 faults).

#### 13–6 Ma

Fig. 19c shows the Aar Massif at the transition to D4. At the northern massif front, D4 is dominated by arrays of NNW-verging thrusts (referred to as Pfaffenchopf phase in the central Aar Massif, e.g., Herwegh et al. 2020). Massif-internally and in the south, D4 is associated with oblique to strike-slip faulting (Oberaar phase in the central Aar Massif, e.g., Herwegh et al. 2020), coeval with back-thrusting in the southern Aar Massif. At 13 Ma, the two reference points in the northern and southern Aar Massif were exhumed to the same temperature, as constrained by constant zircon fission track ages across the transect (Nibourel 2019; Michalski and Soom 1990; Wangenheim 2016). From 13 Ma onwards, the two reference points share a common cooling history (Fig. 18). This is attributed to “en bloc” exhumation in the hanging wall to frontal crustal ramps, and implies no significant massif-internal compressional deformation after 13 Ma. D4 back-thrusts did not cause significant uplift of the Aar Massif with respect to the Tavetsch and Gotthard nappes as shown by relatively constant cooling ages across the Clavaniev and Urseren-Garvera zones (see also Glotzbach et al. 2010; Nibourel 2019). “En bloc” exhumation from 13 Ma onwards is in agreement with the observed transition from reverse-fault dominated to strike-slip-dominated deformation in the massif-internal areas. In the central Aar Massif, the transition to strike-slip dominated shearing is dated at $\sim 14\,\text {Ma}$ (e.g., Rolland et al. 2009). At 13 Ma, the Aar Massif basement had not reached the surface, which is in agreement with the absence of Aar Massif pebbles in the youngest (11.5 Ma, Rahn and Selbekk 2007) Upper Freshwater Molasse depostis (Pfiffner 2015). The frontal crustal ramps, which developed during D4 are coeval with the main deformation event at the Jura fold-and-thrust belt (14–4 Ma, Becker 2000; Looser et al. 2020) and break-back thrusting in the Subalpine Molasse, which lasted until at least 5 Ma (Mock and Herwegh 2017; Mock et al. 2020; von Hagke et al. 2014).

#### 6 Ma to present

Figure 19d shows an in sequence development of frontal crustal ramps above which the Aar Massif continued to exhume at a relatively constant rate (0.5 mm/year). Continued exhumation in the hanging wall of younger D4 thrusts lead to the exposure of older D4 thrusts along the northern central massif front (i.e., Pfaffenchopf thrust, Herwegh et al. 2020). Exhumed D4 thrusts have dip angles between 15 and $30^{\circ}$ (Fig. 19e). About 13 km of massif-internal shortening were accommodated mainly along D4 thrusts and caused up to 9 km of uplift and erosion between 13 Ma and present (Fig. 19c–e).

#### Discussion of tectonic reconstruction

According to our tectonic reconstruction, shortening rates within the Lower Helvetic domain were relatively constant (1 mm/year) between 25 Ma and present. Exhumation rates $>1 \text { mm/year}$ are estimated for the time interval between 22 Ma and 14 Ma during D3 reverse faulting. During subsequent D4 (12 Ma to present), exhumation rates decreased to a mean value of 0.5 mm/year. For the time span between 16 Ma and present, these exhumation rates are in good agreement with Glotzbach et al. (2010) and with inverse model results presented in Nibourel (2019). Rosenberg et al. (2015) estimated exhumation rates of 0.8 mm/year for the Gotthard nappe/Aar Massif, averaged over the past 30 Ma. Detrital thermochronology studies suggest steady exhumation since at least 15 Ma at rates between 0.4 and 1 mm/year for the Lepontine area (e.g., Bernet et al. 2001; Glotzbach et al. 2011a). Exhumation estimates from this study are similar or higher than estimates from other, mostly thermochronology-based studies. This discrepancy could arise from the fact that thermochronology based studies generally cannot capture early stages of exhumation (i.e., exhumation between peak metamorphic conditions and the first reset thermochronometer).

The estimated 27 km of massif-internal shortening are in good agreement with Rosenberg et al. (2015) and Schmid et al. (1996). In the western Aar Massif, Burkhard (1988) and Bellahsen et al. (2014) estimated more than twice the amount of shortening (69 km). This large discrepancy arises mainly from the interpretation of all shear zones as shallow dipping thrusts (Burkhard 1988).

The Jungfrau syncline and the Doldenhorn nappe in the western Aar Massif (Krayenbuhl and Steck 2009) have many similarities to the Windgällen-Färnigen zone. Both zones are related to an inherited normal fault and an associated half-graben. These half grabens were inverted during an early phase of NNW-vergent basement-involved shearing, bringing the crystalline basement on top of what is referred to as the Gastern Massif in the west and the northern Aar Massif in our study area, respectively (Fig. 3b, c). In the western Aar Massif, this early inversion and juxtaposition (D2 in this study) is referred to as Kiental phase (Burkhard 1988; Mair et al. 2018, Table 1). The reorientation and southward steepening of the Jungfrau syncline/Doldenhorn nappe and associated early foliations are explained by a passive backward rotation in the hanging wall to younger structures due to an in sequence development (Krayenbuhl and Steck 2009). According to Burkhard (1988), the uplift of the Gastern Massif and the related exhumation and tilting of the Aar Massif in its hinterland are summarized as Grindelwald phase, which corresponds to D3 in our study area. Our observations demonstrate that D3 shear zones of the Massif-internal regions were steep already at inception with very minor subsequent back-rotation (see also Herwegh et al. 2017), which is in contrast to the above interpretation.

In the central Aar Massif, steep to sub-vertical Handegg phase reverse faults are described as the first discernible Alpine deformation structures (e.g., Wehrens et al. 2017, see also Table 1). These faults correlate with D3 structures in our study area. In contrast, Labhart (1966) and Mair et al. (2018) described moderately steep pre-D3 (or pre-Handegg phase) foliations in the Mesozoic sediment wedges of the northern Aar Massif front, very similar to what we described as S2 in this study. Pre-Handegg phase structures likely also exist in the central Aar Massif, but cannot be recognised in the crystalline basement.

The post-D3 structural development in the study area is very similar to what is known from the central Aar Massif (e.g., Wehrens et al. 2017). At the northern massif front, D4 shallow dipping thrusts represent the equivalent to what is known as Pfaffenchopf phase structures in the central Aar Massif and the Jungfrau area (Herwegh et al. 2020; Mair et al. 2018, Table 1). Massif-internally, D4 structures of the study area are dominated by oblique to strike-slip shear zones, which is equivalent to what is referred to as Oberaar phase in the central Aar Massif (Wehrens et al. 2017, Table 1). However, the abundance of strike-slip faults and associated displacements gradually decrease towards the eastern Aar Massif. This is in contrast to the southern domains of the central and western Aar Massif, where oblique to strike-slip faults locally dominate the structural appearance (e.g., Steck 1968; Wehrens et al. 2017). This W-E decrease could be due to a kinematic link between the central/western Aar Massif and the Simplon-Rhône fault system. Furthermore, given their E-W strike, many dextral strike-slip faults, which dominate in the Grimsel area, may join the Urseren-Garvera or Clavaniev zones already west of Andermatt (Figs. 1, 2 and 3; Egli et al. 2018; Rolland et al. 2009; Wehrens et al. 2017). This hypothesis is also supported by dextral strike-slip movements along the Clavaniev zone (e.g., Gisler 2018) and associated K–Ar ages (10–14 Ma, Pleuger et al. 2012), which are similar to the age estimated for Oberaar phase structures in the central Aar Massif (Bergemann 2017; Berger et al. 2017b; Rolland et al. 2009).

East of the study area, the main Alpine foliation and associated sub-parallel shear zones are referred to as Calanda phase structures (Milnes and Pfiffner 1977). Milnes and Pfiffner (1977) proposed a late Oligocene age for the Calanda phase, mostly based on relative age constraints. Calanda phase deformation coincided with peak metamorphic temperatures, as indicated by syn- to post-kinematic chloritoid crystals (Milnes and Pfiffner 1977). The age of peak metamorphism in this area was dated at 18–24 Ma, based on K-Ar ages on illite (Frey et al. 1974; Milnes and Pfiffner 1977). According to Milnes and Pfiffner (1977), the Calanda phase foliation is characterised by a pronounced southward and downward steepening, similar to what we observed for D2 (and partly D3) in our study area. The Calanda phase foliation is overprinted by a steeper SSE dipping crenulation cleavage (Ruchi phase, Milnes and Pfiffner 1977), which appears to be related to movements along the basal Helvetic thrust. The Calanda and Ruchi phases involve basement and cover units (Milnes and Pfiffner 1977). Based on the above equivocal age and geometrical constraints, we correlate the Calanda phase with D2 (Table 1). The Ruchi phase is correlated with D3, which is always steeper than D2 and commonly forms a crenulation cleavage. However, in the absence of clear age constraints, this correlation mostly based on geometrical considerations remains unsure (Milnes and Pfiffner 1977).

The thermo-kinematic evolution of the Mont Blanc - Aiguilles Rouges Massifs, shows a number of similarities with the evolution of the Aar Massif, but also some differences (Boutoux et al. 2016). Girault et al. (2020) found significant peak metamorphic and retrograde deformation in the Lower Helvetic Doldenhorn (western Aar Massif) and Morcles (eastern Mont Blanc - Aiguilles Rouges Massifs) nappes. In the Mont Blanc and Aiguilles Rouges Massifs, massif-internal deformation appears to mostly coincide with the thermal peak, which persisted for 5–10 Myrs (Boutoux et al. 2016). This is in contrast to the Aar Massif where most of the deformation occurred after the thermal peak and where the thermal peak lasted for about 5 Myrs (southern Aar Massif). Indications for a longer duration of the thermal peak are found in the northern Aar Massif (Herwegh et al. 2020; Nibourel 2019).

We observe a distinct deformation sequence starting with basement-involved prograde to peak metamorphic thrusting (D2), followed by reverse fault-dominated pervasive massif-internal deformation during and after the thermal peak (D3), and finally the exhumation of the massif in the hanging wall of frontal crustal ramps (D4). Similar observations were reported from other ECMs (e.g., Bellahsen et al. 2012; Boutoux et al. 2016) and orogens (i.e., Scandinavian Caledonides, Torgersen et al. 2018) suggesting that this is characteristic for the kinematic evolution of late-stage orogens. The geodynamic framework and associated temperature distribution emerge as first order parameters controlling the deformation style. Structural observations indicate a weak rheological contrast between basement and cover units at greenschist facies conditions. Although inherited normal faults were not significantly reactivated, they dictated the position of compressional deformation, which preferentially localised below rift-related basins. A similar mechanism is described in Bauville and Schmalholz (2015) and Krayenbuhl and Steck (2009).

## Conclusions

This study demonstrates that RSCM peak temperature data, coupled with detailed structural analyses and independent age constraints, can be used to quantify vertical and horizontal components of collisional deformation and to study the mechanical behavior of basement and cover units during the late-stage orogenic evolution.

The future Aar Massif was buried to depths up to 20 km with minor massif-internal deformation (D1). This clearly prograde deformation only affected the cover of the Aar Massif. In the eastern Aar Massif, early prograde to peak metamorphic thrusts (D2) caused 7 km of massif-internal basement-involved shortening but did not cause significant uplift, which is attributed to the low fault dip and to ongoing underthrusting in the footwall to the basal Helvetic thrust. At peak temperature conditions ($\sim$ 20 Ma), top basement and the overlying basal Helvetic thrust were dipping $10^{\circ }-15^{\circ }$ to the SSE as highligthed by a sub-linear trend of retrodeformed and depth-converted $T_p$ data increasing from $250\,^{\circ }\text{C}$ in the north to more than $500\,^{\circ }\text{C}$ in the Urseren-Garvera zone. D3 ($\sim$ 22–14 Ma) was dominated by steep to sub-vertical ductile basement shear zones. These reverse faults bend peak metamorphic isograds at the massif scale, and across major shear zones, thus indicating mostly post-metamorphic deformation. Field investigations show that D3 shear zones were steep already at inception. Reverse S-block up dominated movements along these shear zones caused rapid ($\sim 1.4\,\text {mm/year}$) and highly localised exhumation, which produced the characteristic massif-internal top basement geometry. After 14 Ma (D4), the Aar Massif continued to exhume at slower rates ($\sim 0.5\,\text {mm/year}$) in the hanging wall of frontal crustal ramps for which we estimate a dip of $15^{\circ }-30^{\circ }$. D4 structures are clearly retrograde, as highlighted by their more brittle and discrete appearance and by discrete $T_p$ jumps across major structures such as the Pfaffenchopf thrust array. Overall, estimated shortening rates remained relatively constant ($\sim 0.5\,\text {mm/year}$) through the last 25 Ma of Aar Massif deformation.

We observe a distinct change in structural style between the northern Aar Massif ($T_p < 300\,^{\circ }\text{C}$), where thrusts dominate the structural appearance and the southern Aar Massif ($T_p >330\,^{\circ }\text{C}$), where steep reverse faults are the dominant structural element. In contrast to early prograde (D2) and late retrograde (D4) thrusts, peak- to post-metamorphic D3 reverse faults appear to have initiated at a much higher dip angle ($> 50^{\circ }$). Inherited normal faults were not significantly reactivated. Instead, D3 shear zones developed in the basement below inherited rift-basins and at an acute angle ($5^{\circ }-20^{\circ }$) to the basin-bounding normal faults. The sedimentary cover was disharmonically folded, but mostly remained attached to its crystalline substratum. Altogether, these observations point to a decreasing rheological contrast between relatively strong basement units and relatively weak cover units and inherited normal faults with increasing temperature conditions, especially above temperatures of $330\,^{\circ }\text{C}$. Although deformation is certainly driven by relative convergence or buoyancy forces, temperature appears to control the timing and style (shear zone dip) of basement-involved deformation, while inherited rift-basins dictate the position where thick-skinned deformation will initiate. Such an evolution appears to be characteristic for late-stage orogens.

## Supplementary Information

**Additional file 1:** **Table S1** contains a list of geological maps and cross sections used for the shear zone map shown on Figure 2a. **Table S2** contains RSCM data and sample information. **Figures S1–S4** contain histograms showing the distribution of RSCM peak temperature data for each sample.

## Data Availability

All data generated or analysed during this study are included in this published article and its supplementary information files. Analysed rock samples and thin sections are stored at the collection of the Institute of Geological Sciences, University of Bern.

## References

[CR1] Abrecht J (1994). Geologic units of the Aar massif and their preâ€“Alpine rock associations: a critical review: the pre-Alpine crustal evolution of the Aar-, Gotthard-and Tavetsch massifs. Schweizerische mineralogische und petrographische Mitteilungen.

[CR2] Akker, I. V. (2020). *The evolution of slate microstructures during the accretion of foreland basin sediments and implications for mechanical strength, fluid flow and seismicity in accretionary wedges*. Ph.D thesis, unpublished, Universität Bern.

[CR3] Ambühl E, Huber HM, Niggli E, Huber W, Niggli M, Flück W (2008). Geological Atlas of Switzerland 1:25 000, map sheet 126 Oberalppass.

[CR4] Baker, D. (1964). *Structural petrology of the Windgällen fold*. Ph.D. thesis, unpublished, University of Zürich.

[CR5] Baltzer A (1880). Das Aarmassiv (mittlerer Theil) nebst einem Abschnitt des Gotthardmassivs. Beiträge zur Geologischen Karte der Schweiz.

[CR6] Bambauer HU, Bernotat W, Breit U, Kroll H (2005). Perthitic alkali feldspar as indicator mineral in the Central Swiss Alps. Dip and extension of the surface of the microcline/sanidine transition isograd. European Journal of Mineralogy.

[CR7] Bambauer HU, Herwegh M, Kroll H (2009). Quartz as indicator mineral in the Central Swiss Alps: the quartz recrystallization isograd in the rock series of the northern Aar massif. Swiss Journal of Geosciences.

[CR8] Barzoi SC (2015). Shear stress in the graphitization of carbonaceous matter during the low-grade metamorphism from the northern Parang Mountains (South Carpathians): Implications to graphite geothermometry. International Journal of Coal Geology.

[CR9] Baumberger, R. (2015). *Quantification of lineaments: Link between internal 3D structure and surface evolution of the Hasli valley (Aar Massif, Central Alps, Switzerland)*. PhD thesis, unpublished, Universität Bern.

[CR10] Bauville A, Schmalholz SM (2015). Transition from thin-to thick-skinned tectonics and consequences for nappe formation: numerical simulations and applications to the Helvetic nappe system, Switzerland. Tectonophysics.

[CR11] Beaumont C, Muñoz JA, Hamilton J, Fullsack P (2000). Factors controlling the Alpine evolution of the central Pyrenees inferred from a comparison of observations and geodynamical models. Journal of Geophysical Research: Solid Earth.

[CR12] Becker A (2000). The Jura Mountains: An active foreland fold-and-thrust belt?. Tectonophysics.

[CR13] Belgrano TM, Herwegh M, Berger A (2016). Inherited structural controls on fault geometry, architecture and hydrothermal activity: An example from Grimsel Pass Switzerland. Swiss Journal of Geosciences.

[CR14] Bellahsen N, Jolivet L, Lacombe O, Bellanger M, Boutoux A, Garcia S, Mouthereau F, Le Pourhiet L, Gumiaux C (2012). Mechanisms of margin inversion in the external Western Alps: Implications for crustal rheology. Tectonophysics.

[CR15] Bellahsen N, Mouthereau F, Boutoux A, Bellanger M, Lacombe O, Jolivet L, Rolland Y (2014). Collision kinematics in the western external Alps. Tectonics.

[CR16] Bellanger M, Augier R, Bellahsen N, Jolivet L, Monié P, Baudin T, Beyssac O (2015). Shortening of the European Dauphinois margin (Oisans Massif, Western Alps): New insights from RSCM maximum temperature estimates and 40Ar/39Ar in situ dating. Journal of Geodynamics.

[CR17] Bergemann, C. (2017). *Crystallization age of alpine cleft monazite-(Ce) and correlation with tectonically driven hydrothermal dissolution/precipitation events*. Ph.D. thesis, unpublished, University of Geneva.

[CR18] Bergemann C, Gnos E, Berger A, Whitehouse M, Mullis J, Wehrens P, Pettke T, Janots E (2017). Th-Pb ion probe dating of zoned hydrothermal monazite and its implications for repeated shear zone activity: An example from the Central Alps. Switzerland. Tectonics.

[CR19] Berger A, Engi M, Erne-Schmid S, Glotzbach C, Spiegel C, de Goede R, Herwegh M (2020). The relation between peak metamorphic temperatures and subsequent cooling during continent-continent collision (western central alps, switzerland). Swiss journal of geosciences.

[CR20] Berger, A., Mercolli, I., Herwegh, M., and Gnos, E. (2016). Geological Map of the Aar Massif, Tavetsch and Gotthard Nappes, 129, 1:100 000. *Special Geological Maps, Federal Office of Topography swisstopo, Wabern*.

[CR21] Berger, A., Mercolli, I., Herwegh, M., and Gnos, E. (2017a). Geological Map of the Aar Massif, Tavetsch and Gotthard Nappes, 129, 1:100 000, Explanatory notes. *Special Geological Maps, Federal Office of Topography swisstopo, Wabern*.

[CR22] Berger A, Wehrens P, Lanari P, Zwingmann H, Herwegh M (2017). Microstructures, mineral chemistry and geochronology of white micas along a retrograde evolution: An example from the Aar massif (Central Alps, Switzerland). Tectonophysics.

[CR23] Bernet M, Zattin M, Garver JI, Brandon MT, Vance JA (2001). Steady-state exhumation of the European Alps. Geology.

[CR24] Beyssac O, Goffé B, Chopin C, Rouzaud J (2002). Raman spectra of carbonaceous material in metasediments: A new geothermometer. Journal of Metamorphic Geology.

[CR25] Beyssac O, Lazzeri M (2012). Application of raman spectroscopy to the study of graphitic carbons in the earth sciences. Applications of Raman spectroscopy to earth sciences and cultural heritage. EMU Notes in Mineralogy.

[CR26] Böhm, C. (1986). *Geologie und Petrographie im Gebiet von Val Russein und Val Gliems, Graubünden*. Diplomarbeit, unpublished, Universität Bern.

[CR27] Bonanomi Y, Dietler T, Etter U (1992). Querschnitt zwischen dem südlichsten Aar-Massiv und der Lucomagno-Decke im Bereich des Gotthard-Basistunnels. Eclogae Geologicae Helvetiae.

[CR28] Bousquet R, Oberhänsli R, Goffé B, Wiederkehr M, Koller F, Schmid SM, Schuster R, Engi M, Berger A, Martinotti G (2008). Metamorphism of metasediments at the scale of an orogen: A key to the Tertiary geodynamic evolution of the Alps. Geological Society.

[CR29] Bousquet, R., Oberhänsli, R., Schmid, S., Berger, A., Wiederkeher, M., Robert, C., Möller, A., Rosenberg, C., Zeilinger, G., Molli, G., et al. (2012). *Metamorphic framework of the Alps-Carte metamorphique des Alpes CCGM/CGMW*. CCGM/CGMW.

[CR30] Boutoux A, Bellahsen N, Lacombe O, Verlaguet A, Mouthereau F (2014). Inversion of pre-orogenic extensional basins in the external Western Alps: Structure, microstructures and restoration. Journal of Structural Geology.

[CR31] Boutoux A, Bellahsen N, Nanni U, Pik R, Verlaguet A, Rolland Y, Lacombe O (2016). Thermal and structural evolution of the external Western Alps: Insights from (U-Th-Sm)/He thermochronology and RSCM thermometry in the Aiguilles Rouges/Mont Blanc massifs. Tectonophysics.

[CR32] Boyer SE, Elliott D (1982). Thrust systems. AAPG Bulletin.

[CR33] Breitschmid A (1982). Diagenese und schwache Metamorphose in den sedimentären Abfolgen der Zentralschweizer Alpen (Vierwaldstätter See, Urirotstock). Eclogae Geologicae Helvetiae.

[CR34] Brückner, W. and Zbinden, P. (1987). Geological Atlas of Switzerland 1:25 000, map sheet 83 Schächental, Explanatory notes. *Federal Office of Topography swisstopo, Wabern*.

[CR35] Buiter SJH, Pfiffner OA (2003). Numerical models of the inversion of half-graben basins. Tectonics.

[CR36] Buiter SJH, Pfiffner OA, Beaumont C (2009). Inversion of extensional sedimentary basins: A numerical evaluation of the localisation of shortening. Earth and Planetary Science Letters.

[CR37] Burkhard M (1988). L’Helvétique de la bordure occidentale du massif de l’Aar (évolution tectonique et métamorphique). Eclogae Geologicae Helvetiae.

[CR38] Burkhard M (1990). Aspects of the large-scale Miocene deformation in the most external part of the Swiss Alps (Subalpine Molasse to Jura fold belt). Eclogae Geologicae Helvetiae.

[CR39] Burkhard, M. (1999). Strukturgeologie und Tektonik im Bereich AlpTransit. In *Vorerkundung und Prognose der Basistunnels am Gotthard und am Lötschberg, Löw, S. and R. Wyss (eds)*, pages 45–57.

[CR40] Burkhard M, Sommaruga A (1998). Evolution of the western Swiss Molasse basin: structural relations with the Alps and the Jura belt. Geological Society.

[CR41] Burov E, Yamato P (2008). Continental plate collision, P-T-t-z conditions and unstable vs. stable plate dynamics: Insights from thermo-mechanical modelling. Lithos.

[CR42] Butler RWH, Tavarnelli E, Grasso M (2006). Structural inheritance in mountain belts: An Alpine-Apennine perspective. Journal of Structural Geology.

[CR43] Campani M, Mulch A, Kempf O, Schlunegger F, Mancktelow N (2012). Miocene paleotopography of the Central Alps. Earth and Planetary Science Letters.

[CR44] Capitanio F, Morra G, Goes S, Weinberg R, Moresi L (2010). India-Asia convergence driven by the subduction of the Greater Indian continent. Nature Geoscience.

[CR45] Cardello GL, Mancktelow NS (2014). Cretaceous syn-sedimentary faulting in the Wildhorn Nappe (SW Switzerland). Swiss Journal of Geosciences.

[CR46] Cederbom CE, van der Beek P, Schlunegger F, Sinclair HD, Oncken O (2011). Rapid extensive erosion of the North Alpine foreland basin at 5–4 Ma. Basin Research.

[CR47] Challandes N, Marquer D, Villa IM (2008). PTt modelling, fluid circulation, and 39 Ar-40 Ar and Rb-Sr mica ages in the Aar Massif shear zones (Swiss Alps). Swiss Journal of Geosciences.

[CR48] Choukroune, P. and Gapais, D. (1983). Strain pattern in the Aar Granite (Central Alps): Orthogneiss developed by bulk inhomogeneous flattening. In *Strain Patterns in Rocks, Cobbold, W.M. Schwerdtner and S.H. Treagus (eds)*, pages 411–418. Elsevier.

[CR49] Cloos M (1993). Lithospheric buoyancy and collisional orogenesis: Subduction of oceanic plateaus, continental margins, island arcs, spreading ridges, and seamounts. Geological Society of America Bulletin.

[CR50] Dielforder A, Berger A, Herwegh M (2016). The accretion of foreland basin sediments during early stages of continental collision in the European Alps and similarities to accretionary wedge tectonics. Tectonics.

[CR51] Dielforder A, Vollstaedt H, Vennemann T, Berger A, Herwegh M (2015). Linking megathrust earthquakes to brittle deformation in a fossil accretionary complex. Nature Communications.

[CR52] Dollfus SBA (1965). Ueber den helvetischen Dogger zwischen Linth und Rhein. Eclogae Geologicae Helvetiae.

[CR53] Ebert A, Herwegh M, Berger A, Pfiffner A (2008). Grain coarsening maps for polymineralic carbonate mylonites: A calibration based on data from different Helvetic nappes (Switzerland). Tectonophysics.

[CR54] Egli D, Baumann R, Küng S, Berger A, Baron L, Herwegh M (2018). Structural characteristics, bulk porosity and evolution of an exhumed long-lived hydrothermal system. Tectonophysics.

[CR55] England P, Molnar P (1993). The interpretation of inverted metamorphic isograds using simple physical calculations. Tectonics.

[CR56] Erne, S. (2014). *Temperaturabschätzung der Metasedimente zwischen Gotthard- und Aar-Massiv in der Urseren-Garvera Zone, mittels Ramanspektroskopie*. Bachelor thesis, unpublished, Universität Bern.

[CR57] Eugster HP (1951). Petrographische Untersuchungen im Gebiete der Val Russein (Aarmassiv-Ostende). Eclogae Geologicae Helvetiae.

[CR58] Fox M, Herman F, Willett SD, Schmid SM (2016). The Exhumation history of the European Alps inferred from linear inversion of thermochronometric data. American Journal of Science.

[CR59] Franks GD (1968). A study of upper Paleozoic sediments and volcanics in the northern part of the eastern Aar massif. Eclogae Geologicae Helvetiae.

[CR60] Franks GD (1968). The pre-Westphalian (Hercynian) metamorphism and structures of the Tödi area (Aar Massif). Schweizerische mineralogische und petrographische Mitteilungen.

[CR61] Frey M (1980). Very lowgrade metamorphism in external parts of the Central Alps: Illite crystallinity, coal rank and fluid inclusion data. Eclogae Geologicae Helvetiae.

[CR62] Frey M, Ferreiro Mählmann R (1999). Alpine metamorphism of the Central Alps. Schweizerische mineralogische und petrographische Mitteilungen.

[CR63] Frey M, Hunziker J, Frank W, Bocquet J, Dal Piaz G, Jäger E, Niggli E (1974). Alpine metamorphism of the alps: A review. Schweizerische mineralogische und petrographische Mitteilungen.

[CR64] Frey M, Jäger E, Niggli E (1976). Gesteinsmetamorphose im Bereich der Geotraverse Basel-Chiasso. Schweizerische mineralogische und petrographische Mitteilungen.

[CR65] Froitzheim N, Schmid SM, Frey M (1996). Mesozoic paleogeography and the timing of eclogite-facies metamorphism in the Alps: A working hypothesis. Eclogae Geologicae Helvetiae.

[CR66] Fügenschuh B, Schmid S (2003). Late stages of deformation and exhumation of an orogen constrained by fission-track data: A case study in the Western Alps. Geological Society of America Bulletin.

[CR67] Funk, H., Labhart, T., Milnes, A., Pfiffner, O.-A., Schaltegger, W., Schindler, C., Schmid, S., and Trümpy, R. (1983). Bericht über die Jubiläumsexkursion “Mechanismus der Gebirgsbildung” der Schweizerischen Geologischen Gesellschaft in das ost-und zentralschweizerische Helvetikum und in das nördliche Aarmassiv vom 12. bis 17. September 1982. *Eclogae Geologicae Helvetiae*, 76(1):91–123.

[CR68] Galy V, Beyssac O, France-Lanord C, Eglinton T (2008). Recycling of graphite during Himalayan erosion: A geological stabilization of carbon in the crust. Science.

[CR69] Gautschi, A., Schnellmann, M., Wilfried, A., Blümling, P., Vietor, T., Blaser, P., and Müller, H. (2008). Vorschlag geologischer Standortgebiete für das SMA- und das HAA-Lager: Geologische Grundlagen Beilagenband. In *Nagra Technischer Bericht, NTB 08-04*. Nagra, Wettingen.

[CR70] Girault J, Bellahsen N, Boutoux A, Rosenberg C, Nanni U, Verlaguet A, Beyssac O (2020). The 3-D Thermal Structure of the Helvetic Nappes of the European Alps: Implications for Collisional Processes. Tectonics.

[CR71] Gisler, C. (2018). Geological Atlas of Switzerland 1:25 000, map sheet 160 Amsteg, Explanatory notes. *Federal Office of Topography swisstopo, Wabern*.

[CR72] Gisler C, Hochuli PA, Ramseyer K, Bläsi H, Schlunegger F (2007). Sedimentological and palynological constraints on the basal Triassic sequence in Central Switzerland. Swiss Journal of Geosciences.

[CR73] Glotzbach C, Bernet M, van der Beek P (2011). Detrital thermochronology records changing source areas and steady exhumation in the Western European Alps. Geology.

[CR74] Glotzbach C, Reinecker J, Danišík M, Rahn MK, Frisch W, Spiegel C (2010). Thermal history of the central Gotthard and Aar massifs, European Alps: Evidence for steady state, long-term exhumation. Journal of Geophysical Research.

[CR75] Glotzbach C, van der Beek PA, Spiegel C (2011). Episodic exhumation and relief growth in the Mont Blanc massif, Western Alps from numerical modelling of thermochronology data. Earth and Planetary Science Letters.

[CR76] Goncalves P, Oliot E, Marquer D, Connolly J (2012). Role of chemical processes on shear zone formation: an example from the Grimsel metagranodiorite (Aar massif, Central Alps). Journal of Metamorphic Geology.

[CR77] Günzler-Seiffert H (1941). Persistente Brüche im Jura der Wildhorn-Decke des Berner Oberlandes. Eclogae Geologicae Helvetiae.

[CR78] Hafner, S. (2016). *The thermal peak in metamorphic black shales: Gradients in the Valaisan units and contrast to tectonic neighbors*. Master thesis, unpublished, Universität Bern.

[CR79] Handy MR, Schmid SM, Bousquet R, Kissling E, Bernoulli D (2010). Reconciling plate-tectonic reconstructions of Alpine Tethys with the geological–geophysical record of spreading and subduction in the Alps. Earth-Science Reviews.

[CR80] Hänni R, Pfiffner OA (2001). Evolution and internal structure of the Helvetic nappes in the Bernese Oberland. Eclogae Geologicae Helvetiae.

[CR81] Heim, A. (1887). Untersuchungen über den Mechanismus der Gebirgsbildung: Im Anschluss an die geologische Monographie der Tödi-Windgällen-Gruppe. *Schwabe, Basel*, 2 Bde. + Atlas.

[CR82] Heim A (1921). Geologie der Schweiz.

[CR83] Heim A, Heim A (1916). Die Juramulde bei Fernigen (Uri). Vjschr. natf. Ges. Zürich.

[CR84] Herb, R. (1983). Bericht über die Exkursion der Schweizerischen 100 Geologischen Gesellschaft auf das Schilthorn vom 19. September 1982. *Eclogae Geologicae Helvetiae*, 76(1):181–188.

[CR85] Herwegh M, Berger A, Baumberger R, Wehrens P, Kissling E (2017). Large-scale crustal-block-extrusion during late Alpine collision. Scientific Reports.

[CR86] Herwegh, M., Berger, A., Glotzbach, C., Wangenheim, C., Mock, S., Wehrens, P., Baumberger, R., Egli, D., and Kissling, E. (2020). Late stages of continent-continent collision: Timing, kinematic evolution, and exhumation of the northern rim (Aar Massif) of the Alps. *Earth-Science Reviews*, page 102959.

[CR87] Herwegh M, Pfiffner OA (2005). Tectono-metamorphic evolution of a nappe stack: A case study of the Swiss Alps. Tectonophysics.

[CR88] Hitz L, Pfiffner O-A (1994). A 3D crustal model of the Eastern External Aar Massif interpreted from a network of deep seismic profiles. Schweizerische mineralogische und petrographische Mitteilungen.

[CR89] Hofmann BA, Helfer M, Diamond LW, Villa IM, Frei R, Eikenberg J (2004). Topography-driven hydrothermal breccia mineralization of Pliocene age at Grimsel Pass, Aar massif, Central Swiss Alps. Schweizerische mineralogische und petrographische Mitteilungen.

[CR90] Hunziker JC, Frey M, Clauer N, Dallmeyer R, Friedrichsen H, Flehmig W, Hochstrasser K, Roggwiler PT, Schwander H (1986). The evolution of illite to muscovite: Mineralogical and isotopic data from the Glarus Alps, Switzerland. Contributions to Mineralogy and Petrology.

[CR91] Jackson J (1980). Reactivation of basement faults and crustal shortening in orogenic belts. Nature.

[CR92] Jammes S, Huismans RS (2012). Structural styles of mountain building: Controls of lithospheric rheologic stratification and extensional inheritance. Journal of Geophysical Research: Solid Earth.

[CR93] Janots E, Berger A, Gnos E, Whitehouse M, Lewin E, Pettke T (2012). Constraints on fluid evolution during metamorphism from U-Th-Pb systematics in Alpine hydrothermal monazite. Chemical Geology.

[CR94] Janots E, Rubatto D (2014). U-Th-Pb dating of collision in the external Alpine domains (Urseren zone, Switzerland) using low temperature allanite and monazite. Lithos.

[CR95] Käch, P. (1972). *Geologie der Brigelserhörner: (Bündnerisches Vorderrheintal)*. Dissertation, unpublished, ETH Zürich.

[CR96] Kammer, A. (1985). *Bau und Strukturen des noerdlichen Aarmassivs und seiner Sedimente zwischen dem Sustenpass und Grindelwald. (Berner Oberland)*. Dissertation, unpublished, Universität Bern.

[CR97] Kammer A (1989). Alpidische Verformung des aarmassivischen Nordrandes. Schweizerische mineralogische und petrographische Mitteilungen.

[CR98] Kedar L, Bond CE, Muirhead D (2020). Carbon ordering in an aseismic shear zone: Implications for raman geothermometry and strain tracking. Earth and Planetary Science Letters.

[CR99] Kempf O, Matter A, Burbank DW, Mange M (1999). Depositional and structural evolution of a foreland basin margin in a magnetostratigraphic framework: The eastern Swiss Molasse Basin. International Journal of Earth Sciences.

[CR100] Kempf O, Pfiffner O-A (2004). Early Tertiary evolution of the North Alpine Foreland Basin of the Swiss Alps and adjoining areas. Basin Research.

[CR101] Kirilova M, Toy V, Rooney JS, Giorgetti C, Gordon KC, Collettini C, Takeshita T (2018). Structural disorder of graphite and implications for graphite thermometry. Solid Earth.

[CR102] Krayenbuhl T, Steck A (2009). Structure and kinematics of the Jungfrau syncline, Faflertal (Valais, Alps), and its regional significance. Swiss Journal of Geosciences.

[CR103] Kuo L-W, Di Felice F, Spagnuolo E, Di Toro G, Song S-R, Aretusini S, Li H, Suppe J, Si J, Wen C-Y (2017). Fault gouge graphitization as evidence of past seismic slip. Geology.

[CR104] Labhart, T., Gisler, C., Renner, F., Schwizer, B., and Schaltegger, U. (2015). Geological Atlas of Switzerland 1:25 000, map sheet 146 Meiental, Explanatory notes. *Federal Office of Topography swisstopo, Wabern*.

[CR105] Labhart TP (1966). Mehrphasige alpine Tektonik am Nordrand des Aarmassivs Beobachtungen im Druckstollen Trift-Speicherberg (Gadmental) der Kraftwerke Oberhasli AG. Eclogae Geologicae Helvetiae.

[CR106] Labhart, T. P. (1977). Aarmassiv und Gotthardmassiv. In *Sammlung geologischer Führer*, volume 63. Schweizerbart’sche Verlagsbuchhandlung.

[CR107] Lacombe O, Bellahsen N (2016). Thick-skinned tectonics and basement-involved fold–thrust belts: Insights from selected Cenozoic orogens. Geological Magazine.

[CR108] Lacombe O, Mouthereau F (2002). Basement-involved shortening and deep detachment tectonics in forelands of orogens: Insights from recent collision belts (Taiwan, Western Alps, Pyrenees). Tectonics.

[CR109] Lafosse M, Boutoux A, Bellahsen N, Le Pourhiet L (2016). Role of tectonic burial and temperature on the inversion of inherited extensional basins during collision. Geological Magazine.

[CR110] Lahfid A, Beyssac O, Deville E, Negro F, Chopin C, Goffé B (2010). Evolution of the Raman spectrum of carbonaceous material in low-grade metasediments of the Glarus Alps (Switzerland). Terra Nova.

[CR111] Lehmann, A. (2008). *Geologie des hinteren Erstfeldertals*. Diploma thesis, unpublished, Universität Bern.

[CR112] Lemoine, M., Dardeau, G., Delpech, P.-Y., Dumont, T., De Graciansky, P.-C., Graham, R., Jolivet, L., Roberts, D., and Tricart, P. (1989). Extension synrift et failles transformantes jurassiques dans les Alpes occidentales. *Comptes rendus de l’Académie des sciences. Série 2, Mécanique, Physique, Chimie, Sciences de l’univers, Sciences de la Terre*, 309(17):1711–1716.

[CR113] Lihou J (1996). Stratigraphy and sedimentology of the Sardona unit, Glarus Alps: Upper Cretaceous middle Eocene deep-marine flysch sediments from the Ultrahelvetic realm. Eclogae Geologicae Helvetiae.

[CR114] Lihou J (1996). Structure and deformational history of the Infrahelvetic flysch units, Glarus Alps, eastern Switzerland. Eclogae Geologicae Helvetiae.

[CR115] Lihou JC, Allen PA (1996). Importance of inherited rift margin structures in the early North Alpine Foreland Basin Switzerland. Basin Research.

[CR116] Looser, N., Madritsch, H., Guillong, M., Laurent, O., Wohlwend, S., and Bernasconi, S. M. (2020). Absolute age and temperature constraints on faulting along the basal décollement of the jura fold-and-thrust belt from carbonate u-pb dating and clumped isotopes. *Earth and Space Science Open Archive ESSOAr*.

[CR117] Lu G, Winkler W, Rahn M, Von Quadt A, Willett SD (2018). Evaluating igneous sources of the Taveyannaz formation in the Central Alps by detrital zircon U-Pb age dating and geochemistry. Swiss Journal of Geosciences.

[CR118] Lünsdorf N, Lünsdorf J (2016). Evaluating Raman spectra of carbonaceous matter by automated, iterative curve-fitting. International Journal of Coal Geology.

[CR119] Lünsdorf NK, Dunkl I, Schmidt BC, Rantitsch G, von Eynatten H (2014). Towards a higher comparability of geothermometric data obtained by Raman spectroscopy of carbonaceous material. Part I: Evaluation of biasing factors. Geostandards and Geoanalytical Research.

[CR120] Lünsdorf NK, Dunkl I, Schmidt BC, Rantitsch G, von Eynatten H (2017). Towards a higher comparability of geothermometric data obtained by Raman spectroscopy of carbonaceous material. Part 2: A revised geothermometer. Geostandards and Geoanalytical Research.

[CR121] Mair D, Lechmann A, Herwegh M, Nibourel L, Schlunegger F (2018). Linking Alpine deformation in the Aar Massif basement and its cover units-the case of the Jungfrau-Eiger mountains (Central Alps, Switzerland). Solid Earth.

[CR122] Manatschal G (2004). New models for evolution of magma-poor rifted margins based on a review of data and concepts from West Iberia and the Alps. International Journal of Earth Sciences.

[CR123] Mancktelow NS, Grasemann B (1997). Time-dependent effects of heat advection and topography on cooling histories during erosion. Tectonophysics.

[CR124] Marshak S, Karlstrom K, Timmons JM (2000). Inversion of Proterozoic extensional faults: An explanation for the pattern of Laramide and Ancestral Rockies intracratonic deformation. United States. Geology.

[CR125] Maxelon M, Mancktelow NS (2005). Three-dimensional geometry and tectonostratigraphy of the Pennine zone, Central Alps. Switzerland and Northern Italy. Earth-Science Reviews.

[CR126] Menkveld-Gfeller U, Kempf O, Funk H (2016). Lithostratigraphic units of the Helvetic Palaeogene: Review, new definition, new classification. Swiss Journal of Geosciences.

[CR127] Michalski I, Soom M (1990). The Alpine thermo-tectonic evolution of the Aar and Gotthard massifs, Central Switzerland: Fission Track ages on zircon and apatite and K-Ar mica ages. Schweizerische mineralogische und petrographische Mitteilungen.

[CR128] Milnes AG (1974). Post-nappe folding in the western Lepontine Alps. Eclogae Geologicae Helvetiae.

[CR129] Milnes AG, Pfiffner O-A (1977). Structural development of the Infrahelvetic complex, eastern Switzerland. Eclogae Geologicae Helvetiae.

[CR130] Mock, S. (2014). *Deformation of the sediment-crystalline contact in the northern Aar Massif (Innertkirchen, Bernese Oberland)*. Master thesis, unpublished, Universität Bern.

[CR131] Mock S, Herwegh M (2017). Tectonics of the central Swiss Molasse Basin: Post-Miocene transition to incipient thick-skinned tectonics?. Tectonics.

[CR132] Mock S, von Hagke C, Schlunegger F, Dunkl I, Herwegh M (2020). Long-wavelength late-miocene thrusting in the north alpine foreland: Implications for late orogenic processes. Solid Earth.

[CR133] Mohn G, Manatschal G, Beltrando M, Masini E, Kusznir N (2012). Necking of continental crust in magma-poor rifted margins: Evidence from the fossil Alpine Tethys margins. Tectonics.

[CR134] Molnar P, England P (1990). Temperatures, heat flux, and frictional stress near major thrust faults. Journal of Geophysical Research: Solid Earth.

[CR135] Morgenthaler H (1921). Petrographisch-tektonische Untersuchungen am Nordrand des Aarmassivs. AAPG Bulletin.

[CR136] Mouthereau F, Watts AB, Burov E (2013). Structure of orogenic belts controlled by lithosphere age. Nature geoscience.

[CR137] Negro F, Bousquet R, Vils F, Pellet C-M, Hänggi-Schaub J (2013). Thermal structure and metamorphic evolution of the Piemont-Ligurian metasediments in the northern Western Alps. Swiss Journal of Geosciences.

[CR138] Nemčok M, Mora A, Cosgrove J (2013). Thick-skin-dominated orogens; from initial inversion to full accretion: An introduction. Geological society, London, Special Publications.

[CR139] Nibourel, L. (2019). *The structural and thermo-kinematic evolution of the eastern Aar Massif, Switzerland*. Ph.D. thesis, unpublished, Universität Bern.

[CR140] Nibourel L, Berger A, Egli D, Luensdorf NK, Herwegh M (2018). Large vertical displacements of a crystalline massif recorded by Raman thermometry. Geology.

[CR141] Niggli E, Niggli C (1965). Karten der Verbreitung einiger Mineralien der alpidischen Metamorphose in den Schweizer Alpen (Stilpnomelan, Alkali-Amphibol, Chloritoid, Staurolith, Disthen, Sillimanit). Eclogae Geologicae Helvetiae.

[CR142] Oberhänsli R, Schenker F, Mercolli I (1988). Indications of Variscan nappe tectonics in the Aar Massif. Schweizerische mineralogische und petrographische Mitteilungen.

[CR143] Oliot E, Goncalves P, Marquer D (2010). Role of plagioclase and reaction softening in a metagranite shear zone at mid-crustal conditions (Gotthard Massif, Swiss Central Alps). Journal of Metamorphic Geology.

[CR144] Pfiffner O-A (1972). Neue Kenntnisse zur Geologie östlich und westlich des Kunkelspasses (GR). Eclogae Geologicae Helvetiae.

[CR145] Pfiffner O-A (1978). Der Falten-und Kleindeckenbau im infrahelvetikum der Ostschweiz. Eclogae Geologicae Helvetiae.

[CR146] Pfiffner OA (1993). The structure of the Helvetic nappes and its relation to the mechanical stratigraphy. Journal of Structural Geology.

[CR147] Pfiffner, O. A., Ramsey, J.G., & Schmid, S.M. (2011). Structural map of the Helvetic Zone of the Swiss Alps, including Vorarlberg (Austria) and Haute Savoie (France), 128, 1:100 000, Explanatory notes. *Special Geological Maps, Federal Office of Topography swisstopo, Wabern*.

[CR148] Pfiffner, O. A. (2015). *Geologie der Alpen*. Haupt, Bern, Stuttgart, Wien, 3 edition.

[CR149] Pfiffner OA (2017). Thick-skinned and thin-skinned tectonics: A global perspective. Geosciences.

[CR150] Pfiffner OA, Frei W, Valasek P, Stäuble M, Levato L, DuBois L, Schmid SM, Smithson SB (1990). Crustal shortening in the Alpine Orogen: Results from deep seismic reflection profiling in the eastern Swiss Alps, Line NFP 20-east. Tectonics.

[CR151] Pfiffner, O. A., Lehner, P., Heitzmann, P., Mueller, S., and Steck, A. (1997). *Deep structure of the Swiss Alps: results of NRP 20*. Birkhäuser.

[CR152] Pleuger J, Mancktelow N, Zwingmann H, Manser M (2012). K-Ar dating of synkinematic clay gouges from Neoalpine faults of the Central, Western and Eastern Alps. Tectonophysics.

[CR153] Rahn M, Grasemann B (1999). Fission track and numerical thermal modeling of differential exhumation of the Glarus thrust plane (Switzerland). Earth and Planetary Science Letters.

[CR154] Rahn M, Mullis J, Erdelbrock K, Frey M (1995). Alpine metamorphism in the north Helvetic flysch of the Glarus-Alps, Switzerland. Eclogae Geologicae Helvetiae.

[CR155] Rahn M, Selbekk R (2007). Absolute dating of the youngest sediments of the Swiss Molasse basin by apatite fission track analysis. Swiss Journal of Geosciences.

[CR156] Ramsay JG, Casey M, Kligfield R (1983). Role of shear in development of the Helvetic fold-thrust belt of Switzerland. Geology.

[CR157] Ramsay, J. G. and Huber, M. I. (1987). *The techniques of modern structural geology. Vol. 2: Folds and fractures*. Academic Press.

[CR158] Rauchenstein-Martinek, K. (2014). *Metamorphic fluid history along a cross section through the Central Alps: Constraints from LA-ICPMS analysis of fluid inclusions and Ar-Ar geochronology*. PhD thesis, unpublished, ETH Zürich.

[CR159] Reinecker J, Danišík M, Schmid C, Glotzbach C, Rahn MK, Frisch W, Spiegel C (2008). Tectonic control on the late stage exhumation of the Aar Massif (Switzerland): Constraints from apatite fission track and (U-Th)/He data. Tectonics.

[CR160] Ricchi E, Bergemann C, Gnos E, Berger A, Rubatto D, Whitehouse M (2019). Constraining deformation phases in the Aar Massif and the Gotthard Nappe (Switzerland) using Th-Pb crystallization ages of fissure monazite-(Ce). Lithos.

[CR161] Rodgers J (1949). Evolution of thought on structure of middle and southern Appalachians. AAPG Bulletin.

[CR162] Rohr, K. (1926). Stratigraphische und tektonische Untersuchungen der Zwischenbildungen am Nordrand des Aarmassivs (zwischen Wendenjoch und Wetterhorn). *Beiträge zur Geologischen Karte der Schweiz*, NF 57.

[CR163] Rolland Y, Cox SF, Corsini M (2009). Constraining deformation stages in brittle-ductile shear zones from combined field mapping and 40Ar/39Ar dating: The structural evolution of the Grimsel Pass area (Aar Massif, Swiss Alps). Journal of Structural Geology.

[CR164] Rolland Y, Rossi M, Cox S, Corsini M, Mancktelow N, Pennacchioni G, Fornari M, Boullier A-M (2008). 40Ar/39Ar dating of synkinematic white mica: Insights from fluid-rock reaction in low-grade shear zones (Mont Blanc Massif) and constraints on timing of deformation in the NW external Alps. Geological Society, London, Special Publications.

[CR165] Rosenberg CL, Berger A, Bellahsen N, Bousquet R (2015). Relating orogen width to shortening, erosion, and exhumation during Alpine collision. Tectonics.

[CR166] Rosenberg CL, Kissling E (2013). Three-dimensional insight into Central-Alpine collision: Lower-plate or upper-plate indentation?. Geology.

[CR167] Sanchez G, Rolland Y, Jolivet M, Brichau S, Corsini M, Carter A (2011). Exhumation controlled by transcurrent tectonics: The Argentera-Mercantour massif (SW Alps). Terra Nova.

[CR168] Schaltegger U, Abrecht J, Corfu F (2003). The Ordovician orogeny in the Alpine basement: Constraints from geochronology and geochemistry in the Aar Massif (Central Alps). Swiss Bulletin of Mineralogy and Petrology.

[CR169] Schenker, F. (1980). *Geologische und Mineralogische Untersuchungen im Gebiet der kleinen und grossen Windgälle, Maderanertal, Kanton Uri*. Lizentiatsarbeit, unpublished, Universität Bern.

[CR170] Schenker F (1987). Hinweise für kompressive Tektonik während der Ablagerung von oberpaläozoischen Sedimenten und Vulkaniten im Aarmassiv. Bulletin der Vereinigung Schweiz. Petroleum-Geologen u. -Ingenieure.

[CR171] Schenker F, Abrecht J (1987). Prä-aargranitische Anatexis, variszische Kontaktmetamorphose und alpidische Regionalmetamorphose im Oberhasli (zentrales Aarmassiv, Schweiz). Schweizerische mineralogische und petrographische Mitteilungen.

[CR172] Schlunegger F, Matter A, Burbank DW, Klaper EM (1997). Magnetostratigraphic constraints on relationships between evolution of the central Swiss Molasse basin and Alpine orogenic events. Geological Society of America Bulletin.

[CR173] Schmid SM, Fügenschuh B, Kissling E, Schuster R (2004). Tectonic map and overall architecture of the Alpine orogen. Eclogae Geologicae Helvetiae.

[CR174] Schmid SM, Kissling E, Diehl T, van Hinsbergen DJJ, Molli G (2017). Ivrea mantle wedge, arc of the Western Alps, and kinematic evolution of the Alps-Apennines orogenic system. Swiss Journal of Geosciences.

[CR175] Schmid SM, Pfiffner OA, Froitzheim N, Schönborn G, Kissling E (1996). Geophysical-geological transect and tectonic evolution of the Swiss-Italian Alps. Tectonics.

[CR176] Schneeberger R, de La Varga M, Egli D, Berger A, Kober F, Wellmann F, Herwegh M (2017). Methods and uncertainty estimations of 3-d structural modelling in crystalline rocks: a case study. Solid Earth.

[CR177] Schneeberger R, Egli D, Lanyon GW, Mäder UK, Berger A, Kober F, Herwegh M (2018). Structural-permeability favorability in crystalline rocks and implications for groundwater flow paths: A case study from the Aar Massif (central Switzerland). Hydrogeology Journal.

[CR178] Schwartz S, Gautheron C, Audin L, Dumont T, Nomade J, Barbarand J, Pinna-Jamme R, van der Beek P (2017). Foreland exhumation controlled by crustal thickening in the Western Alps. Geology.

[CR179] Shi Y, Wang C-Y (1987). Two-dimensional modeling of the p-t-t paths of regional metamorphism in simple overthrust terrains. Geology.

[CR180] Spillmann, P. (2011). *Geologie des Kantons Uri*. Naturforschende Gesellschaft Uri.

[CR181] Steck A (1968). Die alpidischen Strukturen in den Zentralen Aaregraniten des westlichen Aarmassivs. Eclogae Geologicae Helvetiae.

[CR182] Steck A (1984). Structures et deformations tertiaires dans les Alpes Centrales (transversale Aar-Simplon-Ossola). Eclogae Geologicae Helvetiae.

[CR183] Tan, B. (1969). *Analysis of Tectonic Strain in Windgällen and Färnigen, Canton Uri, Switzerland*. Ph.D. thesis, unpublished, University of London.

[CR184] Tan B (1976). Oolite deformation in Windgällen, Canton Uri, Switzerland. Tectonophysics.

[CR185] Torgersen, E., Bingen, B., and Ganerød, M. (2018). Tectonic basement slices in the décollement of the Scandinavian Caledonides: Transition from thin-skinned to thick-skinned tectonics in collisional orogens. In *EGU General Assembly Conference Abstracts*, volume 20, page 17048.

[CR186] Tricart P, Lemoine M (1986). From faulted blocks to megamullions and megaboudins: Tethyan heritage in the structure of the Western Alps. Tectonics.

[CR187] Trümpy, R., Aubert, D., and Bernoulli, D. (1980). *Geology of Switzerland: Geological excursions*, volume 10. Wepf.

[CR188] Tuinstra F, Koenig JL (1970). Raman spectrum of graphite. The Journal of Chemical Physics.

[CR189] Vernon AJ, van der Beek PA, Sinclair HD, Persano C, Foeken J, Stuart FM (2009). Variable late Neogene exhumation of the central European Alps: Low-temperature thermochronology from the Aar Massif, Switzerland, and the Lepontine Dome. Italy. Tectonics.

[CR190] Vernon AJ, van der Beek PA, Sinclair HD, Rahn MK (2008). Increase in late Neogene denudation of the European Alps confirmed by analysis of a fission-track thermochronology database. Earth and Planetary Science Letters.

[CR191] Voll G (1976). Recrystallization of quartz, biotite and feldspars from Erstfeld to the Leventina nappe, Swiss Alps, and its geological significance. Schweizerische mineralogische und petrographische Mitteilungen.

[CR192] von Hagke C, Cederbom CE, Oncken O, Stöckli DF, Rahn MK, Schlunegger F (2012). Linking the northern Alps with their foreland: The latest exhumation history resolved by low-temperature thermochronology. Tectonics.

[CR193] von Hagke C, Oncken O, Ortner H, Cederbom CE, Aichholzer S (2014). Late Miocene to present deformation and erosion of the Central Alps—Evidence for steady state mountain building from thermokinematic data. Tectonophysics.

[CR194] Von Raumer, J., Ménot, R., Abrecht, J., and Biino, G. (1993). The Pre-Alpine evolution of the External massifs. In *Pre-mesozoic geology in the Alps*, pages 221–240. Springer.

[CR195] Wagner M, Kissling E, Husen S (2012). Combining controlled-source seismology and local earthquake tomography to derive a 3-D crustal model of the western Alpine region. Geophysical Journal International.

[CR196] Wangenheim, C. (2016). *Quantifying Fluvial and Glacial Erosion Using (detrital) Thermochronology, Cosmogenic Nuclides and Numerical Modelling: A Case Study in the European Alps*. Ph.D. thesis, unpublished, Gottfried Wilhelm Leibniz Universität Hannover.

[CR197] Wehrens P, Baumberger R, Berger A, Herwegh M (2017). How is strain localized in a meta-granitoid, mid-crustal basement section? Spatial distribution of deformation in the central Aar massif (Switzerland). Journal of Structural Geology.

[CR198] Wehrens P, Berger A, Peters M, Spillmann T, Herwegh M (2016). Deformation at the frictional-viscous transition: Evidence for cycles of fluid-assisted embrittlement and ductile deformation in the granitoid crust. Tectonophysics.

[CR199] Wiederkehr M, Bousquet R, Schmid SM, Berger A (2008). From subduction to collision: Thermal overprint of HP/LT meta-sediments in the north-eastern Lepontine Dome (Swiss Alps) and consequences regarding the tectono-metamorphic evolution of the Alpine orogenic wedge. Swiss Journal of Geosciences.

[CR200] Wiederkehr M, Bousquet R, Ziemann MA, Berger A, Schmid SM (2011). 3-D assessment of peak-metamorphic conditions by Raman spectroscopy of carbonaceous material: An example from the margin of the Lepontine dome (Swiss Central Alps). International Journal of Earth Sciences.

[CR201] Wyss R (1986). Die Urseren-Zone: Lithostratigraphie und Tektonik. Eclogae Geologicae Helvetiae.

