# Supplementary material for: Structural and thermal evolution of the eastern Aar Massif: insights from structural field work and Raman thermometry
Source: Swiss J Geosci. 2021 Mar 3;114(1):9. doi: 10.1186/s00015-020-00381-3 (PMC7929973; doi:10.1186/s00015-020-00381-3)
Supplement: Supplementary file 1 — Additional file 1: Table S1 contains a list of geological maps and cross sections used for the shear zone map shown on Figure 2a. Table S2 contains RSCM data and sample information. Figures S1–S4 contain histograms showing the distribution of RSCM peak temperature data for each sample. [file 15_2020_381_MOESM1_ESM.pdf]

Supporting information for "Structural and thermal evolution of the eastern Aar Massif: insights from structural field work and Raman thermometry"

Lukas Nibourel[1,2], Alfons Berger[1], Daniel Egli[1], Stefan Heuberger[2] and Marco Herwegh[1]

Contents of this file

1 Figures S1 to S4
2 Tables S1 and S2

Table S1 List of geological maps and cross sections used for the shear zone map (Figure 2A of the main manuscript) and for the construction of cross sections A-A‴, B-B‴ and C-C′ (Figures 1, 3 and 19 of main manuscript).

Data type	Source
Geological Atlas of Switzerland 1:25'000	Swiss Geological Survey of the Federal Office of Topography (swisstopo); Map sheets: 83, 126, 133, 146
Special Geological Maps 1:100'000	Swiss Geological Survey of the Federal Office of Topography (swisstopo); Map sheet: 128
Tectonic map of Switzerland 1:500'000	Swiss Geological Survey of the Federal Office of Topography (swisstopo)
Geological vector dataset GeoCover	Swiss Geological Survey of the Federal Office of Topography (swisstopo)
Geological cross-sections	Böhm (1986); Brückner and Zbinden (1987); Funk et al. (1983); Käch (1972); Pfiffner (1978, 1985, 2011, 2017); Pfiffner et al. (1997)
Geological and tectonic maps and sketches	Baumberger (2015); Gnos (1988); Lehmann (2008); Wehrens et al. (2017)
Gotthard base tunnel (NEAT) geological profiles and maps	AlpTransit Gotthard AG; Report Nr. 4343-5; 4343-13; 4343-72; 4343-81 (unpublished)
Hydropower gallery geological profiles	Kraftwerke Amsteg, Wassen and Göschenen (unpublished)
Basal Helvetic thrust contour map	Schmid (1975),
Top basement topography map	For latest version see Pfiffner (2011)

Table S2 RSCM data and sample information.

Sample number	Location			Tp	SD	Error	N	Curve-fitting method	Stratigraphic unit	Tectonic unit
	Lat (°N)	Long (°E)	Elevation (m)	(°C)	(°C)	(°C)				
009	8.74957	46.7525	2300	443**	15	40	29	Lu	Permo-Carboniferous	LH
010	8.682062	46.75964	1520	345*	32	40	11	Lu	Permo-Carboniferous	LH
011	8.736525	46.79781	2440	316	5	40	12	Lu	Cenozoic	LH
ER-15-04	8.623024	46.84079	700	275	13	40	24	Lu	Middle Jurassic	LH
EZ-15-02	8.75187	46.72394	2290	517**	26	40	17	Lu	Permo-Carboniferous	LH
EZ-15-05	8.707744	46.72341	2430	497**	84	40	22	Lu	Permo-Carboniferous	LH
MA-15-01	8.803064	46.79838	1500	325	16	40	20	Lu	Middle Jurassic	LH
TO-15-02	8.925855	46.83	2370	326	8	40	22	Lu	Middle Jurassic	LH
TO-15-03	8.937819	46.8289	2250	302	13	40	10	Lu	Permo-Carboniferous	LH
KL-15-01	8.87815	46.86422	1780	280	51	40	25	Lu	Cenozoic	LH
KL-15-04	8.854764	46.87174	2100	279	9	40	24	Lu	Lower Jurassic	HN
KL-15-06	8.725604	46.86983	850	272	11	40	26	Lu	Cenozoic	LH
AL-15-01	8.611384	46.8746	450	244	12	40	21	Lu	Cenozoic	LH
DI-15-01	8.859183	46.7078	1150	404	27	40	16	Lu	Middle Jurassic	LH
RU-15-02	8.896664	46.81411	2850	324	7	40	17	Lu	Permo-Carboniferous	LH
RU-15-03	8.897966	46.8143	2900	320	11	40	14	Lu	Middle Jurassic	LH
FR-15-02	9.061763	46.7771	1320	359	15	40	9	Lu	Middle Jurassic	LH
TR-15-01A	8.975203	46.74206	950	422**	12	40	6	Lu	Middle Jurassic	LH
TR-15-01B	8.975203	46.74206	950	419**	18	40	19	Lu	Middle Jurassic	LH
HU-16-03	8.81873	46.80184	2320	327	10	40	20	Lu	Middle Jurassic	LH
GL-16-02	8.92239	46.78183	2490	540**	11	40	8	Lu	Late Paleozoic?	LH
GL-16-03	8.93166	46.78692	2800	322	13	40	15	Lu	Middle Jurassic	LH
GL-16-07	8.901626	46.77055	2605	560**	22	40	7	Lu	Late Paleozoic?	LH
GL-16-14	8.929083	46.78846	2870	351	18	40	19	Lu	Upper Jurassic	LH
WI-16-15	8.774771	46.81014	3137	315	9	40	20	Lu	Upper Jurassic	LH
WI-16-16	8.790627	46.81905	2611	286	14	40	16	Lu	Tertiary sediments	LH
DA-17-01	9.006728	46.78825	2772	344	18	40	13	Lu	Upper Jurassic	LH
DA-17-02	8.987096	46.78974	2640	345	19	40	7	Lu	Middle Jurassic	LH
SI-17-02	8.690911	46.79905	1550	290	17	40	13	Lu	Upper Jurassic	LH
SI-17-03	8.697856	46.8036	2060	292	6	40	13	Lu	Cenozoic	LH
SI-17-04	8.706082	46.80076	2280	305	14	40	16	Lu	Cenozoic	LH
SI-17-05	8.681911	46.8195	1520	293	8	40	15	Lu	Middle Jurassic	LH
UR-17-01A	8.168031	46.65294	2700	310	10	40	14	Lu	Middle Jurassic	LH
UR-17-01B	8.168031	46.65294	2695	303	14	40	6	Lu	Upper Jurassic	LH
UR-17-01C	8.168031	46.65294	2690	314	15	40	11	Lu	Upper Jurassic	LH
UR-17-02	8.177011	46.65712	2220	302	15	40	11	Lu	Upper Jurassic	LH
UR-17-03	8.184863	46.65998	2000	304	11	40	13	Lu	Upper Jurassic	LH
UR-17-04	8.186882	46.65948	1890	306	13	40	10	Lu	Upper Jurassic	LH
UR-17-05	8.197834	46.67087	1050	293	5	40	12	Lu	Middle Jurassic	LH
UR-17-06	8.227632	46.70843	622	308	10	40	13	Lu	Middle Jurassic	LH
DL V5	9.094209	46.9393	2050	244	13	40	12	Lu	Permo-Carboniferous	HN
IN22	8.289267	46.70756	1520	306	13	40	15	Lu	Middle Jurassic	LH
BL03	8.291643	46.69076	2222	385**	69	40	30	Lu	Late Paleozoic?	LH
BL05-1	8.290781	46.68999	2164	325	16	40	14	Lu	Permo-Carboniferous	LH
TR-17-01	8.983025	46.77751	2530	334	11	40	13	Lu	Middle Jurassic	LH
TR-17-02	8.98391	46.77687	2460	340	38	40	19	Lu	Triassic (dolomite)	LH
SM-17-01	8.934376	46.72767	1090	358	21	40	23	Lu	Lower Jurassic	LH

Samples from this study are stored in the collections of the University of Bern, Institute of Geological Sciences, Switzerland. T_p = RSCM peak temperature, SD = standard deviation, Error = temperature calibration based error, N = number of considered spectra, Lu = Lünsdorf et al. (2017), LH = Lower Helvetic units, HN = Upper Helvetic nappes, * Based on a local maximum (bimodal or more complex temperature distribution), ** discarded T_p value (per-Alpine temperature or clear outlier, see discussion below).

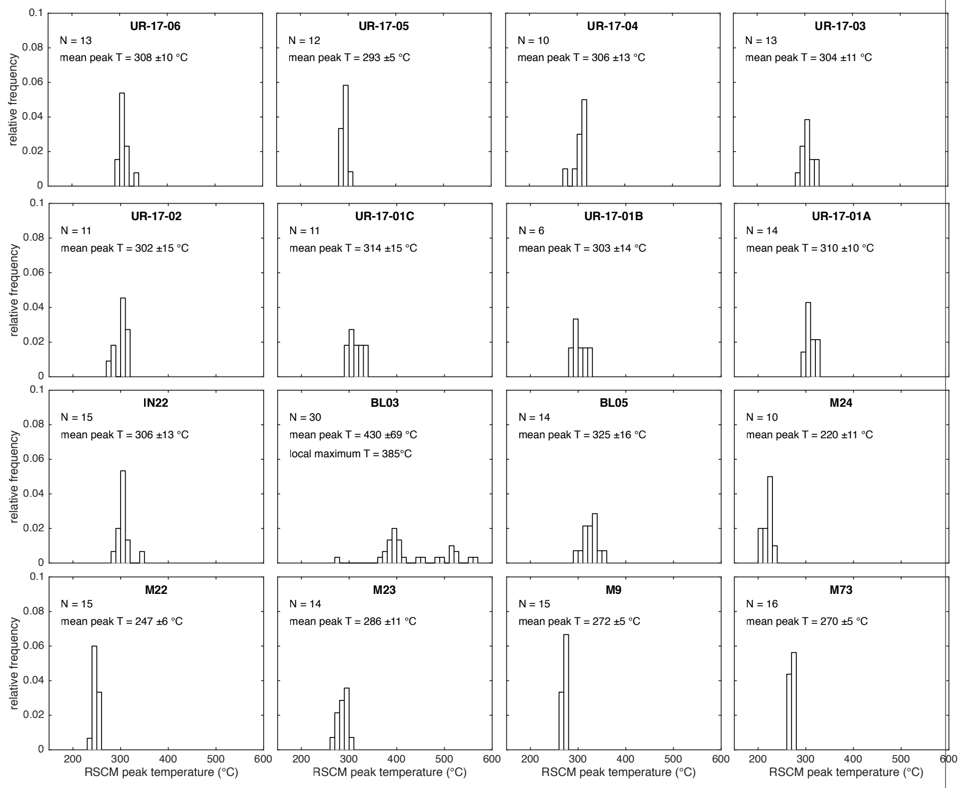

Figure S1 Histograms showing the distribution of RSCM peak temperature data for each sample. Histograms to samples from Nibourel et al. (2018) are also shown. N = number of considered spectra.

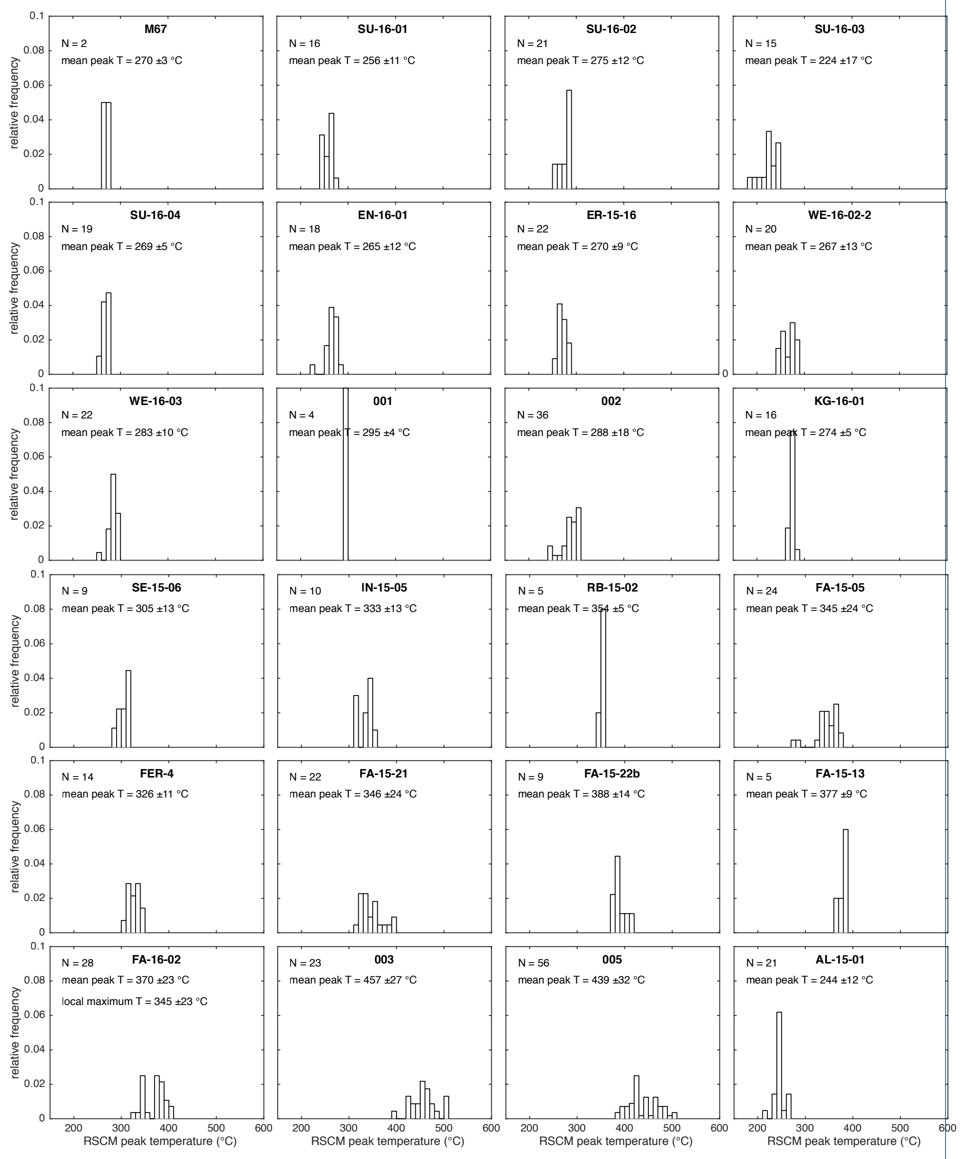

Figure S2 Histograms showing the distribution of RSCM peak temperature data for each sample. Histograms to samples from Nibourel et al. (2018) are also shown. N = number of considered spectra.

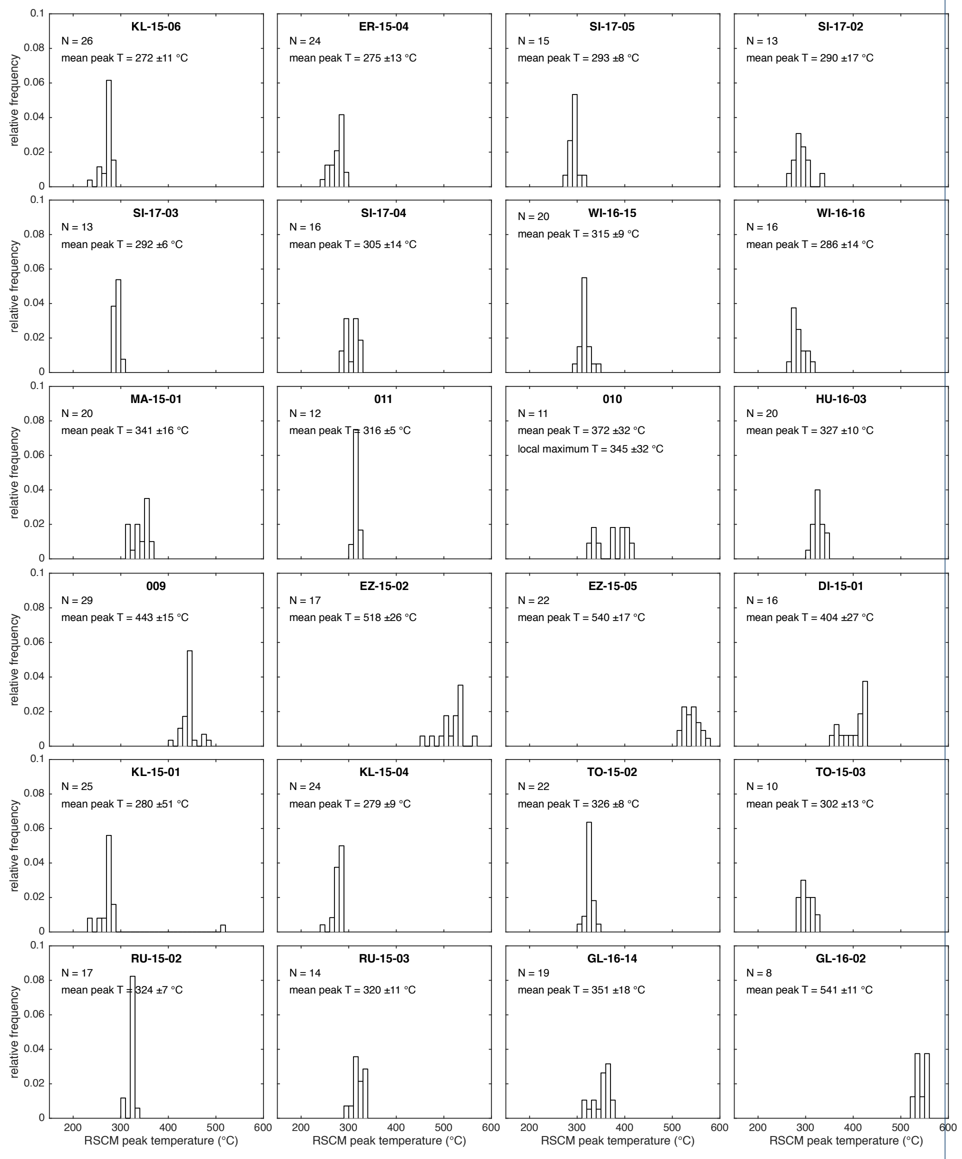

Figure S3 Histograms showing the distribution of RSCM peak temperature data for each sample. Histograms to samples from Nibourel et al. (2018) are also shown. N = number of considered spectra.

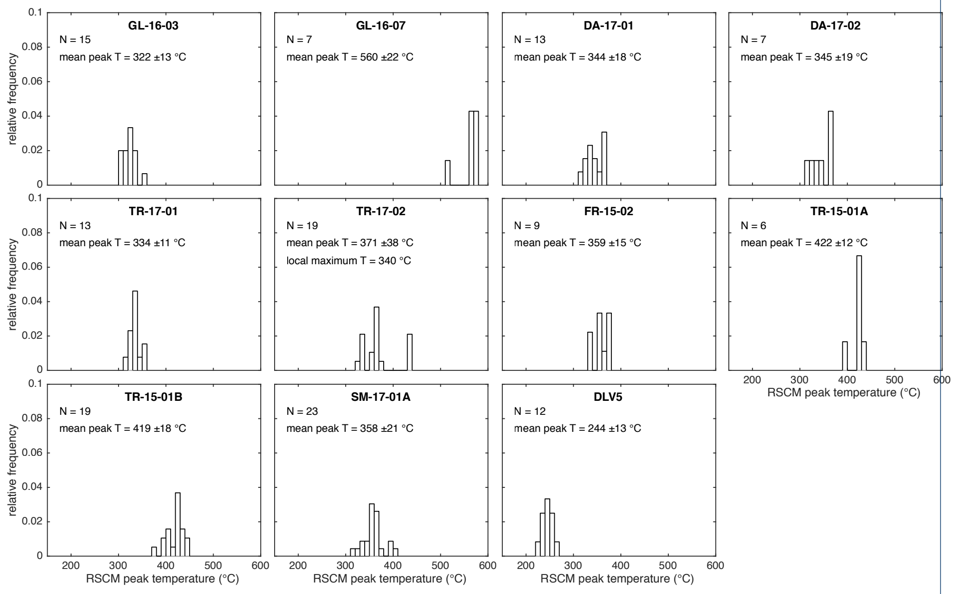

Figure S4 Histograms showing the distribution of RSCM peak temperature data for each sample. Histograms to samples from Nibourel et al. (2018) are also shown. N = number of considered spectra.

Author details

[1]Institute of Geological Sciences, University of Bern, Baltzerstrasse 1+3, 3012 Bern, Switzerland. [2]Department of Earth Sciences, ETH Zurich, Sonneggstrasse 5, 8092 Zürich, Switzerland.

References

Baumberger, R. (2015). *Quantification of lineaments: Link between internal 3D structure and surface evolution of the Hasli valley (Aar Massif, Central Alps, Switzerland)*. PhD thesis, unpublished, Universität Bern.

Böhm, C. (1986). *Geologie und Petrographie im Gebiet von Val Russein und Val Gliems, Graubünden*. Diplomarbeit, unpublished, Universität Bern.

Brückner, W. and Zbinden, P. (1987). Geological Atlas of Switzerland 1:25 000, map sheet 83 Schächental, Explanatory notes. *Federal Office of Topography swisstopo, Wabern*.

Funk, H., Labhart, T., Milnes, A., Pfiffner, O.-A., Schaltegger, W., Schindler, C., Schmid, S., and Trümpy, R. (1983). Bericht über die Jubiläumsexkursion "Mechanismus der Gebirgsbildung" der Schweizerischen Geologischen Gesellschaft in das ost-und zentralschweizerische Helvetikum und in das nördliche Aarmassiv vom 12. bis 17. September 1982. *Eclogae Geologicae Helvetiae*, 76(1):91–123.

Gnos, E. (1988). *Geologie und petrographie des westlichen Brunnitals, Maderanertal, Kanton Uri*. Diplomarbeit, unpublished, Universität Bern.

Käch, P. (1972). *Geologie der Brigelserhörner: (Bündnerisches Vorderrheintal)*. Dissertation, unpublished, ETH Zürich.

Lehmann, A. (2008). *Geologie des hinteren Erstfeldertals*. Diploma thesis, unpublished, Universität Bern.

Lünsdorf, N. K., Dunkl, I., Schmidt, B. C., Rantitsch, G., and von Eynatten, H. (2017). Towards a higher comparability of geothermometric data obtained by Raman spectroscopy of carbonaceous material. Part 2: a revised geothermometer. *Geostandards and Geoanalytical Research*, 41(4):593–612.

Nibourel, L., Berger, A., Egli, D., Luensdorf, N. K., and Herwegh, M. (2018). Large vertical displacements of a crystalline massif recorded by Raman thermometry. *Geology*, 46(10):879–882.

Pfiffner, O.-A. (1978). Der Falten-und Kleindeckenbau im infrahelvetikum der Ostschweiz. *Eclogae Geologicae Helvetiae*, 71(1):61–84.

Pfiffner, O. A. (1985). Displacements along thrust faults. *Eclogae Geologicae Helvetiae*, 78(2):313–333.

Pfiffner, O. A. (2011). Structural Map of the Helvetic Zone of the Swiss Alps, including Vorarlberg (Austria) and Haute Savoie (France), 128, 1:100 000, Explanatory notes. *Special Geological Maps, Federal Office of Topography swisstopo, Wabern*.

Pfiffner, O. A. (2017). Thick-skinned and thin-skinned tectonics: A global perspective. *Geosciences*, 7(3):71.

Pfiffner, O. A., Lehner, P., Heitzmann, P., Mueller, S., and Steck, A. (1997). *Deep structure of the Swiss Alps: results of NRP 20*. Birkhäuser.

Schmid, S. M. (1975). The Glarus overthrust: Field evidence and mechanical model. *Eclogae Geologicae Helvetiae*, 68:247–280.

Wehrens, P., Baumberger, R., Berger, A., and Herwegh, M. (2017). How is strain localized in a meta-granitoid, mid-crustal basement section? Spatial distribution of deformation in the central Aar massif (Switzerland). *Journal of Structural Geology*, 94:47–67.
